# Elementary Steps in Olefin Metathesis: Nickelacyclobutanes via Cycloaddition to Nickel Carbenes

**DOI:** 10.1021/jacs.5c06505

**Published:** 2025-07-07

**Authors:** Samantha K. Cormier, Marco Foscato, Michael J. Ferguson, Vidar R. Jensen, R. Tom Baker, Deryn E. Fogg

**Affiliations:** † Centre for Catalysis Research & Innovation and Department of Chemistry and Biomolecular Sciences, 6363 University of Ottawa, Ottawa, ON K1N 6N5, Canada; ‡ Department of Chemistry, University of Bergen, Allégaten 41, N-5007, Bergen, Norway; § X-ray Crystallography Laboratory, 3158 University of Alberta, 11227 Saskatchewan Dr., Edmonton, AB T6G 2G2, Canada

## Abstract

Olefin metathesis and cyclopropanation, major reactions in the synthetic toolbox, are predominantly catalyzed by platinum-group metals. First-row transition metal alternatives are desirable from the perspective of supply-chain sustainability. Although cyclopropanation by nickel catalysts is well established, limited evidence exists for the chemical feasibility of the cycloaddition step essential for inner-sphere cyclopropanation or olefin metathesis. We describe Ni­(PP)­(=CPh_2_) complexes that provide this missing evidence. These complexes react with styrene and phenyl vinyl sulfone (PVS) to form β-H elimination products that imply nickelacyclobutane (NiCB) intermediates. For the PVS system, the NiCB could be observed directly by NMR analysis and X-ray crystallography. This is the sole example to date of an obervable NiCB generated via cycloaddition, beyond the fluorinated examples. Computations pinpoint a decreased energy barrier to cycloreversion, relative to reductive elimination or β-H elimination, as the core requirement to achieve metathesis. This work thus demonstrates that nickel complexes can engage in cycloaddition, the missing elementary step essential for olefin metathesis, and establishes the design parameters essential to close the catalytic cycle.

## Graphical abstract

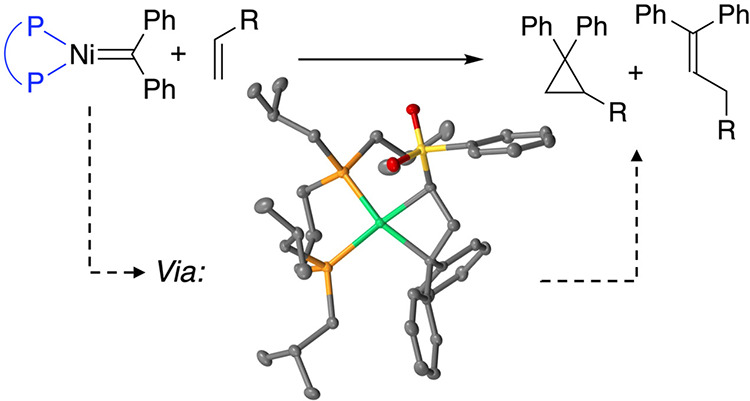

Cyclopropanation^1^ and olefin metathesis,^2^ divergent catalytic manifolds enabled by a common set of transition-metal species, hold high value in synthetic chemistry.^3^ Metal carbene (alkylidene) complexes,^4–7^ typically of platinum-group metals,^8^ play a central role in both reaction classes. Concerns about supply-chain security, exacerbated by geopolitical tensions, are reinforcing efforts to develop base-metal alternatives.^9^ Among first-row transition metal candidates, nickel is attractive for its abundance and the accessibility of major reserves worldwide. Likewise compelling are its diverse reactivity, which offers potential access to new chemical space,^10^ and suggestions that nickel catalysts may be tuned to favor two-electron chemistry and to inhibit β-H elimination.^11^

In principle, both olefin metathesis and cyclopropanation may be mediated by [Ni]=CRR’ complexes, if the latter are able to form nickelacyclobutanes (NiCBs; Scheme 1a) via inner-sphere olefin cycloaddition. Retro-addition then effects olefin exchange; reductive elimination liberates a cyclopropane and a reduced Ni species. Olefin cyclopropanation may also proceed via outer-sphere insertion (Scheme 1b), without NiCB intermediates. In either case, cyclopropanation is stoichiometric in the absence of an exogenous carbene source, whereas metathesis can be fully catalytic.

**Scheme 1:**
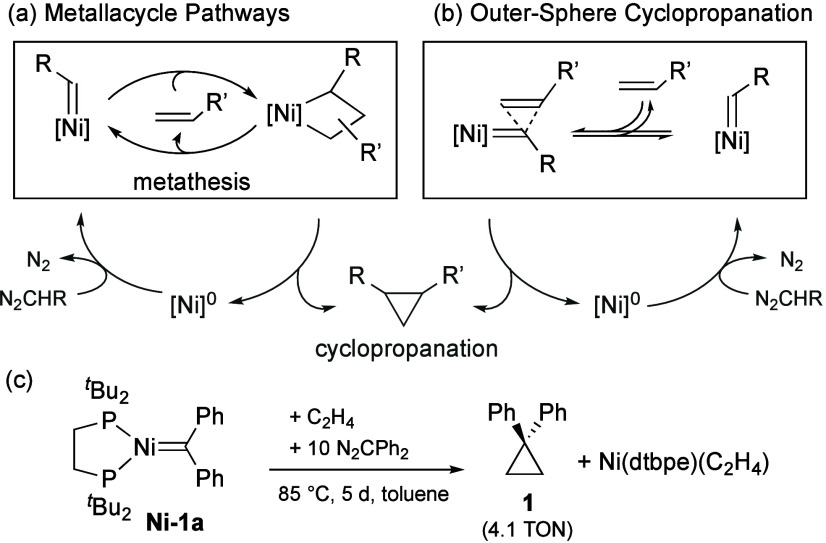
(a) Olefin Metathesis or Cyclopropanation via NiCB Intermediates. (b) Outer-Sphere Cyclopropanation. (c) Prior Work

Only cyclopropanation is well established in nickel chemistry to date.^12–14^ Tantalizing hints of metathesis activity are seen in early work, however,^15,16^ and the stoichiometric transformation of fluoroalkenes into metathesis products via Ni=CFR species (R = F, perfluoroalkyl) was recently established.^17–19^ Beyond fluorinated and pincer^7,20–22^ derivatives (with which catalytic metathesis has yet to be achieved), well-defined Ni=CRR’ complexes remain exceptionally rare. A single example was shown to react with olefins: Ni­(dtbpe)­(CPh_2_) (**Ni-1a**, Scheme 1c; dtbpe = bis­(di-*tert*-butyl-phosphinoethane), reported by Mindiola and Hillhouse more than two decades ago.^23^

Complex **Ni-1a**, the sole reactive member within a triad of Ni­(PP)­(=CRR’) complexes,^24^ generated cyclopropane **1** in near-stoichiometric yields on prolonged thermolysis under ethylene.^25^ Catalytic cyclopropanation was achieved with added Ph_2_CN_2_ (4 turnovers; Scheme 1c), but no NiCB could be detected. The Moret group recently reported retro-addition from a pincer-NiCB^26^ complex to form alkene and Ni-alkyl products: DFT calculations supported the intermediacy of a Ni=CAr_2_ species.^27^ Direct experimental evidence for olefin cycloaddition—a core requirement for both olefin metathesis and inner-sphere cyclopropanation—has been elusive outside the stoichiometric chemistry of [Ni]=CFR^F^ complexes. Here we report this missing evidence, demonstrating that unconstrained complexes of the type **Ni-1** engage in cycloaddition with olefins of widely differing electronic character to form NiCB species.

On the hypothesis that the phosphine bulk designed to stabilize **Ni-1a** retards its reaction with olefins, we sought sterically accessible variants. Diphosphines of widely varying stereoelectronic character (see **b**–**e**, Scheme 2a) were screened, with the objective of enabling cycloaddition, and uncovering any bias of the resulting NiCBs toward retro-addition or reductive elimination (i.e., metathesis or cyclopropanation, respectively). Accordingly, dcpe **b** was chosen for its steric adaptability vs dtbpe **a**; dibpp **c** for its flexibility^28^ and larger chelate ring size; diarylphosphine **d** for the greater rigidity of the ferrocenyl backbone and yet larger bite angle;^29^ and dpephos **e** for its capacity to adopt bi- or tridentate (fac, mer) geometries.^30,31^ The target complexes were synthesized via the Hillhouse protocol,^24,29^ by synthesis and UV irradiation of Ni­(PP)­(N_2_CPh_2_) **Ni-2** until elimination of N_2_ was complete (45 min–2.5 h). Diagnostic for **Ni-1** was a color change from orange to green (**b**, **c**) or yellow (**d**, **e**), emergence of a downfield ^31^P­{^1^H} NMR singlet in place of the doublets for **Ni-2**, and a ca. 100 ppm downfield shift in the ^13^C­{^1^H} NMR signal for the [Ni]=CPh_2_ carbon, to ca. 230 ppm.^20,23,24^ The observation of azine Ph_2_C=N–N=CPh_2_ in minor amounts points toward formation of the free carbene. **Ni-1** thus plausibly forms via a dissociative pathway involving decoordination and decomposition of Ph_2_CN_2_, and attack of :CPh_2_ at the Ni center.

**Scheme 2:**
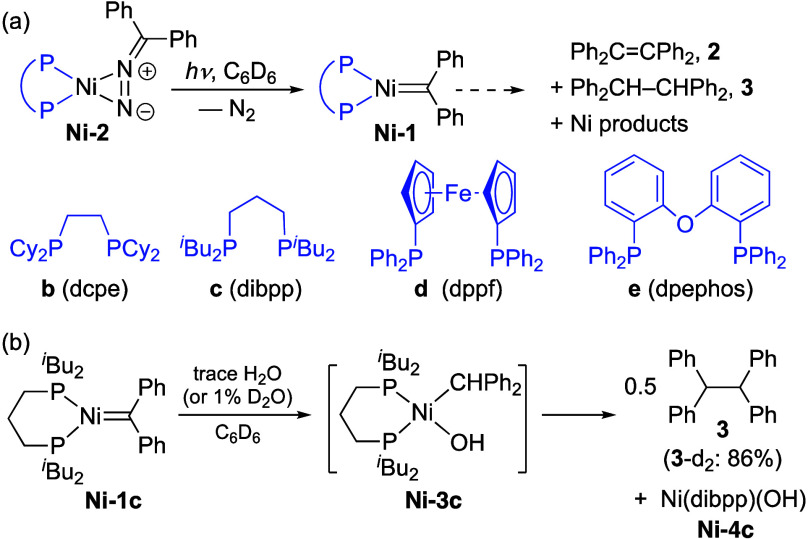
(a) Synthesis and Bimolecular Decomposition of Ni-1b–e. (b) Hydrolysis of Ni-1c, Showing Proposed Ni Species^a^

a For phosphine **a**, ^t^Bu_2_PCH_2_CH_2_P^*t*^Bu_2_, see Scheme 1.

The diphenylcarbene products **Ni-1b–e** are unstable in solution (benzene), an early indicator of their greater reactivity vs **Ni-1a**, which was found to withstand crystallization from concentrated solutions. Our efforts to isolate the most stable example, isobutylphosphine complex **Ni-1c**, were thwarted by decomposition to a black oil on concentrating. ^31^P­{^1^H} NMR analysis indicated near-complete disappearance of **Ni-1c**, and formation of unidentified Ni products (Figure S12). The remaining **Ni-1** complexes behaved similarly: that is, all can be generated cleanly in dilute solution (ca. 3 mM), but decompose readily at higher concentrations. Deliberately concentrating a 3.4 mM sample of **Ni-1c** for GC/MS analysis of the organic decomposition products revealed only Ph_2_C=CPh_2_ **2** and minor amounts of the corresponding alkane **3** (20% vs **2**; Figure S13), in addition to the benzophenone oxidation product resulting from exposure to air.

We attribute formation of **2** to bimolecular coupling, as reported for prior [M]=CPh_2_ complexes of Ni and Cu.^32,33^ Given the basicity established for related ligands^7^ (confirmed for **Ni-1c** by reaction with HCl),^29^ we suspected that alkane **3** may be an adventitious hydrolysis product, formed via protonation of the Ni=*C*Ph_2_ carbon by trace water, and ensuing coupling of the Ni-alkyl complex (Scheme 2b). Consistent with this hypothesis, deliberately adding D_2_O to **Ni-1c** in C_6_H_6_ caused an instant color change from green to brown, disappearance of the ^31^P­{^1^H} NMR signal for **Ni-1c**, and 86% formation of **3**-**d**_**2**_. EPR analysis was undertaken to detect the putative Ni­(I)–OH coproduct **Ni-4c**. A Ni­(I) species (g = 2.15) with two equivalent phosphines (a = 73 G) was detected at RT, but no additional hyperfine couplings could be resolved down to 90 K.

In seeking to drive cycloaddition reactions of **Ni-1b–e**, we employed two olefins of widely different electronic character, styrene and phenyl vinyl sulfone (PVS). Reaction with a 50-fold excess of styrene at room temperature resulted in complete consumption of the Ni complexes within 24 h. Cyclopropane **1** ^**S**^ formed in all cases (Table 1, entries 1–4), accompanied by variable proportions of hydrolysis product **3**.^34^ For **Ni-1c/d** (entries 2, 3), propene **4** ^S^ also formed. The latter is important as a marker for metallacyclobutane β-elimination: as such, it offers the first unequivocal evidence for NiCB intermediates, despite the absence of a detectable NiCB at −50 °C even for **Ni-1c** (Figure S24).

**Table 1:**
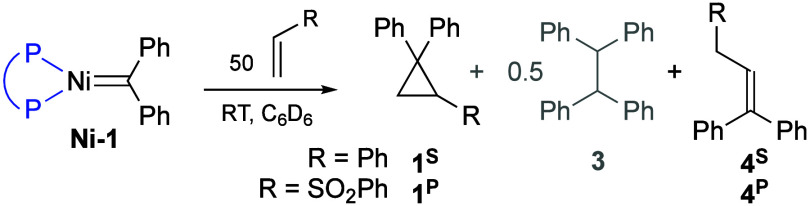
Reactivity of Ni-1b–e with Olefins (S = Styrene, P = PVS)^a^

	**Ni-1**: PP	olefin	conv.	%**1**	%**3**	%**4**
1	dcpe, **b**	styrene	100	43	**24**	0
2^b^	dibpp, **c**	styrene	100	9	**20**	70
3	dppf, **d**	styrene	100	42	**8**	25
4	dpephos, **e**	styrene	>88^c^	61	**13**	0
5	dibpp, **c**	PVS	100	0	**1**	84

a Conversions and yields measured by quantitative ^1^H NMR analysis at 24 h.

b To limit hydrolysis of long-lived **Ni-1c**, Ph_2_CN_2_ was dried over molecular sieves (15 min, −35 °C). For yields without drying, see Table S2.

c NMR signals for **Ni-1e** overlap with those for a byproduct.

The distinctive behavior of **Ni-1c/d** may reflect their reduced steric bulk (a function of the flexibility of the isobutyl substituents in **Ni-1c**,^28^ and the small phenyl groups in **Ni-1d**). In the cyclohexyl complex **Ni-1b**, in contrast, steric pressure is proposed to favor cyclopropanation by favoring reductive elimination of the NiCB. Bulk may also bias cycloaddition in favor of a β-substituted MCB, for which β-H elimination would plausibly be inhibited, leaving cyclopropanation as the only available reaction path. Lastly, formation of a κ^3^-POP species^30,31^ in the case of dpephos complex **Ni-1e** may block the open site required for β-H elimination. The absence of any clear trend in the reactivity from Table 1 may reflect counteracting steric and electronic properties. For example, a reduced bias toward reductive elimination is expected for the electron-rich alkylphosphine, but the greater bulk of these ligands would promote reductive elimination.

In exploring the impact of the electronic character of the olefin, we focused on **Ni-1c**, for which the evidence above indicates efficient formation of the NiCB. Unexpectedly, **Ni-1c** reacts dramatically faster with electron-deficient PVS than with styrene. Addition of even 1 equiv of PVS to **Ni-1c** causes an instant color change from green to bright red, with formation of a spectroscopically observable NiCB (**Ni-5c**, Scheme 3a).^29^ The ^1^H NMR spectrum shows an ABX spin system for the NiCB ring protons, within which the diastereotopic β-CH_2_ protons exhibit the expected correlations with H_α_ (^1^H–^1^H COSY), as well as with the adjacent *C*Ph_2_ carbon (^1^H–^13^C HMBC). NOESY-NMR analysis reveals a through-space interaction between the latter and H_α_. X-ray-quality crystals deposited from solution on repeating the reaction at larger scale, lyophilizing, and immediately adding cold diethyl ether. Crystallographic analysis (Scheme 3b) confirmed the successful interception of **Ni-5c**, the first example of an observable NiCB formed via [2 + 2] cycloaddition of an olefin to a conventional Ni-carbene. The complex exhibits a slightly distorted square-planar geometry at Ni (τ_4_ = 0.25),^35^ with bond metrics comparable to those for the pincer NiCB reported by Moret and co-workers.^27^

**Scheme 3:**
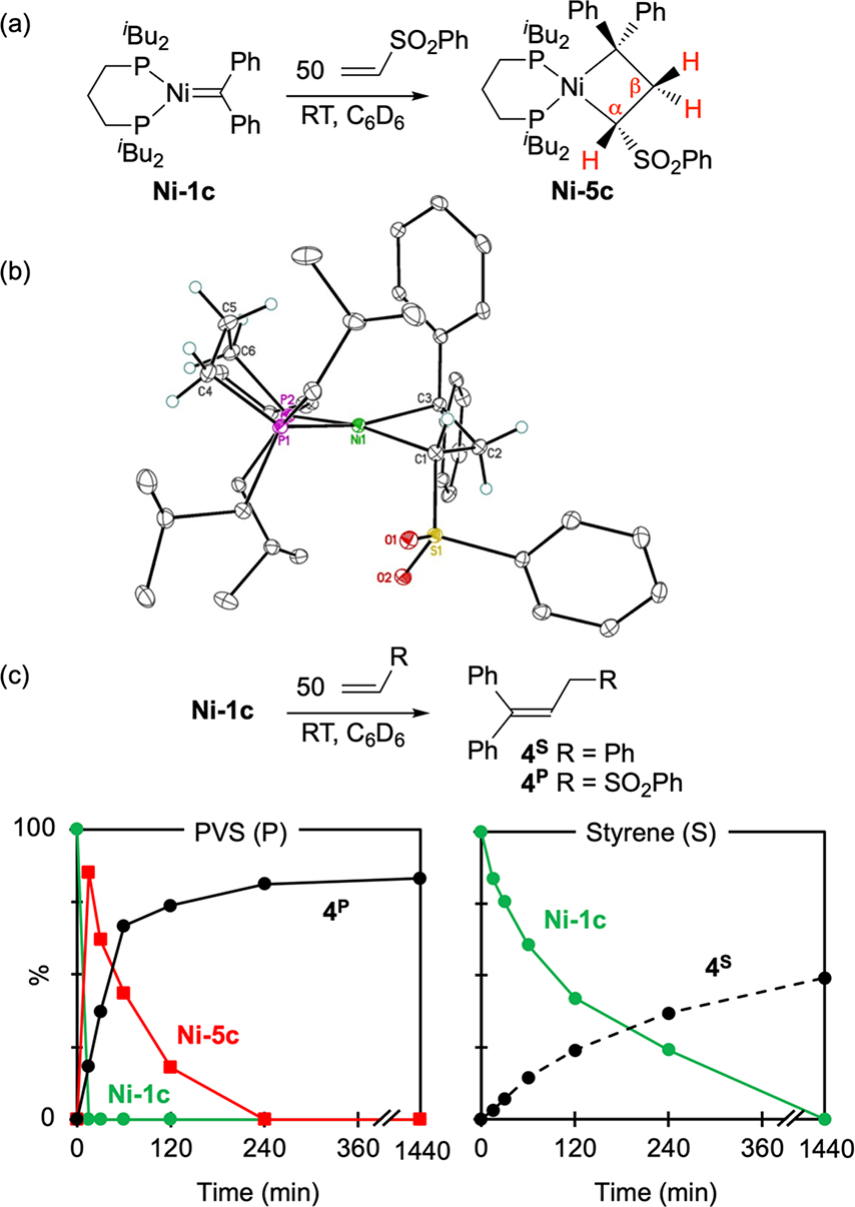
(a) Reaction of Ni-1c with PVS. (b) Crystal Structure of NiCB Cycloaddition Product Ni-5c. (c) Rate Profiles for Reactions of Ni-1c with Olefins

The rate profiles for reactions of **Ni-1c** with 50 equiv of PVS or styrene are shown in Scheme 3c. For PVS, immediate conversion to the NiCB **Ni-5c** is followed by slower β-H elimination to form propene **4** ^**P**^, with complete decomposition within 4 h (vs 24 h to **4** ^**S**^ for styrene). The high propene yield for PVS (85%) implies that NiCB formation is rapid relative to hydrolysis. Also of note is the much faster reaction with this electron-deficient olefin, suggesting either accelerated binding or faster [2 + 2] cycloaddition at the electron-rich Ni center. Addition of oxidizing agent [FeCp_2_]­[PF_6_] to **Ni-5c** did not induce metathesis to form CH_2_=CPh_2_ **5**.^36^ Instead, a small proportion of cyclopropanation was observed, accompanied by the β-elimination product **4** ^**P**^.

The computed energy barriers for the reaction of **Ni-1c** with styrene or PVS (Figure 1) are consistent with the experimental findings. The calculations reveal an essentially square-planar, singlet NiCB complex. For both substrates, the β-substituted NiCB is less favored (Table S20). β-H elimination is energetically feasible for both substrates (as is cyclopropanation for styrene). The η^1^-allyl structure is favored over its η^3^-allyl isomer (10.9 vs 14.1 kcal/mol; Table S20), presumably reflecting the electronic preference for the 16-electron d^8^ metal. Cycloreversion requires rearrangement to a tetrahedral triplet structure, resulting in a substantially higher barrier to metathesis (Figure 1, red line).

**Figure 1:**
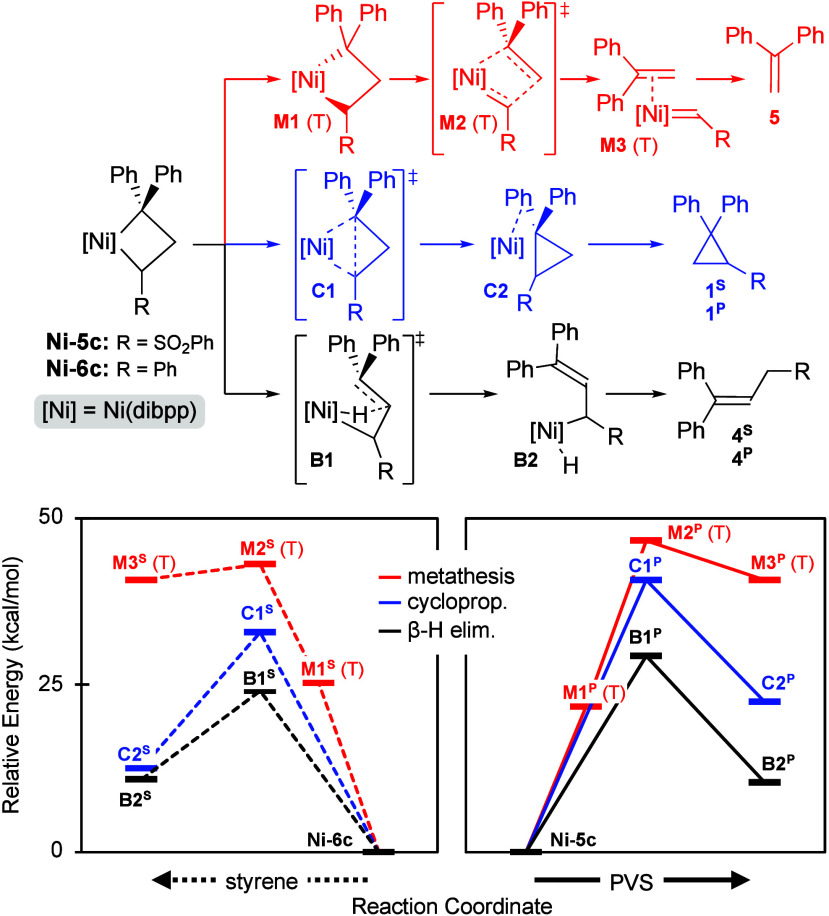
Reaction coordinate for **Ni-1c** + styrene or PVS (denoted with superscript S or P, respectively). Singlet states, except where labeled (T).

Natural bond orbital (NBO)^37^ and natural resonance theory (NRT)^38^ analyses of **Ni-1c** reveal surprisingly little covalent character in the nickel–carbene bond, with a Wiberg bond index of just 0.72, and predominantly ionic contributions to the natural bond order (BO = 1.24; 0.74 ionic, 0.50 covalent). Significantly greater covalency was reported for RuCl_2_(PCy_3_)­(=CH_2_) **GIm**, the 14-electron active species formed by the first-generation Grubbs catalyst.^39^ The Wiberg index for **GIm** is 1.64, more than double that of **Ni-1c**; the natural BO is also substantially higher (1.69) and, crucially, is almost entirely covalent (1.51 covalent, vs just 0.17 ionic). Accordingly, in the most representative Lewis structure for **GIm**, the σ and π NBOs of the M=C bond are weakly polarized toward carbon (55%) and metal (54%), respectively. For **Ni-1c**, a very different picture emerges for the most representative Lewis structure, which accounts for 98.3% of the electron density. Here the π-symmetry NBO is strongly polarized toward metal (73%, Figure S28a), whereas the σ component is best described as a carbon lone pair (Figure S28b) donating to a 4s-rich acceptor orbital on nickel (Figure S28c). Consistent with the near-absence of covalency, enforcing a M=C double bonded Lewis structure results in a σ-component NBO located almost solely on carbon, with only 13% metal character.

Nevertheless, NRT analysis reveals a 60% overall weight for resonance structures containing a M=C double bond, although structures describing the metal–carbene bond as exclusively donor–acceptor interactions also hold significant weight (>26%; Figure S27). The cumulative picture suggests a diphenylcarbene functioning primarily as a dative σ-donor to Ni, while accepting some degree of π-donation from the metal. **Ni-1c** can thus be viewed as a Ni(0) species with a donor–acceptor carbene, a subset of Fischer carbenes.^39^ Dissociation of the nickel–carbene bond to singlet fragments is consistently favored by >15 kcal/mol, whereas spin-triplet fragments are favored for the Grubbs catalysts.^39^ A recent computational analysis by the Moret group demonstrates that other [Ni]=CPh_2_ complexes fall into the same as **Ni-1c**.^7^ Important to note in this context is the metathesis activity of the iconic Casey carbene, W­(CO)_5_(=CPh_2_), arguably the best-known representative of this class.^5,6^

The foregoing demonstrates that Ni-carbene complexes participate in intermolecular [2 + 2] cycloaddition, an elementary step central to olefin metathesis. Indirect evidence for a NiCB intermediate in styrene metathesis is supplemented by direct X-ray and NMR observation of the metallacyclobutane in metathesis of phenyl vinyl sulfone. **Ni-5c** represents the first observable NiCB formed via [2 + 2] cycloaddition of a nonfluorinated [Ni]=CRR’ complex with alkenes. Computational analysis reveals a higher energy barrier to metathesis relative to reductive elimination of cyclopropane or β-H elimination, indicating that although cycloaddition is feasible for this system, cycloreversion is not. We are presently exploring factors that bias reactivity in favor of cycloreversion, an essential prerequisite to realizing the potential of nickel catalysts for olefin metathesis.

## Supplementary Material





## Notes

Experimental and computational details, NMR and MS spectra, GC traces (PDF)
Computational data set (XYZ)
