# Supplementary material for: Elementary Steps in Olefin Metathesis: Nickelacyclobutanes via Cycloaddition to Nickel Carbenes
Source: J Am Chem Soc. 2025 Jul 7;147(28):24225–9. doi: 10.1021/jacs.5c06505 (PMC12272553; doi:10.1021/jacs.5c06505)
Supplement: Supplementary file 1 [file ja5c06505_si_001.pdf]

Supporting Information

Elementary Steps in Olefin Metathesis: Nickelacyclobutanes via Cycloaddition to Nickel Carbenes

Samantha K. Cormier,[a] Marco Foscato,[b] Michael J. Ferguson,[c] Vidar Jensen,[b] R. Tom Baker,*[a] and Deryn E. Fogg*[a,b]

[a]Center for Catalysis Research & Innovation and Department of Chemistry and Biomolecular Sciences, University of Ottawa, Ottawa, ON K1N 6N5, Canada. [b]Department of Chemistry, University of Bergen, Allégaten 41, N-5007, Bergen, Norway. [c]X-ray Crystallography Laboratory. Department of Chemistry University of Alberta, 11227 Saskatchewan Dr., Edmonton, AB T6G 2G2, Canada.

*Corresponding authors: dfogg@uottawa.ca, deryn.fogg@uib.no, rbaker@uottawa.ca

Table of Contents

S1. Experimental **S2**
S1.1 General Procedures. S2
S1.2 Synthesis of Diazo Adducts Ni(PP)(N_2CPh_2), **Ni-2** S3
S1.3 In Situ Generation of Carbene Complexes Ni(PP)(CPh_2), **Ni-1** S5
S1.4 Synthesis of NiCB Complex **Ni-5c** S7
S1.5 Synthesis of Ni(PP)(Olefin) Adducts S7
S1.6 Reactivity of Carbene Complexes **Ni-1** S8
S1.7 Synthesis of Organic Compounds S11
S2. NMR Spectra and Characterization Data **S12**
S2.1 NMR Spectra of Diazo Adducts **Ni-2** S12
S2.2 In Situ Characterization of Carbene Complexes **Ni-1** S24
S2.3 In Situ Characterization of NiCB Complex **Ni-5c** S34
S2.4 Characterization of Ni(PP)(Olefin) Adducts S39
S2.5 Decomposition in Solution of Carbene Complexes **Ni-1** S45
S2.6 Mass Spectra S48
S2.7 Analysis of Reactions of **Ni-1** Complexes with Styrene S58
S3. X-ray Crystallographic Analysis **S60**
S3.1 X-ray Analysis of NiCB Complex **Ni-5c** S60
S3.2 X-ray Crystallographic Analysis of Ni(dcpe)(N_2CPh_2), **Ni-2b** S71
S4. Computational Details **S85**
S5. References **S91**

S1. Experimental

S1.1 General Procedures. All reactions were carried out in an N_2-filled glovebox unless otherwise noted. HPLC-grade THF, benzene, and hexanes were dried and degassed with a Glass Contour solvent purification system and stored under N_2 over 4 Å molecular sieves for at least 24 h prior to use, to attain water content of ≤4 ppm (confirmed by Karl-Fischer titration). Pentane and toluene were distilled over P_2O_5 and stored under N_2 over 4 Å molecular sieves for at least 24 h prior to use. Benzophenone tosylhydrazone and 3,3-diphenylallyl phenyl sulfone $\mathbf{4^P}$ were prepared by literature methods.[1,2] Ni(COD)(dppf)[3] and Ni(COD)(dpephos)[4] were synthesized by the reported procedures, but in THF. C_6D_6 (Cambridge Isotopes), D_2O (Sigma-Aldrich, 99.9 %) and styrene (Sigma-Aldrich, 99%) were freeze-pump-thaw degassed (4×) and stored under N_2 in the glovebox; C_6D_6 was stored over 4 Å sieves for 12 h before use. Bis(cyclooctadiene)nickel (Sigma-Aldrich or Strem Chemicals; no difference in performance was noted), 1,2-bis(dicyclohexylphosphino)ethane (dcpe, **b**, Strem, 98%), 1,3-bis(diisobutylphosphino)propane (dibpp, **c**, Cytec, now Solvay), 1,1'-bis(diphenylphosphino)ferrocene (dppf, **d**, Ambeed, 97%), bis(2-diphenylphosphinophenyl)ether (dpephos, **e**, Ambeed, 97%), phenyl vinyl sulfone (PVS, Sigma-Aldrich, 99%), palladium on carbon (10% wt loading, Sigma-Aldrich), 1,3,5-trimethoxybenzene (TMB, TCI, 98%), 1,1,2,2-tetraphenylethylene (Sigma-Aldrich, 98%), 1,2-diphenylethylene (Sigma-Aldrich, 97%), HCl solution (Sigma-Aldrich, 2.0 M in diethyl ether) and anhydrous diethyl ether (Sigma-Aldrich 99.7%), ferrocenium hexafluorophosphate (97%, Sigma-Aldrich) were used as received.

NMR spectra were recorded on Bruker Avance 300, Avance II 300, Avance II 400, Avance III 500, or Avance III 600 MHz NMR spectrometers at 25 °C. Chemical shifts are given in ppm, referenced to the residual proton signal of the deuterated solvent (^{1}H NMR), the carbon signals of the deuterated solvent ($^{13}C\{^1H\}$ NMR), or external 85% H_3PO_4 ($^{31}P\{^1H\}$ NMR).

GC-MS data were collected on an Agilent Technologies 5975B Inert XL EI/CI MSD instrument. MALDI mass spectra were collected on a Bruker UltrafleXtreme MALDI-TOF/TOF mass spectrometer in positive reflectron mode, calibrated using a Lipidomix calibration standard (mass range 155.0344–1046.5418 g/mol) with α-cyano-4-hydroxycinnamic acid (HCCA) as the matrix. Samples for analysis were prepared in the glovebox by evaporating a droplet of the complex in THF (without matrix) directly onto the plate. The plate was removed from inert atmosphere immediately prior to analysis. LIFDI mass spectra were measured by the Mass Spectrometry Facility at the University of British Columbia using a Jeol JMS-T100GCV AccuTOF GCv 4G instrument equipped with a LIFDI probe. Samples were prepared in degassed THF in a glovebox.

Photochemical experiments were conducted via irradiation with a DarkBeam® A300 UV 395 nm rechargeable flashlight. SCXRD data were collected on a Bruker Kappa ApexII diffractometer using graphite-monochromated Mo Kα (0.71073) at 100 K.

EPR data were collected using a Bruker EMX plus EPR spectrometer. The samples were prepared in toluene, and the spectrum was swept from 255 to 370 mT. The modulation amplitude was 0.1 mT, modulation frequency 100 kHz and microwave power 2 mW; 11200 points collected; sweep time 67.2 s, conversion time 6 ms, time constant 0.01 ms. Room-temperature measurements: microwave resonance frequency 9.389916 GHz; 16 scans collected. Fitting was performed using SpinFit. Measurements at 90 K: Microwave resonance frequency 9.39141 GHz; 216 scans.

Table S1. Bite angles and chelate ring sizes for diphosphines **b-e**.

diphosphine, PP	chelate size	bite angle (°)	reference complex for bite angle determination	ref
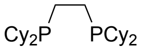 **b** dcpe	5	91	Ni(dcpe)(N_2CPh_2), **Ni-2b**	This work
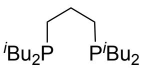 **c** dibpp	6	92	Ni(dibpp)(κ^2-$PhSO_2CHCH_2CP_2$), **Ni-5c**	This work
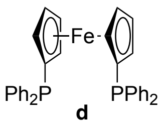 **d** dppf	ambiguous	105	Ni(dppf)(COD)	3
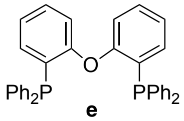 **e** dpephos	8	106	Ni(dpephos)(COD)	4

S1.2 Synthesis of Diazo Adducts Ni(PP)(N_2CPh_2), Ni-2

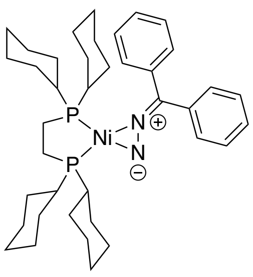

S1.2.1 Ni(dcpe)(N_2CPh_2), Ni-2b. To a stirred yellow solution of Ni(COD)$_2$ (102 mg, 0.37 mmol) in ca. 4 mL THF was added dcpe (155 mg, 0.37 mmol, 1.0 equiv). A slight deepening of colour was observed. Stirring was continued for 1 h. Addition of a purple solution of Ph_2CN_2 (78 mg, 0.40 mmol, 1.1 equiv) in 1 mL THF (dropwise, over ca. 2 min) caused a colour change to orange. The solution was stirred for 2 h, at which point $^{31}P\{^1H\}$ NMR analysis confirmed that reaction was complete. The reaction was filtered through Celite to remove any insoluble Ni decomposition products. The filtrate was concentrated to ca. 0.5 mL and ca. 3 mL hexanes was added to precipitate the bright orange product. The latter was filtered off, washed with hexanes (3 × 1 mL) and dried under vacuum. Isolated yield: 154 mg (63%). For multinuclear 1D and 2D NMR spectra showing signal assignments, see Figure S1.

^1H NMR (600 MHz, C_6D_6): δ 8.53 (d, $^3J_{HH}$ = 8.2 Hz, 2H, ArC*H*), 7.54 (d, $^3J_{HH}$ = 7.3 Hz, 2H, ArC*H*), 7.37 (t, $^3J_{HH}$ = 7.6 Hz, 2H, ArC*H*), 7.24 (t, $^3J_{HH}$ = 7.6 Hz, 2H, ArC*H*), 7.07 (t, $^3J_{HH}$ = 7.3 Hz, 1H, ArC*H*), 6.98 (t, $^3J_{HH}$ = 7.3 Hz, 1H, ArC*H*), 2.18-2.14 (m, 2H, dcpe), 1.93-1.88 (m, 2H, dcpe), 1.75-0.95 (m, 46H, dcpe), 0.79-0.70 (m, 2H, dcpe).

$^{13}C\{^1H\}$ NMR (151 MHz, C_6D_6): δ 143.1 (Ar*C*), 137.8 (Ar*C*), 137.7 (Ar*C*H), 131.9 (Ar*C*H), 128.5 (Ar*C*H), 128.4 (Ar*C*H), 125.1 (Ar*C*H), 124.4 (Ar*C*H), 121.7 (Ar*C*H), 101.3 (t, $^3J_{CP}$ = 7 Hz, (Ar$_2$*C*=N)), 34.6 (dd, J_{CP} = 18, 3 Hz, dcpe), 33.9 (dd, J_{CP} =18, 4 Hz, dcpe), 30.0 (d, J_{CP} = 7 Hz, dcpe), 29.6 (d, J_{CP} = 4 Hz, dcpe), 29.5 (dcpe), 29.1 (dcpe), 27.5 (dcpe), 27.5 (dcpe), 27.4 (dcpe), 27.4 (dcpe), 27.3 (dcpe), 26.5 (d, J_{CP} = 7 Hz, dcpe), 21.3 (dd, J_{CP} = 23, 15 Hz, dcpe), 20.5 (dd, J_{CP} = 23, 15 Hz, dcpe).

$^{31}P\{^1H\}$ NMR (202 MHz, C_6D_6): δ 68.5 (d, $^2J_{PP}$ = 55 Hz), 59.3 (d, $^2J_{PP}$ = 55 Hz).

MALDI-MS (THF, matrix-free): Calc'd for $C_{39}H_{59}N_2NiP_2$: *m/z* 675.35 (M^{+}+ H). Found: *m/z* 675.25. For mass spectrum showing experimental and predicted isotope patterns, see Figure S16.

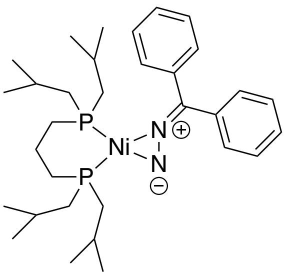

S1.2.2 Ni(dibpp)(N_2CPh_2), Ni-2c. As for **Ni-2b**, with workup by removal of solvent at 2.5 h. The resulting red oil was dissolved in 2 mL hexanes and filtered (Celite). The filtrate was evaporated, 1.5 mL pentane was added, and the sample was chilled to –35 °C. A red precipitate deposited over 2 h. The latter was filtered off, washed with cold pentane (3 × 0.5 mL), and dried under vacuum. Isolated yield: 127 mg (50%). For multinuclear 1D and 2D NMR spectra showing signal assignments, see Figure S2.
Experiment with reduced water content: As above, but storing the diphenyldiazomethane solution over 4 Å molecular sieves at –35 °C for 15 min prior to use. Yield: 56 mg (41%).
^{1}H NMR (600 MHz, C_6D_6): δ 8.39 (d, $^{3}J_{HH}$ = 7.6 Hz, 2H, ArC*H*), 7.58 (d, $^{3}J_{HH}$ = 7.5 Hz, 2H, ArC*H*), 7.35 (t, $^{3}J_{HH}$ = 7.5 Hz, 2H, ArC*H*), 7.21 (t, $^{3}J_{HH}$ = 7.5 Hz, 2H, ArC*H*), 7.05 (t, $^{3}J_{HH}$ = 7.5 Hz, 1H, ArC*H*), 6.97 (t, $^{3}J_{HH}$ = 7.5 Hz, 1H, ArC*H*), 2.22-2.14 (m, 2H, ^{i}BuC*H*), 1.73-1.67 (m, 2H, ^{i}BuCH_2), 1.65-1.57 (m, 2H, ^{i}BuC*H*), 1.42-1.31 (m, 4H, ^{i}BuCH_2 + dibpp CH_2), 1.17 (d, $^{3}J_{HH}$ = 6.8 Hz, 6H, ^{i}BuCH_3), 1.13 (d, $^{3}J_{HH}$ = 6.8 Hz, obscured, ^{i}BuCH_3), 1.11 (m, obscured, dibpp CH_2), 1.06 (d, $^{3}J_{HH}$ = 6.8 Hz, 6H, ^{i}BuCH_3), 1.01-0.97 (m, 2H, dibpp CH_2), 0.93-0.83 (m, obscured, ^{i}BuCH_2), 0.90 (d, $^{3}J_{HH}$ = 6.8 Hz, 6H, ^{i}BuCH_3).
^{13}C{^{1}H} NMR (151 MHz, C_6D_6): δ 142.7 (Ar*C*), 137.7 (d, J_{CP} = 6 Hz, Ar*C*), 131.9 (Ar*C*H), 128.6 (Ar*C*H), 128.3 (Ar*C*H), 125.2 (Ar*C*H), 124.4 (Ar*C*H), 121.7 (Ar*C*H), 98.8 (t, apparent, J_{CP} = 6 Hz, Ar_2*C*=N), 38.8 (dd, J_{CP} = 19, 3 Hz, ^{i}Bu*C*H_2), 38.5 (dd, J_{CP} = 19, 3 Hz, ^{i}Bu*C*H_2), 27.7 (dd, J_{CP} = 20, 3 Hz, dibpp *C*H_2), 26.5 (d, J_{CP} = 20 Hz, dibpp *C*H_2), 26.0 (dd, J_{CP} = 20, 3 Hz, ^{i}Bu*C*H), 25.7 (d, J_{CP} = 8 Hz, ^{i}Bu*C*H_3), 25.3 (d, J_{CP} = 7 Hz, ^{i}Bu*C*H_3), 45.7 (d, J_{CP} = 8 Hz, ^{i}Bu*C*H_3), 20.9 (dibpp *C*H_2).
^{31}P{^{1}H} NMR (202 MHz, C_6D_6): δ 12.8 (d, $^{2}J_{PP}$ = 23 Hz),–0.46 (d, $^{2}J_{PP}$ = 23 Hz)
LIFDI-MS (THF): Calc'd for $C_{32}H_{52}N_2NiP_2$ (M^{+}) *m/z* 584.30. Found: *m/z* 584.34. For spectrum showing experimental and predicted isotope patterns, see Figure S18.

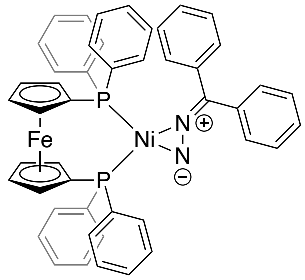

S1.2.3 Ni(dppf)(N_2CPh_2), Ni-2d. As for **Ni-2b** but using pre-formed phosphine complex Ni(COD)(dppf). Colour change: yellow to orange-brown. Isolated yield: 108 mg (67%). For multinuclear 1D and 2D NMR spectra showing signal assignments, see Figure S3.
^{1}H NMR (600 MHz, C_6D_6): δ 8.40 (d, $^{3}J_{HH}$ = 7.6 Hz, 2H, ArC*H*), 8.18 (t, $^{3}J_{HH}$ = 8.3, 4H, Ph), 7.66 (t, $^{3}J_{HH}$ = 8.3, 4H, Ph), 7.35 (br, 4H, ArC*H*), 7.07-6.95 (m, 15H, ArC*H* + Ph), 6.58 (t, $^{3}J_{HH}$ = 6.9 Hz, 1H, ArC*H*), 6.49 (t, $^{3}J_{HH}$ = 6.9 Hz, 2H, ArC*H*), 4.34 (s, 2H, Cp-CH_2), 3.91 (s, 2H, Cp-CH_2), 3.84 (s, 2H, Cp-CH_2), 3.71 (s, 2H, Cp-CH_2).
^{13}C{^{1}H} NMR (151 MHz, C_6D_6): δ 140.1, (d, J_{CP} = 7 Hz, Ar*C*), 137.4 (d, J_{CP} = 7 Hz, Ar*C*), 136.2 (d, J_{CP} = 38 Hz, Ph), 135.1 (d, J_{CP} = 12 Hz, Ph), 134.9 (d, J_{CP} = 12 Hz, Ph), 134.2 (d, J_{CP} = 34 Hz, Ph), 131.1 (Ar*C*H), 130.1 (d, J_{CP} = 7 Hz, Ph), 128.6 (Ar*C*H), 128.5 (Ar*C*H), 125.6 (Ar*C*H), 124.8 (Ar*C*H), 122.5 (Ar*C*H), 97.2 (m, Ar_2*C*=N), 82.5 (d, J_{CP} = 47 Hz, Cp-C), 77.6 (d, J_{CP} = 45 Hz, Cp-C), 74.7 (d, J_{CP} = 10 Hz, Cp-CH_2), 74.0 (d, J_{CP} = 9 Hz, Cp-CH_2), 72.6 (d, J_{CP} = 5 Hz, Cp-CH_2), 71.8 (d, J_{CP} = 5 Hz, Cp-CH_2).
^{31}P{^{1}H} NMR (202 MHz, C_6D_6): δ 30.2 (d, $^{2}J_{PP}$ = 27 Hz), 24.9 (d, $^{2}J_{PP}$ = 27 Hz).

LIFDI-MS (THF): Calc'd for $C_{47}H_{38}FeNiP_2$ (**Ni-1d**, $M^+–N_2$) *m/z*. 778.12. Found: *m/z* 778.15. No **Ni-2d** was detected. For mass spectra showing experimental and predicted isotope patterns, see Figure S20.

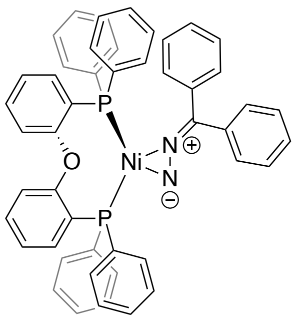

S1.2.4 Ni(dpephos)(N_2CPh_2), Ni-2e. As for **Ni-2b**, but using pre-formed phosphine complex Ni(COD)(dpephos). Colour change: yellow to orange-brown. Isolated yield: 275 mg (78%). For multinuclear 1D and 2D NMR spectra showing signal assignments, see Figure S4. Low solubility in C_6D_6 and instability in CD_2Cl_2 precluded full assignment.
^{1}H NMR (600 MHz, C_6D_6): δ 8.35 (s, br, 2H, ArC*H*), 7.52-7.36 (m, 12H, ArC*H* + Ph), 7.06-6.95 (m, 14H), 6.85 (m, 1H), 6.74 (m, 1H, dpephos), 6.68-6.53 (m, 4H), 6.48 (m, 1H), 6.26 (s, br, 1H, dpephos), 6.12 (m, 1H)
^{13}C{^{1}H} NMR (151 MHz, C_6D_6): δ 159.8 (m, dpephos), 159.4 (m, dpephos), 140.2, 137.5, 136.3, 134.9 (d, J_{CP} = 15 Hz), 134.5 (d, J_{CP} = 10 Hz), 132.6 (m), 131.5, 131.4, 130.7, 129.9, 129.5, 128.6, 128.5, 128.4, 125.4, 125.3 (Ar*C*H), 124.9, 124.6, 123.2, 122.2, 118.2, 96.2 (m, Ar_2*C*=N).
^{31}P{^{1}H} NMR (202 MHz, C_6D_6): δ 26.7 (d, $^2J_{PP}$ = 39 Hz), 23.6 (d, $^2J_{PP}$ = 39 Hz).
LIFDI-MS (THF): Calc'd for $C_{49}H_{38}NiOP_2$ (**Ni-1e**, M^+-N_2) *m/z*. 762.18. Found: *m/z* 762.25 No **Ni-2e** was visible in the spectrum. For the LIFDI mass spectrum showing experimental and predicted isotope patterns, see Figure S21.

S1.3 In Situ Generation of Carbene Complexes Ni(PP)(CPh_2), Ni-1

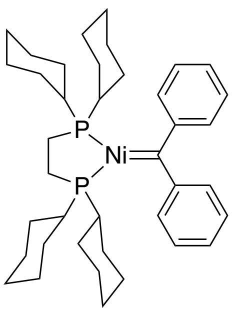

S1.3.1 Ni(dcpe)(CPh_2), Ni-1b. Orange **Ni-2b** (0.0034 mmol, 200 µL of a 10 mg/mL solution in C_6D_6) was dissolved in 800 µL C_6D_6 in a valved NMR tube. The tube was clamped vertically in front of a sheet of aluminum foil to enable reflection of UV light. The tube was irradiated at 395 nm for 1 h with periodic inversion of the tube. Conversion to green **Ni-1b** was complete after 1 h, as judged by ^{31}P{^{1}H} NMR analysis. For multinuclear 1D and 2D NMR spectra showing signal assignments, see Figure S5.
^{1}H NMR (600 MHz, C_6D_6): δ 8.10 (d, $^3J_{HH}$ = 7.5 Hz, 2H, ArC*H*), 7.36 (t, $^3J_{HH}$ = 7.3 Hz, 2H, ArC*H*), 7.21 (t, $^3J_{HH}$ = 7.3 Hz, 4H, ArC*H*), 2.01 (d, $^3J_{HH}$ = 12 Hz, 4H), 1.74-1.05 (m, 44H).
^{13}C{^{1}H} NMR (151 MHz, C_6D_6): δ 228.9 (t, J_{CP}= 51 Hz, Ar_2*C*), 159.4 (t, J_{CP} = 12 Hz, Ar*C*), 128.9 (Ar*C*), 125.0 (t, J_{CP} = 9 Hz, Ar*C*), 123.0 (Ar*C*), 35.5 (t, J_{CP} = 9 Hz, dcpe), 30.1 (dcpe), 29.6 (dcpe), 27.6 (t, J_{CP} = 5 Hz, dcpe), 27.4 (t, J_{CP} = 6 Hz, dcpe), 26.7 (dcpe), 21.0 (t, J_{CP} = 19 Hz, dcpe).
^{31}P{^{1}H} NMR (202 MHz, C_6D_6): δ 82.3.
MALDI-MS (THF, matrix-free): **Ni-2b** was analyzed by MALDI and found to convert to **Ni-1b** under ionization conditions. Calc'd for $C_{39}H_{57}NiP_2$: *m/z* 645.33 (M^+-H). Found: *m/z* 645.25. For mass spectrum showing experimental and predicted isotope patterns, see Figure S17.

S1.3.2 Ni(dibpp)(CPh$_2$), Ni-1c. As for **Ni-1b**. After 45 min irradiation, $^{31}P\{^1H\}$ NMR revealed full conversion to green **Ni-1c.** For multinuclear 1D and 2D NMR spectra showing signal assignments, see Figure S6.

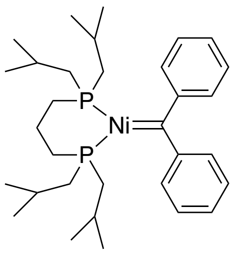

^1H NMR (500 MHz, C_6D_6): δ 8.02 (d, $^3J_{HH}$ = 8.0 Hz, 2H, ArC*H*), 7.38 (t, $^3J_{HH}$ = 7.8 Hz, 2H, ArC*H*), 7.17 (ArCH, obscured by solvent peak), 2.13-2.05 (m, 4H, ^{i}BuC*H*), 1.38-1.29 (m, 10H, dibpp C*H*$_2$), 1.21-1.16 (m, 4H, dibpp C*H*$_2$), 1.09 (d, $^3J_{HH}$ = 6.6 Hz, 12H, ^{i}BuC*H*$_3$), 0.95 (d, $^3J_{HH}$ = 6.6 Hz, 12H, ^{i}BuC*H*$_3$).
$^{13}C\{^1H\}$ NMR (151 MHz, C_6D_6): δ 231.0 (t, J_{CP} = 47 Hz, Ar$_2$*C*), 160.5 (t, J_{CP} = 10 Hz, Ar*C*), 128.7 (Ar*C*), 124.7 (t, J_{CP} = 8 Hz, Ar*C*), 123.1 (m, Ar*C*), 40.5 (dd, J_{CP} = 15, 4 Hz, dibpp *C*H$_2$), 28.1 (dd, J_{CP} = 16, 5 Hz, dibpp *C*H$_2$), 26.6 (d, J_{CP} = 5 Hz, ^{i}Bu*C*H), 25.6 (d, J_{CP} = 7 Hz, ^{i}Bu*C*H$_3$), 25.3 (d, J_{CP} = 7 Hz, ^{i}Bu*C*H$_3$), 20.1 (dibpp *C*H$_2$).
$^{31}P\{^1H\}$ NMR (202 MHz, C_6D_6): δ 18.7.
MALDI-MS (THF): Calc'd for (M^+ + H) 557.2970 *m/z*. Found: 557.297 *m/z*. For mass spectrum showing experimental and predicted isotope patterns, see Figure S19.

S1.3.3 Ni(dppf)(CPh$_2$), Ni-1d. As for **Ni-1b**. After 1h 40 min, $^{31}P\{^1H\}$ NMR revealed full conversion to yellow **Ni-1d**. For multinuclear 1D and 2D NMR spectra showing signal assignments, see Figure S7.

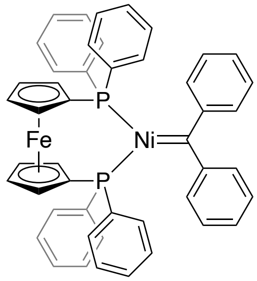

^1H NMR (600 MHz, C_6D_6): δ 8.05-8.00 (m, 11H ArC*H*), 7.38 (t, $^3J_{HH}$ = 7.0 Hz, 2H, ArC*H*), 7.08 (t, $^3J_{HH}$ = 7.6 Hz, 4H, ArC*H*), 7.02-6.96 (m, 12H ArC*H*), 4.25 (s, 4H, CpC*H*), 3.84 (s, 4H, CpC*H*).
$^{13}C\{^1H\}$ NMR (151 MHz, C_6D_6): δ 233.2 (detected HMBC, Ar$_2$*C*), 159.9 (detected HMBC Ar*C*), 136.9 (d, J_{CP} = 35 Hz, Ar*C*), 134.9-134.8 (m, Ar*C*), 129.7 (Ar*C*), 129.2 (Ar*C*), 125.4 (t, J_{CP} = 7 Hz, Ar*C*), 124.1 (Ar*C*), 82.7 (detected HMBC, Cp*C*) 74.4-74.3 (m, Cp*C*H), 71.3 (Cp*C*H).
$^{31}P\{^1H\}$ NMR (202 MHz, C_6D_6): δ 42.6.
LIFDI-MS (THF): **Ni-2d** was analyzed by LIFDI and found to convert to **Ni-1d** under ionization conditions. Calc'd for $C_{47}H_{38}FeNiP_2$ (M^+) *m/z* 778.12. Found: *m/z* 778.15. For mass spectrum showing experimental and predicted isotope patterns, see Figure S20.

S1.3.4 Ni(dpephos)(CPh$_2$), Ni-1e. As for **Ni-1b**. After 1h 40 min, $^{31}P\{^1H\}$ NMR revealed yellow **Ni-1e** as the major product (7:3 **Ni-2e).**

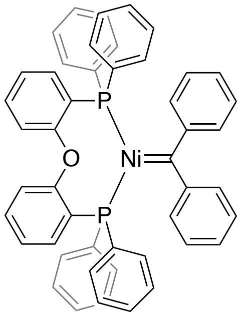

^1H NMR (600 MHz, C_6D_6): δ 8.07 (d, $^3J_{HH}$ = 7.4 Hz, 4H, ArC*H*). For multinuclear 1D and 2D NMR spectra showing signal assignments, see Figure S8. The instability of **Ni-1e** precluded full assignment of ^1H NMR signals.
$^{13}C\{^1H\}$ NMR (151 MHz, C_6D_6): δ 235.6 (detected HMBC, Ar$_2$*C*), 166.4 (detected HMBC), 165.3 (detected HMBC), 159.7 (detected HMBC), 143.9 (detected HMBC), 139.2 (detected HMBC), 137.0 (detected HMBC), 135.3 (detected HMBC), 133.8, 133.3, 130.3, 129.3, 125.3, 124.2, 123.6, 121.0, 118.3. *Lower sensitivity of $^{13}C\{^1H\}$ NMR allows for discrimination of peaks for **Ni-1e**. Peaks detected by HMBC were determined via overlay of HMBC spectra of **Ni-2e** and **Ni-1e**.
$^{31}P\{^1H\}$ NMR (202 MHz, C_6D_6): δ 40.7.
LIFDI-MS (THF): **Ni-2e** was analyzed by LIFDI and found to convert to **Ni-1e** under ionization conditions. Calc'd for $C_{49}H_{38}NiOP_2$ (M^+) *m/z* 762.18. Found: *m/z* 762.25. For mass spectrum showing experimental and predicted isotope patterns, see Figure S21.

S1.3.5 Attempted Preparative Synthesis of Ni(dibpp)(CPh_2), Ni-1c. An orange solution of **Ni-2c** (51 mg, 0.087 mmol) in 25 mL C_6H_6 in a 100 mL Kontes flask was irradiated as above, with a compressed-air flow directed at the bottom of the flask for cooling during the 16-h irradiation period. The flask was then returned to the glovebox, and an aliquot of the green solution was taken which confirmed complete conversion by $^{31}P\{^1H\}$ NMR. The solution was transferred to a 250 mL round-bottom flask, frozen (–35 °C freezer), and lyophilized for 5 h. The resulting green-black oil was diluted with 1 mL pentane and filtered through a fine frit to remove a small proportion of greasy black oil. The filtrate was chilled in the freezer overnight. No solid material was isolated. NMR analysis of the filtrate shows signals for **Ni-1c**, accompanied by extensive decomposition (Figure S12).
Generation of a 2 mg sample of **Ni-1c** in a valved NMR tube as in Section S1.3.2, followed by removal of solvent yielded a greenish-brown oil which was analyzed by GC/MS. The products consisted of benzophenone, alkene **2** and a small proportion of alkane **3** (Figure S13).

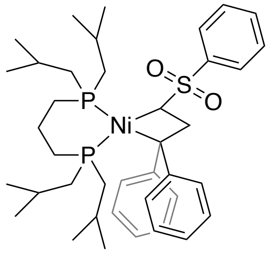

S1.4 Synthesis of NiCB Complex Ni-5c. In an NMR-scale experiment, a solution of **Ni-1c** in C_6D_6 generated as in S1.3.2 in a valved NMR tube was injected with PVS (29 μL, 0.0017 mmol, 1.0 equiv, 10 mg/mL solution in C_6D_6). The green solution immediately turned red. The tube was inverted to mix and immediately analyzed (NMR). For assignments, see Figure S9. Fresh samples were generated for each NMR experiment.
Larger scale: As above, in a round-bottom flask using **Ni-1c** (23 mg, 0.041 mmol, 1.0 equiv) in ca. 12 mL C_6H_6 and PVS (6.89 mg, 0.041 mmol, 1.0 equiv, 10 mg/mL solution in C_6H_6). After addition of PVS the solvent was lyophilized to dryness. Addition of Et_2O (ca. 1 mL) and swirling caused X-ray quality crystals to deposit.
^1H NMR (600 MHz, C_6D_6): δ 8.09 (d, $^3J_{HH}$ = 7.8 Hz, 2H, ArC*H*), 8.02 (d, $^3J_{HH}$ = 7.3 Hz, 2H, ArC*H*), 7.31 (d, $^3J_{HH}$ = 8.1 Hz, 2H, ArC*H*), 7.20 (t, $^3J_{HH}$ = 7.8 Hz, 2H, ArC*H*), 7.09 (t, $^3J_{HH}$ = 7.6 Hz, 2H, ArC*H*), 7.03 (m, 3H, ArC*H*), 6.97 (m, 2H, ArC*H*), 3.87 (dd, $^3J_{HH}$ = 9.8 14.2, Hz, 1H, β-MCB), 3.71 (dt, $^3J_{HH}$ = 8.6, 14.2, Hz, 1H, β-MCB), 3.33 (m, 1H, ^{i}Bu CH_2), 2.97 (dt, $^3J_{HH}$ = 5.0, 15.5, Hz, 1H), 2.38 (ddd, $^3J_{HH}$ = 1.5, 6.7, 14.2 Hz, 1H), 1.86 (m, 1H, ^{i}Bu CH_2), 1.75 (m, 1H, ^{i}Bu CH_2), 1.62 (d, $^3J_{HH}$ = 6.6 Hz, 3H, ^{i}Bu CH_3), 1.44 (m, 2H, ^{i}Bu CH_2, α-MCB), 1.37 (d, $^3J_{HH}$ = 6.6 Hz, 3H, ^{i}Bu CH_3), 1.11 (d, $^3J_{HH}$ = 7.3 Hz, 3H, ^{i}Bu CH_3), 1.06 (d, $^3J_{HH}$ = 6.7 Hz, 3H, ^{i}Bu CH_3), 0.84 (d, $^3J_{HH}$ = 6.7 Hz, 3H, ^{i}Bu CH_3), 0.81 (d, $^3J_{HH}$ = 6.6 Hz, 3H, ^{i}Bu CH_3), 0.67 (d, $^3J_{HH}$ = 6.7 Hz, 3H, ^{i}Bu CH_3), 0.66 (d, $^3J_{HH}$ = 6.7 Hz, 3H, ^{i}Bu CH_3).
$^{13}C\{^1H\}$ NMR (151 MHz, C_6D_6): Key signals: δ 18.6 (β-MCB), 21.0 (α-MCB) 140.9 (γ-MCB).
$^{31}P\{^1H\}$ NMR (202 MHz, C_6D_6): 7.56 (d, $^2J_{PP}$ = 20 Hz), –6.08 (d, $^2J_{PP}$ = 20 Hz).

S1.5 Synthesis of Ni(PP)(Olefin) Adducts

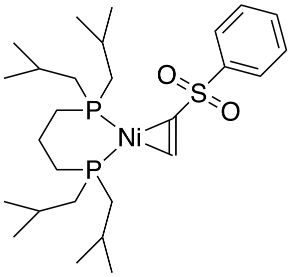

S1.5.1 Ni(dibpp)(PVS) Ni-7c. A solution of Ni(COD)$_2$ (21 mg, 0.076 mmol), dibpp (22 mg, 0.066 mmol, 0.87 equiv) in 1 mL THF was stirred for 5 min, and PVS was then added (12 mg, 0.071 mmol, 0.93 equiv). Sub-stoichiometric amounts of PVS and dibpp were used to limit the need for purification of oily **Ni-7c**. After a further 1 h of stirring, the yellow solution was filtered through Celite to remove decomposed Ni(COD)$_2$, and the filtrate was evaporated to leave a bright yellow oil.

The oil did not yield solid material on adding pentane, or further drying. For multinuclear 1D and 2D NMR spectra showing signal assignments, see Figure S10.

^{1}H NMR (600 MHz, C_6D_6): δ 8.22 (d, $^3J_{HH}$ = 8.1 Hz, 2H, Ar*CH*), 7.94 (d, $^3J_{HH}$ = 7.4 Hz, 2H, Ar*CH*), 7.00 (d, $^3J_{HH}$ = 7.1 Hz, 1H, Ar*CH*), 3.44 (m, 1H, C*H*=CH$_2$), 2.47 (m, 1H, dibpp), 2.23 (m, 1H, dibpp), 2.12 (m, 2H, C*H*=CH$_2$, dibpp), 2.00 (m, 2H, dibpp), 1.78 (m, 1H, dibpp), 2.68 (m, 2H, C*H*=CH$_2$, dibpp), 1.55 (m, 2H, dibpp), 1.47-1.33 (m, 3H, dibpp), 1.33-1.07 (m, 21H, dibpp), 0.99 (d, $^3J_{HH}$ = 6.4 Hz, 3H, dibpp), 0.93 (d, $^3J_{HH}$ = 6.9 Hz, 3H, dibpp), 0.86 (d, $^3J_{HH}$ = 6.9 Hz, 3H, dibpp).

^{13}C{^{1}H} NMR (151 MHz, C_6D_6): δ 148.1 (Ar*C*), 130.5 (Ar*C*H), 126.9 (Ar*C*H), 61.3 (dd, J_{CP} = 22, 4 Hz, *C*H=CH$_2$), 41.4 (d, J_{CP} = 15 Hz, dibpp), 41.3 (m, dibpp), 41.1 (m, dibpp), 40.5 (dd, J_{CP} = 19, 4 Hz, dibpp), 40.0 (d, J_{CP} = 15 Hz, dibpp), 39.0 (d, J_{CP} = 19 Hz, dibpp), 29.0 (dd, J_{CP} = 24, 3 Hz, *C*H=CH$_2$), 27.3 (dd, J_{CP} = 20, 4 Hz, dibpp), 26.4 (dd, J_{CP} = 24, 4 Hz, dibpp), 26.0 (dibpp), 25.9 (dibpp), 25.9-25.8 (m, dibpp), 25.7 (dibpp), 25.6-25.5 (m, dibpp), 25.4-25.3 (m, dibpp), 25.0 (d, J_{CP} = 7 Hz, dibpp), 24.6 (t, J_{CP} = 10 Hz, dibpp), 24.2 (d, J_{CP} = 4 Hz, dibpp), 21.5 (t, J_{CP} = 4 Hz, dibpp).

^{31}P{^{1}H} NMR (202 MHz, C_6D_6): δ 7.74 (d, $^2J_{PP}$ = 14 Hz), 3.45 (d, $^2J_{PP}$ = 14 Hz).

S1.5.2 Ni(dibpp)(styrene) Ni-8c. As for **Ni-7c**, but with addition of styrene (10 mg, 0.096 mmol, 1.26 equiv). The dark yellow oil did not yield solid material upon addition of pentane or further drying. For multinuclear 1D and 2D NMR spectra showing signal assignments, see Figure S11.

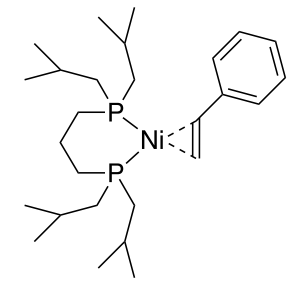

^{1}H NMR (600 MHz, C_6D_6): δ 7.27 (d, $^3J_{HH}$ = 8.1 Hz, 2H, Ar*CH*), 7.15 (obscured by solvent, Ar*CH*), 6.93 (d, $^3J_{HH}$ = 7.1 Hz, 1H, Ar*CH*), 3.95 (m, 1H, C*H*=CH$_2$), 2.48 (m, 1H, CH=C*H*$_2$), 2.31 (m, 1H, CH=C*H*$_2$), 1.98 (m, 2H, dibpp), 1.88 (m, 1H, dibpp), 1.56-1.42 (m, 4H, dibpp), 1.39-1.34 (m, 2H, dibpp), 1.33-1.23 (m, 4H, dibpp), 1.19-1.12 (m, 9H, dibpp), 1.11-0.99 (m, 11H, dibpp), 0.97 (d, $^3J_{HH}$ = 6.6 Hz, 3H, dibpp), 0.92-0.87 (m, 1H, dibpp), 0.77 (d, $^3J_{HH}$ = 6.5 Hz, 3H, dibpp), 0.67 (d, $^3J_{HH}$ = 6.7 Hz, 3H, dibpp).

^{13}C{^{1}H} NMR (151 MHz, C_6D_6): δ 150.1 (d, J_{CP} = 5 Hz, Ar*C*), 128.63 (detected, Ar*C*H), 124.7 (d, J_{CP} = 4 Hz, Ar*C*H), 121.1 (Ar*C*H), 53.4 (d, J_{CP} = 17 Hz, *C*H=CH$_2$), 41.8 (d, J_{CP} = 7 Hz, dibpp), 41.7 (d, J_{CP} = 6 Hz, dibpp), 41.7 (d, J_{CP} = 5 Hz, dibpp), 41.5 (d, J_{CP} = 4 Hz, dibpp), 40.1 (dd, J_{CP} = 15, 5 Hz, dibpp), 38.8 (dd, J_{CP} = 16, 4 Hz, dibpp), 32.8 (dd, J_{CP} = 19, 4 Hz, CH=*C*H$_2$), 28.9 (dd, J_{CP} = 16, 4 Hz, dibpp), 28.2 (dd, J_{CP} = 16, 4 Hz, dibpp), 26.7 (d, J_{CP} = 5 Hz, dibpp), 26.6 (d, J_{CP} = 5 Hz, dibpp), 26.2 (d, J_{CP} = 4 Hz, dibpp), 25.8 (d, J_{CP} = 9 Hz, dibpp), 25.7 (m, dibpp), 25.6 (d, J_{CP} = 6 Hz, dibpp), 25.4 (d, J_{CP} = 7 Hz, dibpp), 25.2 (m, dibpp), 25.1 (dibpp), 25.1 (dibpp) 21.5 (m, dibpp).

^{31}P{^{1}H} NMR (202 MHz, C_6D_6): δ 7.31 (d, $^2J_{PP}$ = 37 Hz), 0.78 (d, $^2J_{PP}$ = 37 Hz).

S1.6 Reactivity of Carbene Complexes Ni-1

S1.6.1 Reaction of Ni-1b-e with styrene. Solutions of **Ni-1** complexes in C_6D_6 were generated in valved NMR tubes as above (3.4 mM, 0.0034 mmol). Once full conversion was achieved, ca. 0.5 mg trimethoxybenzene (TMB) was added, a ^{1}H NMR spectrum was recorded to establish the initial ratio of TMB to **Ni-1**, and the sample was returned to the glovebox and transferred to a vial. Styrene (19 μL, 0.17 mmol, 50 equiv) was injected, and the reaction was stirred for 24 h. The conversion of **Ni-1** and yield of product were assessed by ^{1}H NMR analysis. Key signals for the

cyclopropanation ($\mathbf{1^S}$) and β-H elimination ($\mathbf{4^S}$) products appear at 2.75 and 3.40 ppm, respectively. The identity of $\mathbf{1^S}$ and $\mathbf{4^S}$ was validated by comparison of the signals in $CDCl_3$ to literature values.[5,6] Chemical shifts in C_6D_6 are provided below. GC/MS analysis (Figure S23) confirmed the absence of metathesis products, by comparison to commercial samples as references.

Table S2. Yields of organic products formed on reaction of **Ni-1c** with styrene.

reaction method	conv (%)	product distribution (%)		
		$\mathbf{1^S}$	**3**	$\mathbf{4^S}$
standard	100	6	52	30
additional drying	100	9	20	70

Characterization of organic products:
1,1,2-Triphenylpropane, $\mathbf{1^S}$. ^{1}H NMR (600 MHz, C_6D_6): δ 7.22 (d, $^3J_{HH}$ = 8.1 Hz, 2H), 7.11 (t, $^3J_{HH}$ = 7.6 Hz, 2H), 7.06 (d, $^3J_{HH}$ = 7.3 Hz, 2H), 7.02 (t, $^3J_{HH}$ = 7.4 Hz, 1H), 6.98-6.87 (m, 6H), 6.78 (d, $^3J_{HH}$ = 7.3 Hz, 2H), 2.75 (dd, $^3J_{HH}$ = 9.0, 6.5 Hz, 1H), 1.82 (t, $^3J_{HH}$ = 6.0 Hz, 1H), 1.57 (dd, $^3J_{HH}$ = 9.0, 5.4 Hz, 1H).
1,1,3-Triphenyl-1-propene, $\mathbf{4^S}$. ^{1}H NMR (300 MHz, C_6D_6): δ 7.27-7.06 (m, obscured by residual solvent peak), 6.23 (t, $^3J_{HH}$ = 7.5 Hz, 1H, 3.40 (d, $^3J_{HH}$ = 7.5 Hz, 2H).

S1.6.2 Reaction of Ni-1c with PVS. As above, using PVS (29 mg, 0.17 mmol, 50 equiv); mixing effected by affixing the NMR tube to a rotary stirrer. The diagnostic ^{1}H NMR signal for the β-H elimination product $\mathbf{4^P}$ appears at 3.64 ppm. Chemical shifts in C_6D_6 are provided below for convenience. ^{1}H NMR (600 MHz, C_6D_6): δ 7.66 (d, $^3J_{HH}$ = 7.7 Hz, 2H), 7.10 (m, 2H), 7.03-6.93 (m, 4H), 6.95 (t, $^3J_{HH}$ = 7.6 Hz, 2H), 6.86 (t, $^2J_{HH}$ = 7.3 Hz, 1H), 6.76 (t, $^3J_{HH}$ = 7.7 Hz, 2H), 6.59 (d, $^3J_{HH}$ = 7.3 Hz, 2H), 6.16 (t, $^3J_{HH}$ = 7.9 Hz, 1H), 3.64 (d, $^3J_{HH}$ = 8.0 Hz, 2H).

S1.6.3 Rate experiments for reactions of Ni-1c with styrene and PVS. As in S1.6.2, using **Ni-1c** (1 mg, 3.4 mM, 0.0017 mmol) and PVS or styrene (14 mg or 8.9 mg respectively; 0.085 mmol). NMR analysis was undertaken at 0, 15, 30 60, 120, 240 and 1440 min.

Table S3. Conversions and yields for the NMR-scale reaction of **Ni-1c** with PVS

	conv (%)	product yields (%)	
time (min)	**Ni-1c**	**Ni-5c**	$\mathbf{4^P}$
0	100	0	0
15	0	85	18
30	0	62	37
60	0	44	67
120	0	18	74
240	0	0	81
1440	0	0	83

Table S4. Conversions and yields for the NMR-scale reaction of **Ni-1c** with styrene. No Ni intermediate detected.

time (min)	conv (%) **Ni-1c**	yield (%) $\mathbf{4^S}$
0	100	0
15	84	3
30	76	7
60	61	14
120	42	24
240	24	37
1440	0	49

S1.6.4 Reactivity of Ni-1c with D_2O. A solution of **Ni-1c** was generated as above in a valved NMR tube (0.0017 mol in 500 µL C_6H_6). Once full conversion was confirmed ($^{31}P\{^1H\}$ NMR), 5 µL D_2O was added in the glovebox, and the tube was sealed and shaken. An immediate colour change from green to brown was observed. All **Ni-1c** was consumed within ca. 5 min of addition of D_2O. Over 2 h, the solution turned orange. The contents of the tube were transferred to a round-bottom-flask, and the solvent was removed on a rotary evaporator in air. The sample was dissolved in benzene, diluted with CH_2Cl_2 and analyzed by GC/MS (Figure S22). The extent of deuteration of tetraphenylethane **3** was assessed using the open-source software DGet!:[7] calculated 86% deuteration.
For EPR analysis: sample generated as above, but in toluene, with addition of H_2O instead of D_2O. The sample was analyzed immediately after adding H_2O. For spectra, see Figure S15.

S1.6.5 Reactivity of Ni-1c with HCl. Ni-1c was generated as above in a valved NMR tube (0.0017 mmol in 500 µL C_6D_6). After confirmation of full conversion by ^1H NMR analysis, HCl (5 µL; 2.0 M in Et_2O, 0.01 mmol, 5.9 equiv) was added in the glovebox, and the tube sealed and shaken. An immediate colour change from green to pale blue was observed. ^1H NMR analysis at 5 min revealed only paramagnetic products. The sample was diluted with CH_2Cl_2 and filtered through a short silica plug. GC/MS analysis of the filtrate revealed tetraphenylethane **3** as the major product (Figure S14).

S1.6.6 Reactivity of Ni-5c with [FeCp$_2$][PF$_6$]. Ni-1c (1 mg, 0.0017 mmol) generated as above in a valved NMR tube. After confirmation of full conversion by ^1H NMR, PVS was added (0.29 mg, 0.0017 mmol, 26 µL 11 mg/mL solution in C_6D_6, 1.0 equiv) and the sample was immediately lyophilized to dryness in a vial. To the vial was then added 0.5 mL THF and [FeCp$_2$][PF$_6$] (0.62 mg, 0.0019 mmol, 62 µL 10 mg/mL solution in THF, 1.1 equiv). The red solution was allowed to stir for 3 h at which point the solution was analyzed by GC/MS revealing majority $\mathbf{4^P}$. No diphenyl ethylene was detected.

S1.7 Synthesis of Organic Compounds

S1.7.1 Synthesis of diphenyldiazomethane (6). *Caution! Aryldiazomethane reagents are highly reactive, toxic, and often explosive.* A suspension of benzophenone hydrazone (2.03 g, 5.79 mmol) in 30 mL triethylene glycol in a 100 mL round-bottom flask equipped with a stir bar was heated to 70 °C, and KOH (656 mg, 11.7 mmol, 2.02 equiv) was added. The reaction turned pink within 5 min, and bright red after stirring for 30 min. Distilled water (30 mL) was added to the flask, causing a precipitate to form. The flask was transferred to an ice-bath to cool to RT, and the product was extracted into pentane (7 × 15 mL). The organic phase was washed with brine, then water (15 mL each), dried over $MgSO_4$, and stripped to dryness. Yield: 415 mg (37%). The pink oil was used without purification.

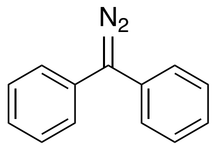

S1.7.2 Synthesis of 1,1,2,2-tetraphenylethane (3). A high-pressure reaction vessel was charged with tetraphenylethane **2** (51 mg, 0.153 mmol, 1 equiv) (16 mg (Pd/C (10 wt%; 0.015 mmol, 0.1 equiv) and 5 mL MeOH. The reactor was sealed, pressurized to 3 bar H_2, and heated to 50 °C for 2 h. Following depressurization, the suspension was filtered through a pad of Celite, and the solvent evaporated. Yield: 40 mg (78% yield). ^{1}H NMR chemical shifts in $CDCl_3$ agree with literature values;[8] values in C_6D_6 are provided here for convenience.

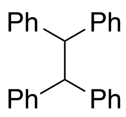

^{1}H NMR (300 MHz, C_6D_6): δ 7.11-7.08 (m, 7H), 7.00-6.83 (m, 13H), 4.69 (s, 2H).

S2. NMR Spectra and Characterization Data

S2.1 NMR Spectra of Diazo Adducts Ni-2

S2.1.1 Ni(dcpe)(N_2CPh_2), Ni-2b.

(a)

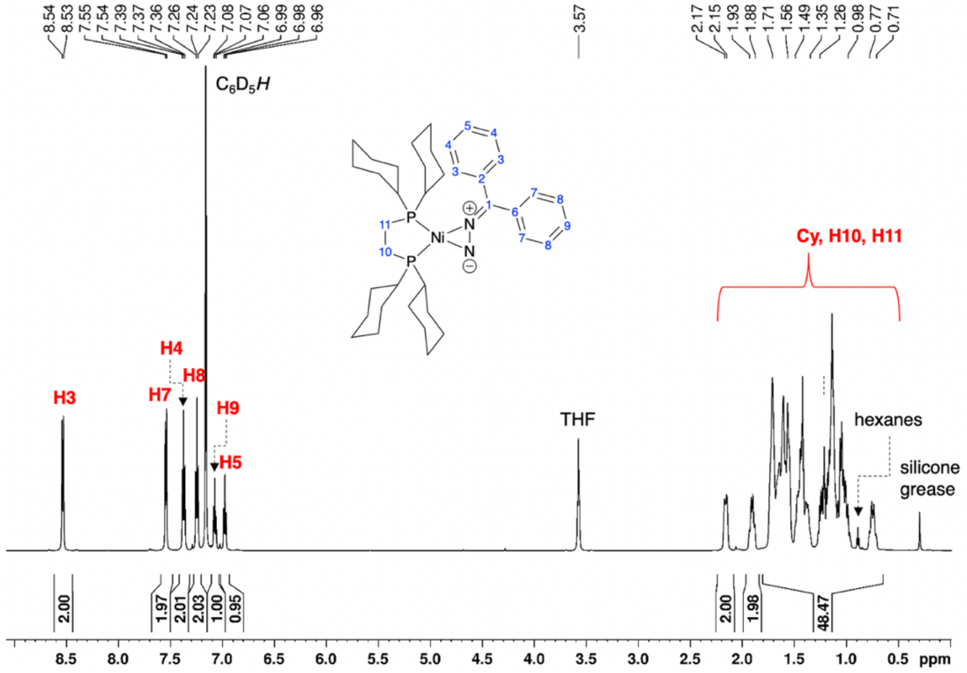

(b)

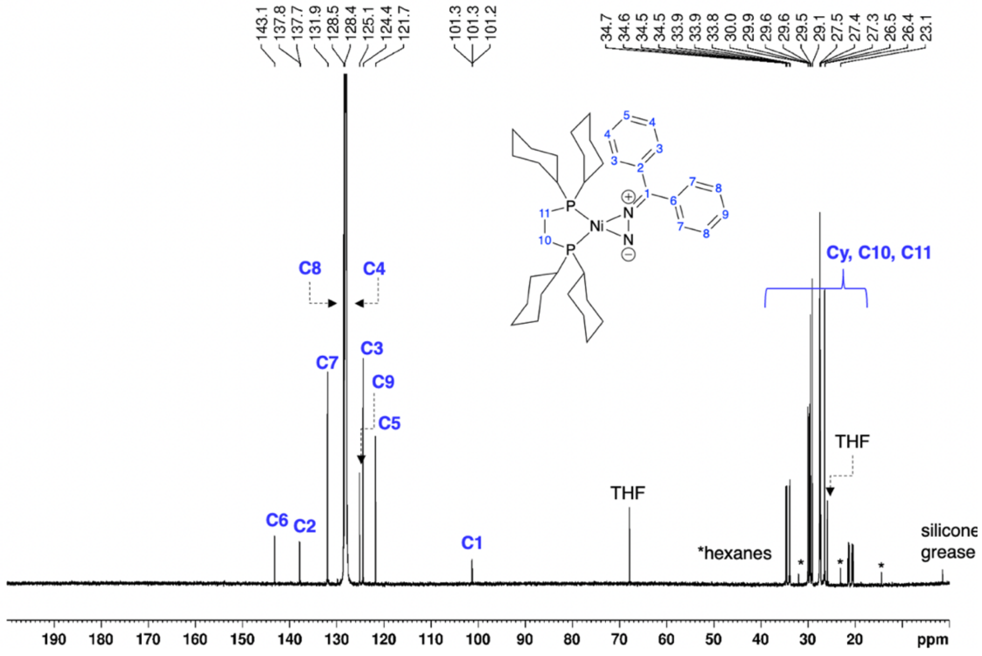

(c)

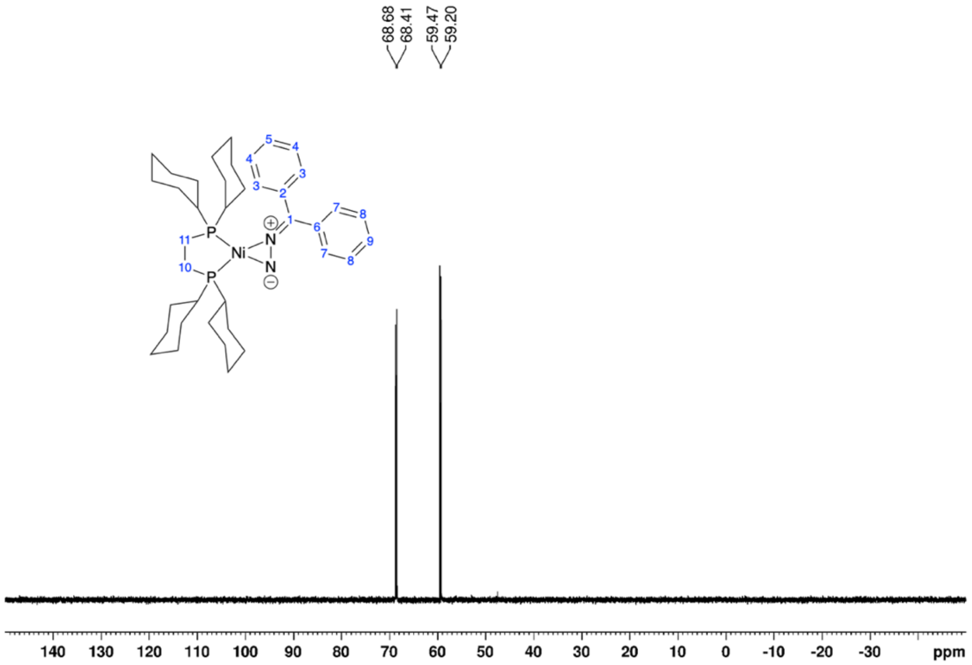
68.68
68.41
59.47
59.20
140 130 120 110 100 90 80 70 60 50 40 30 20 10 0 -10 -20 -30 ppm

(d)

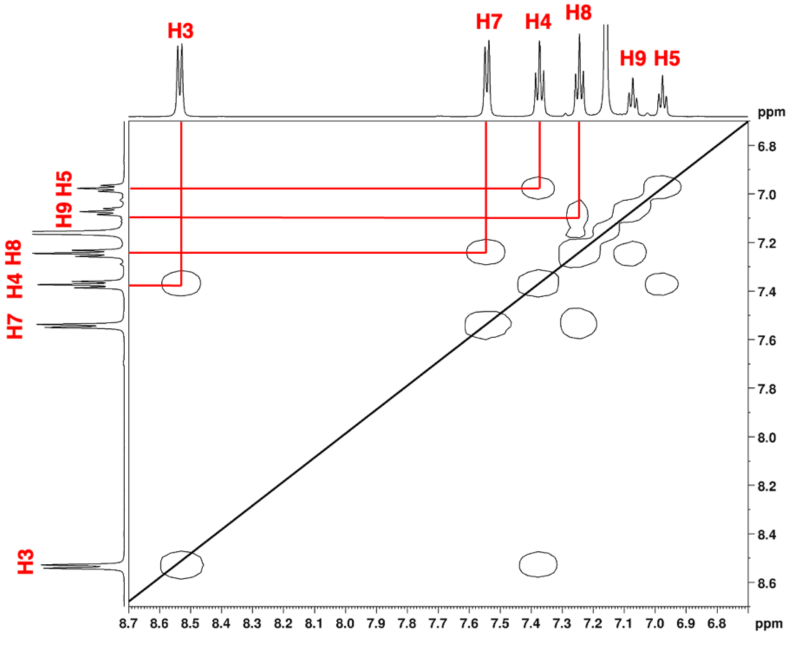
H3
H7
H4
H8
H9
H5
ppm

(e)

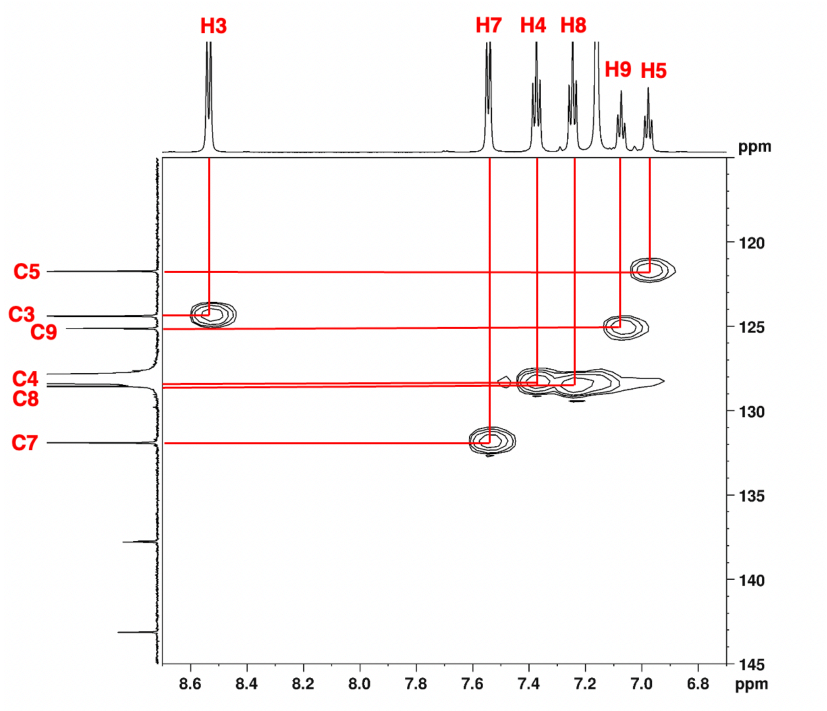

(f)

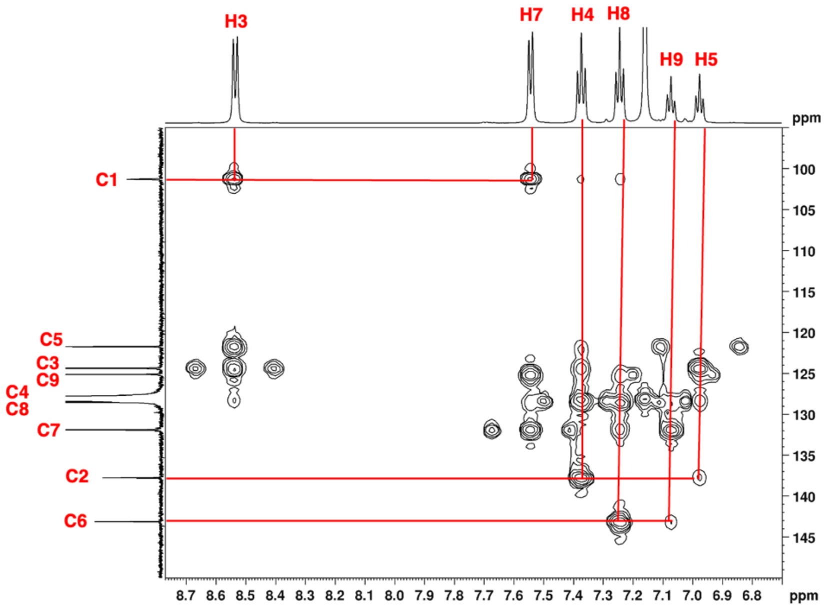

Figure S1. NMR spectra of **Ni-2b** (C_6D_6). ^{1}H NMR (600 MHz). (b) $^{13}C\{^1H\}$ (151 MHz). (c) $^{31}P\{^1H\}$ (202 MHz). (d) ^{1}H-^{1}H COSY expansion (600 MHz). (e) ^{1}H-^{13}C HSQC expansion (600 MHz, 151 MHz). (f) ^{1}H-^{13}C HMBC expansion (600 MHz, 151 MHz)

S2.1.2 Ni(dibpp)(N_2CPh_2), Ni-2c.

(a)

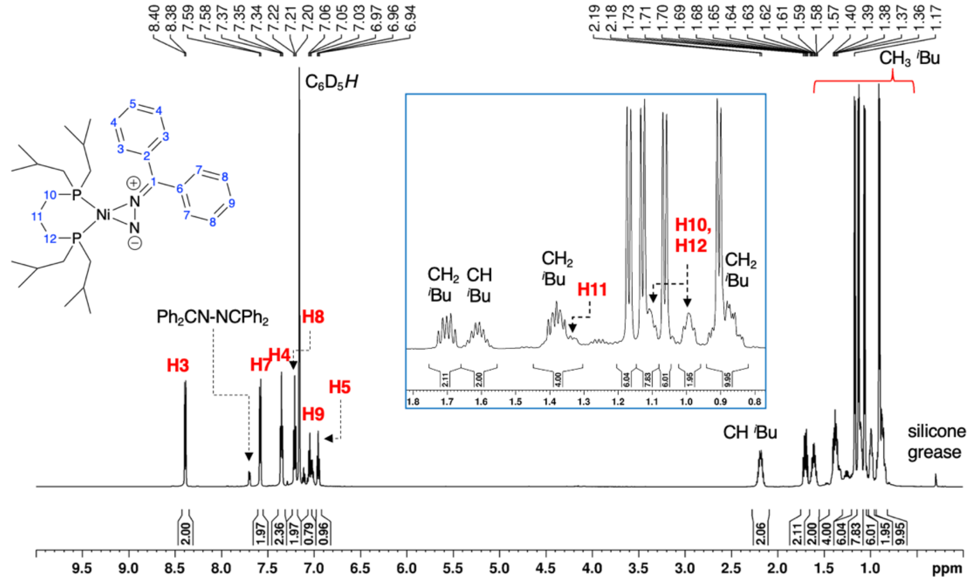

(b)

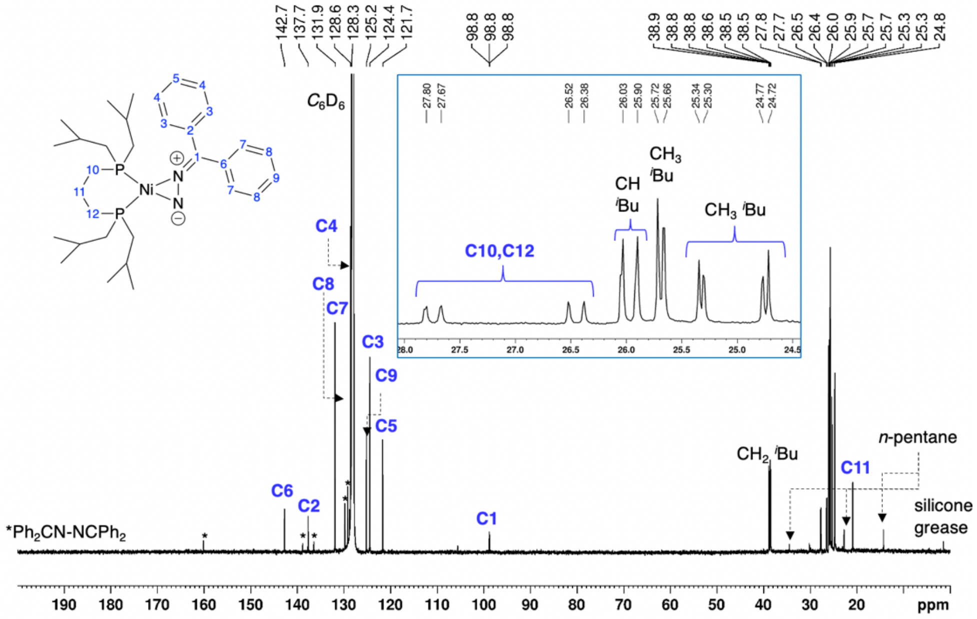

(c)

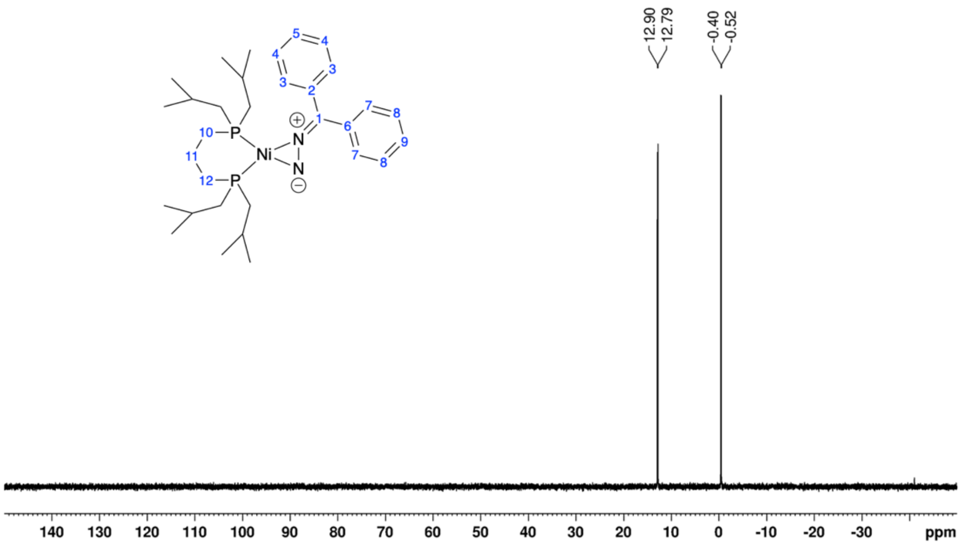

(d)

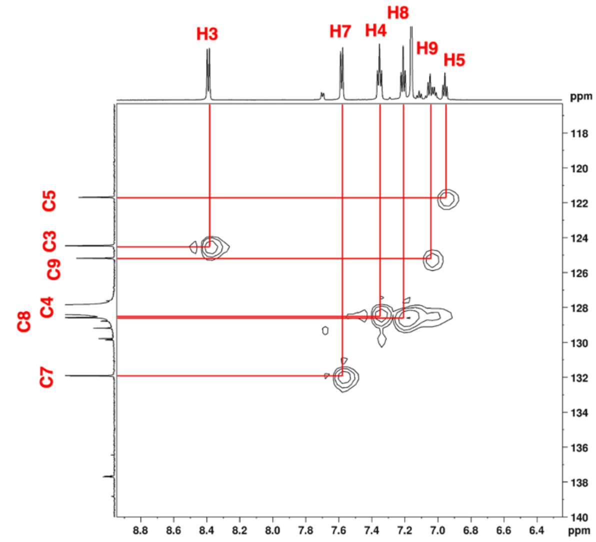

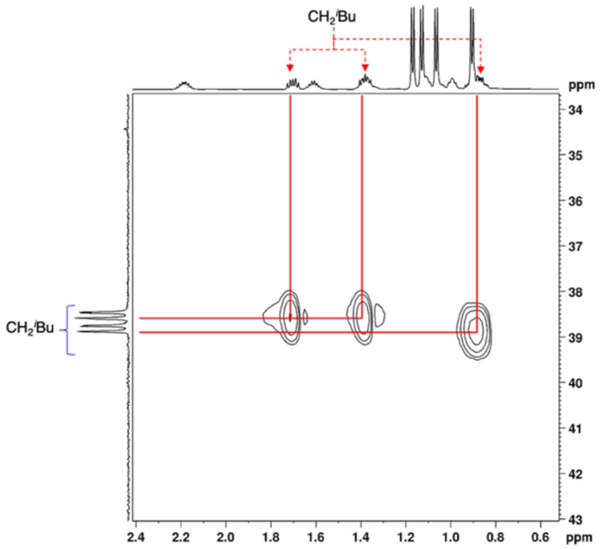

$CH_2{}^iBu$
$CH_2{}^iBu$
ppm

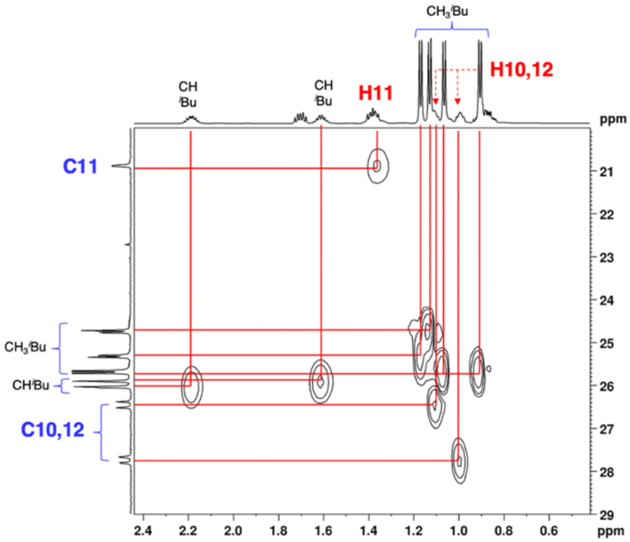

$CH_3{}^iBu$
CH ^iBu
CH ^iBu
H11
H10,12
C11
$CH_3{}^iBu$
CH^iBu
C10,12
ppm

(e)

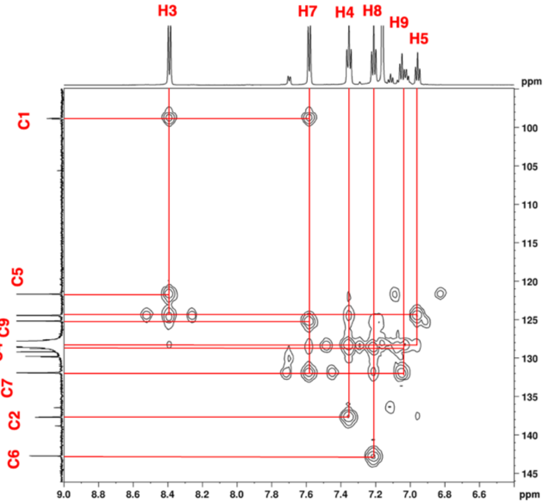

H3
H7
H4
H8
H9
H5
C1
C5
C3
C9
C4
C8
C7
C2
C6
ppm

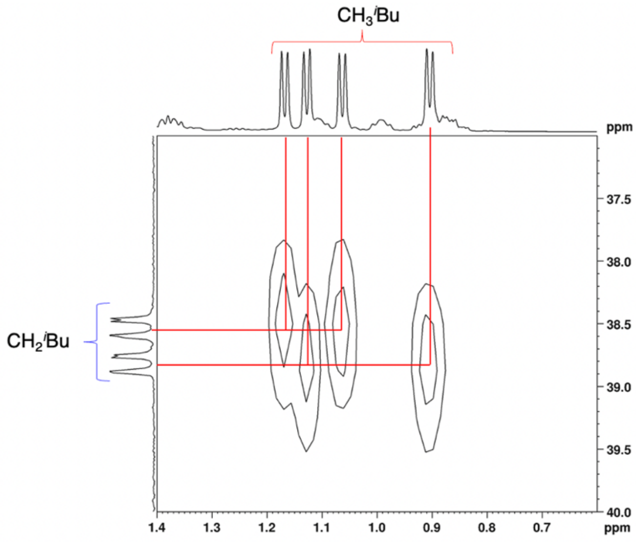

$CH_3{}^iBu$
$CH_2{}^iBu$
ppm

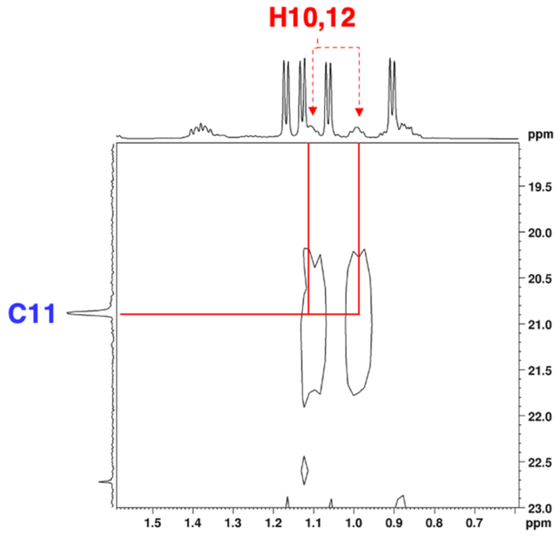

H10,12
C11
ppm

(f)

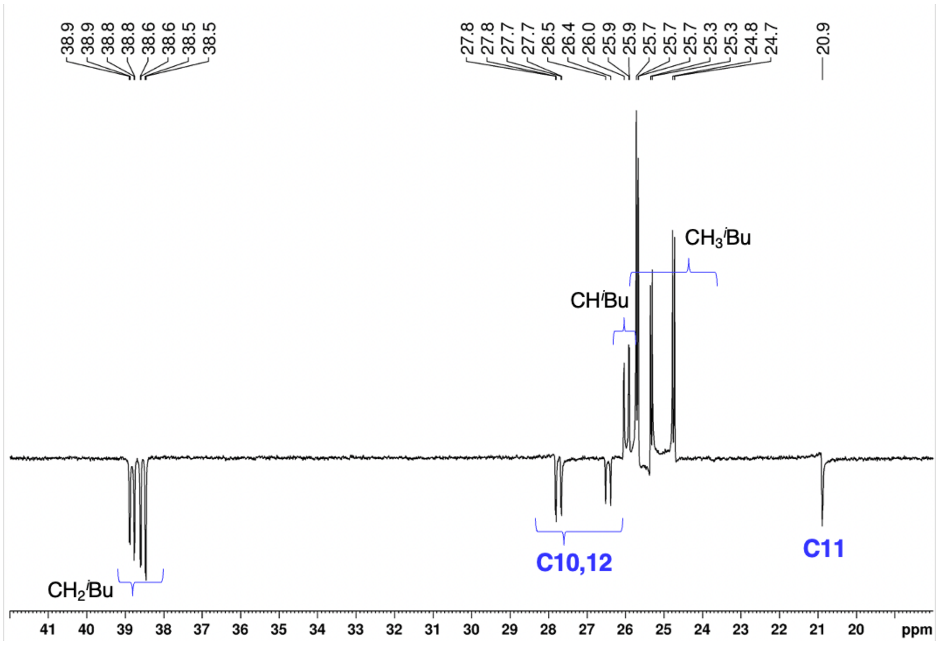

Figure S2. NMR spectra of **Ni-2c** in C_6D_6. (a) ^{1}H NMR (600 MHz). (b) $^{13}C\{^1H\}$ (151 MHz). (c) $^{31}P\{^1H\}$ (202 MHz). (d) ^{1}H-^{13}C HSQC expansion (600 MHz, 150 MHz). (e) ^{1}H-^{13}C HMBC expansion (600 MHz, 150 MHz). (f) $^{13}C\{^1H\}$ DEPT (151 MHz)

S2.1.3 Ni(dppf)(N_2CPh_2), Ni-2d.

(a)

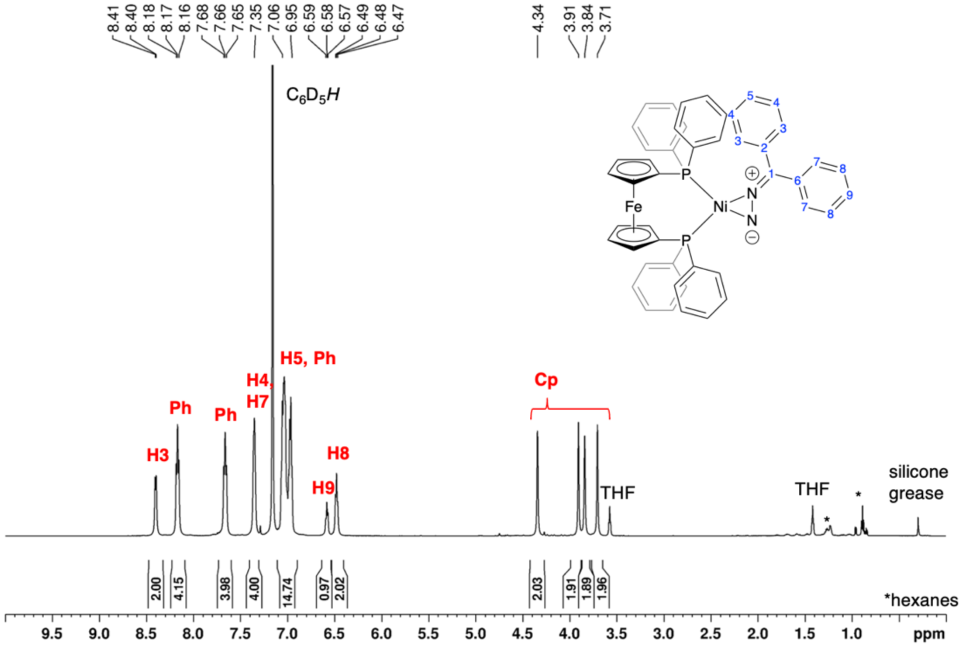

(b)

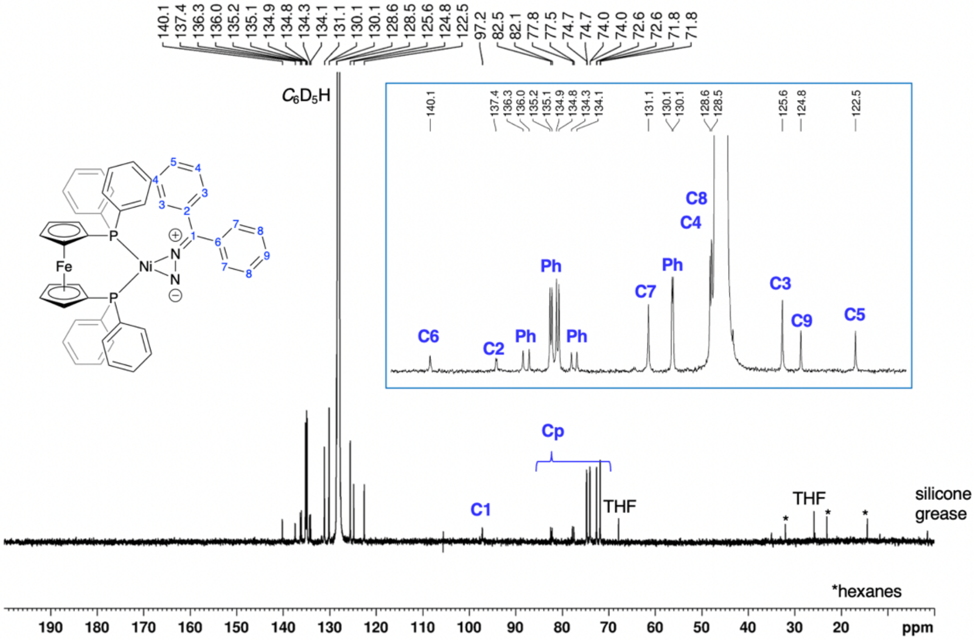
140.1 137.4 136.3 136.0 135.2 135.1 134.9 134.8 134.3 134.1 131.1 130.1 130.1 128.6 128.5 125.6 124.8 122.5 97.2 82.5 82.1 77.8 77.5 74.7 74.7 74.0 74.0 72.6 72.6 71.8 71.8
C_6D_5H
Fe
Ni
P
N
C6
C2
Ph
Ph
Ph
C7
Ph
C8
C4
C3
C9
C5
Cp
C1
THF
THF
silicone grease
*hexanes
ppm

(c)

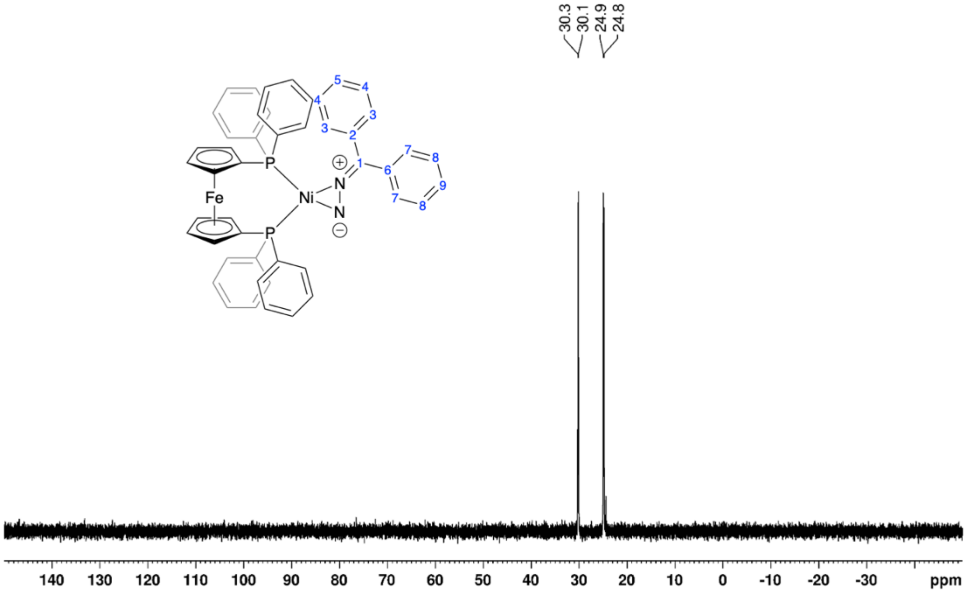
30.3 30.1 24.9 24.8
Fe
Ni
P
N
ppm

(d)

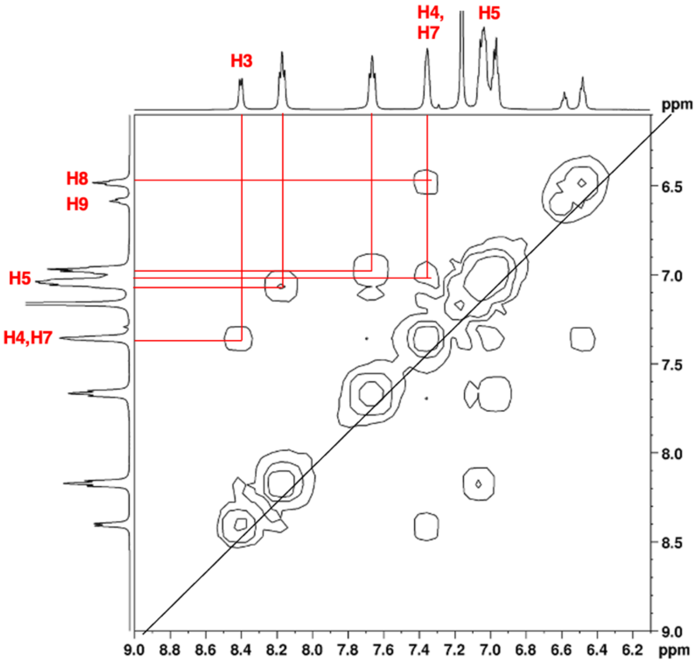
H3
H4,
H7
H5
ppm
H8
H9
H5
H4,H7
6.5
7.0
7.5
8.0
8.5
9.0
9.0 8.8 8.6 8.4 8.2 8.0 7.8 7.6 7.4 7.2 7.0 6.8 6.6 6.4 6.2
ppm

(e)

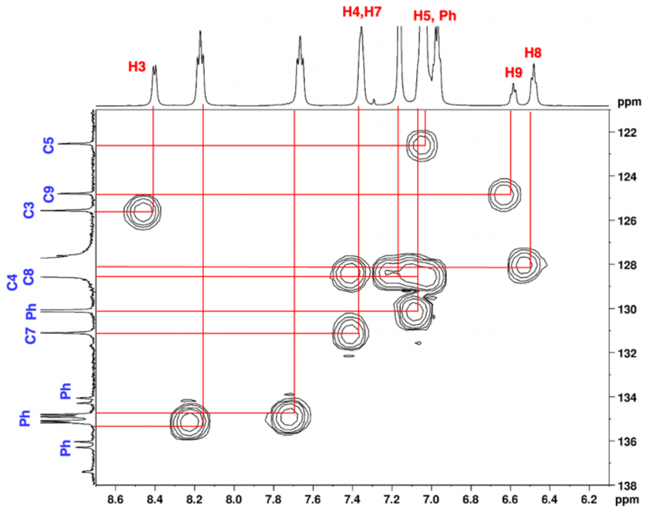
H4,H7
H5, Ph
H8
H3
H9
ppm
C5
C9
C3
C4
C8
Ph
C7
Ph
Ph
Ph
122
124
126
128
130
132
134
136
138
8.6 8.4 8.2 8.0 7.8 7.6 7.4 7.2 7.0 6.8 6.6 6.4 6.2
ppm

(f)

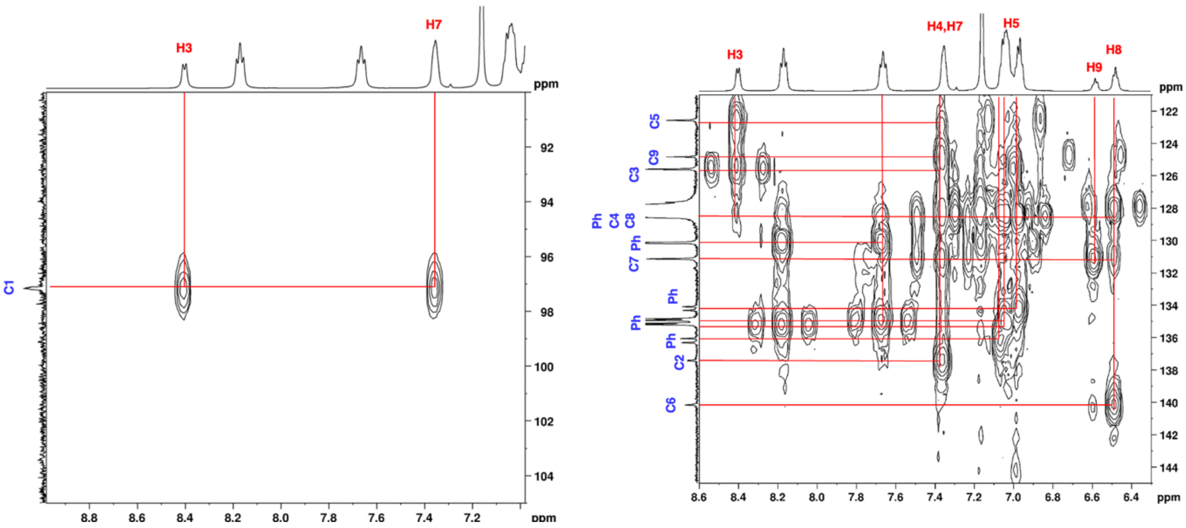

Figure S3. NMR spectra of **Ni-2d** in C_6D_6. (a) ^{1}H NMR (600 MHz). (b) $^{13}C\{^1H\}$ (151 MHz). (c) $^{31}P\{^1H\}$ (202 MHz). (d) ^{1}H-^{1}H COSY expansion (600 MHz). (e) ^{1}H-^{13}C HSQC expansion (600 MHz, 151 MHz). (f) ^{1}H-^{13}C HMBC expansion (600 MHz, 151 MHz)

S2.1.4 Ni(dpephos)(N_2CPh_2), Ni-2e.

(a)

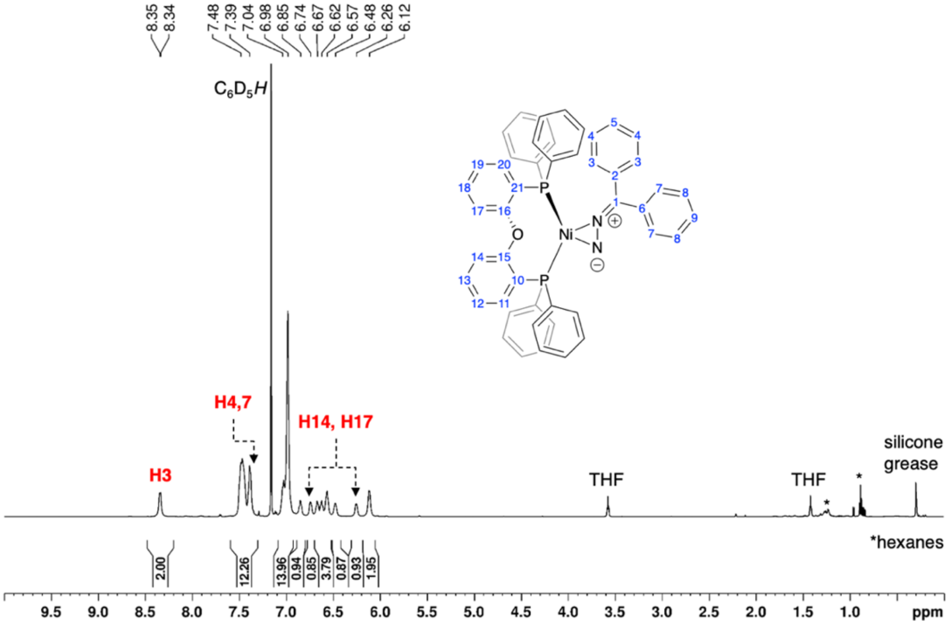

(b)

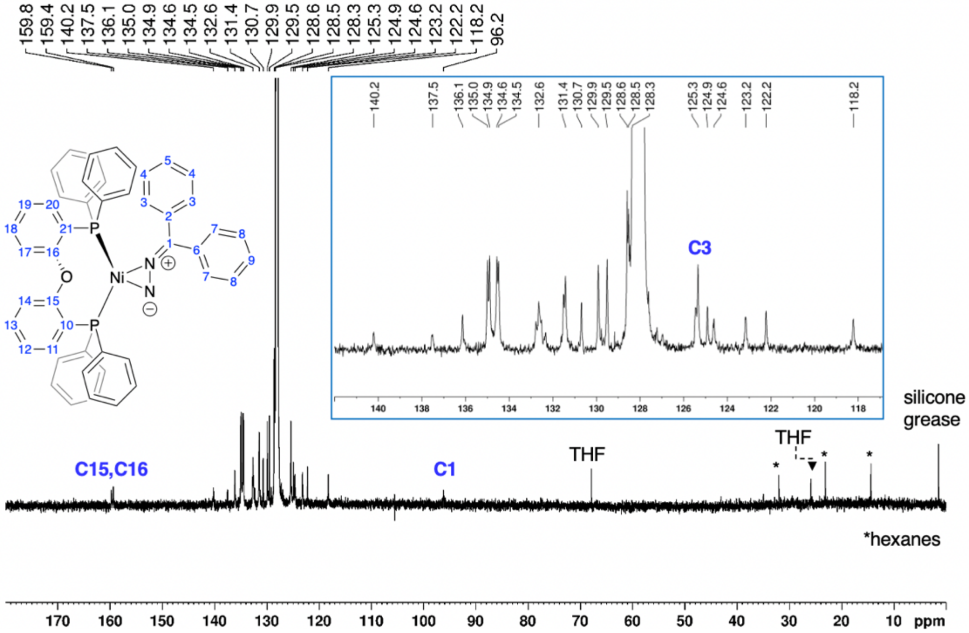
159.8
159.4
140.2
137.5
136.1
135.0
134.9
134.6
134.5
132.6
131.4
130.7
129.9
129.5
128.6
128.5
128.3
125.3
124.9
124.6
123.2
122.2
118.2
96.2
C3
C15,C16
C1
THF
THF
silicone grease
*hexanes
ppm

(c)

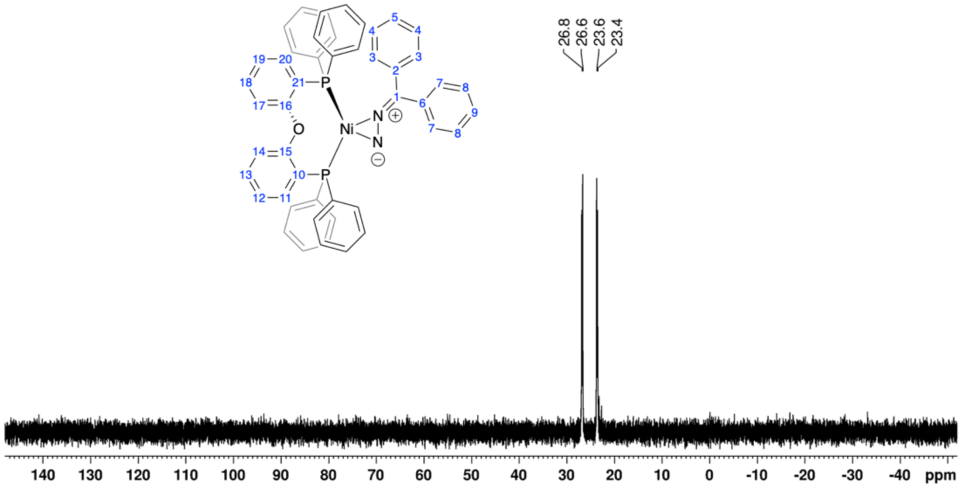
26.8
26.6
23.6
23.4
ppm

(d)

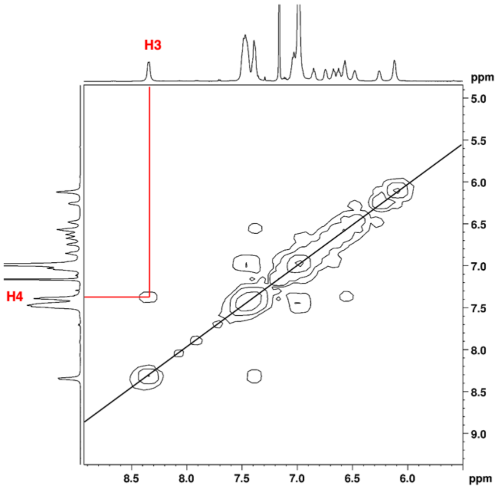

(e)

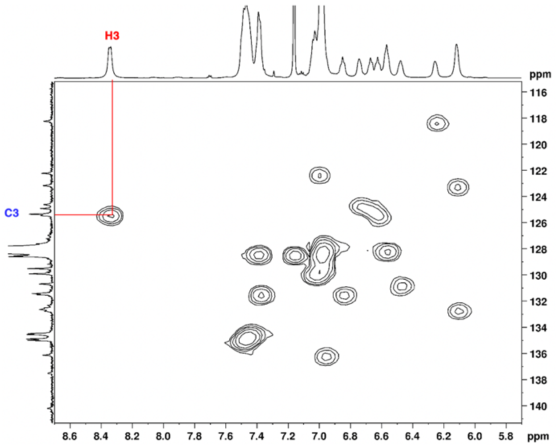

(f)

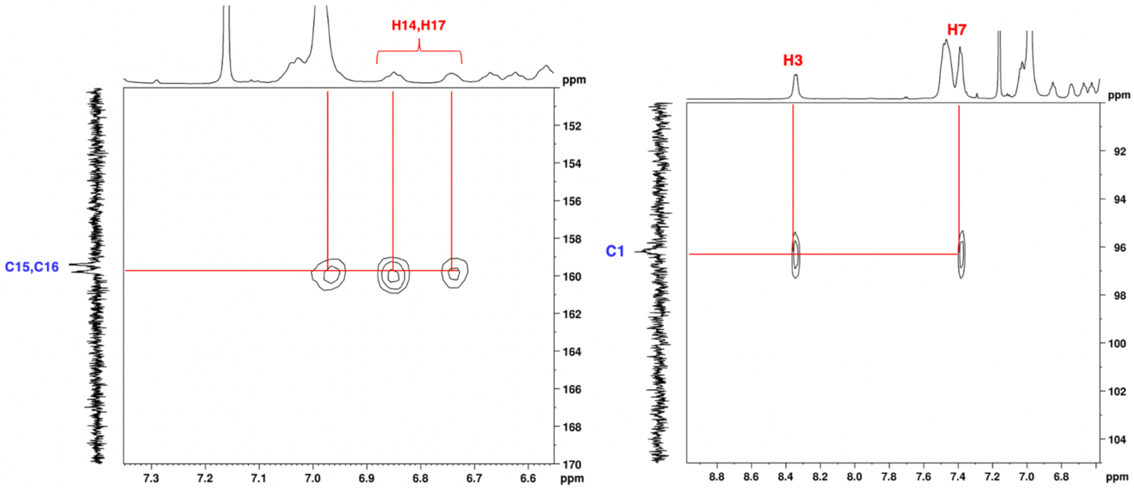

Figure S4. NMR spectra of **Ni-2e** in C_6D_6. (a) ^{1}H NMR (600 MHz). (b) $^{13}C\{^1H\}$ (151 MHz). (c) $^{31}P\{^1H\}$ (202 MHz). (d) ^{1}H-^{1}H COSY expansion (600 MHz). (e) ^{1}H-^{13}C HSQC expansion (600 MHz, 151 MHz). (f) ^{1}H-^{13}C HMBC expansion (600 MHz, 151 MHz)

S2.2 In Situ Characterization of Carbene Complexes Ni-1

S2.2.1 Ni(dcpe)(CPh₂), Ni-1b

(a)

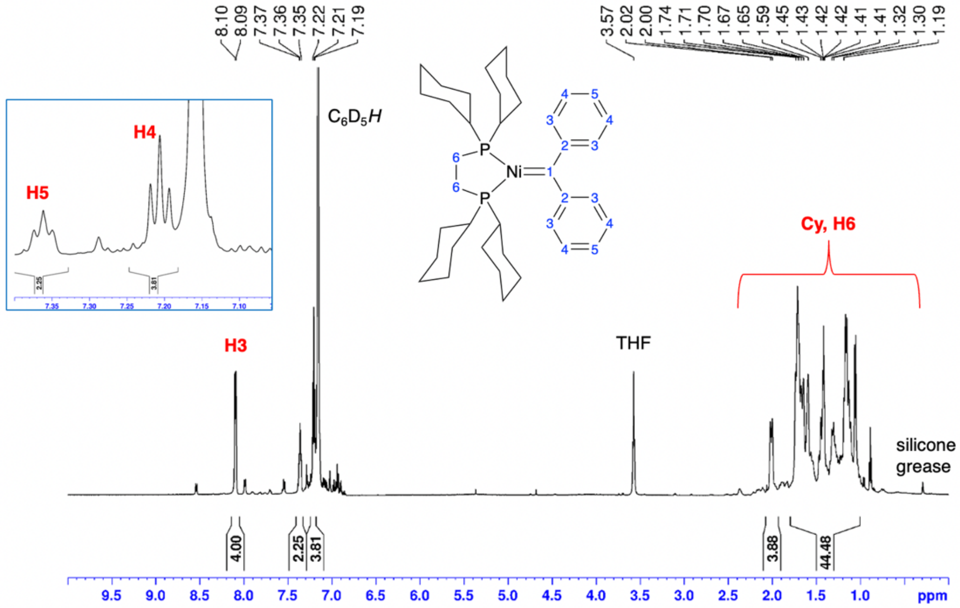

(b)

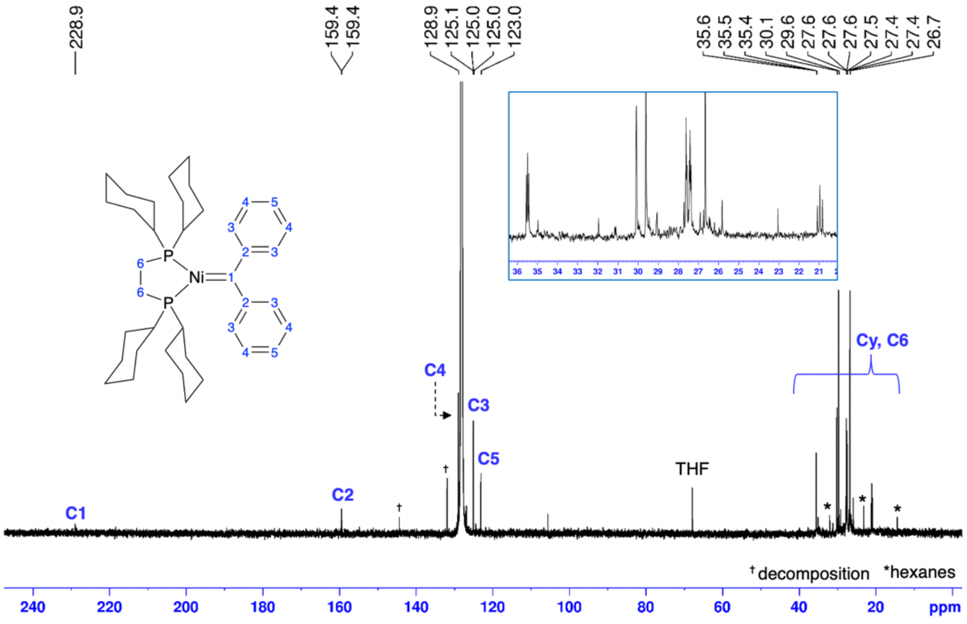

(c)

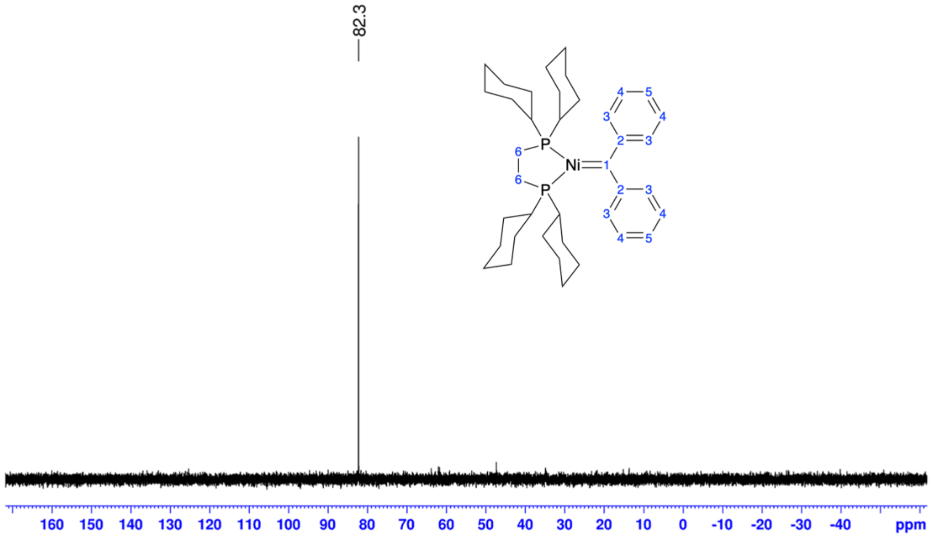
82.3
P
P
Ni
1
2
3
4
5
6
6
160
150
140
130
120
110
100
90
80
70
60
50
40
30
20
10
0
-10
-20
-30
-40
ppm

(d)

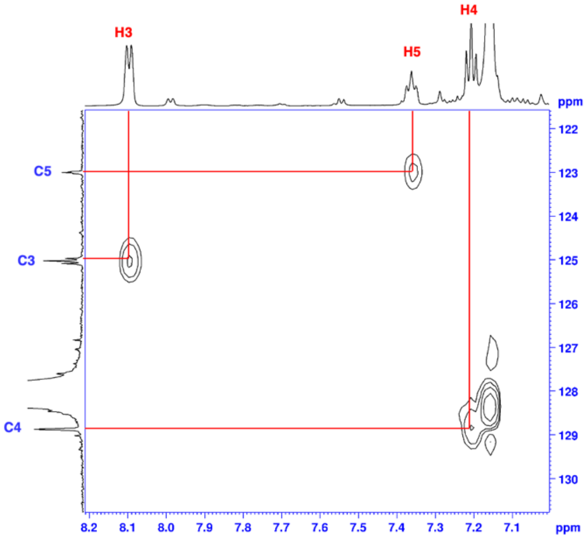
H3
H5
H4
C5
C3
C4
ppm
122
123
124
125
126
127
128
129
130
8.2
8.1
8.0
7.9
7.8
7.7
7.6
7.5
7.4
7.3
7.2
7.1
ppm

(e)

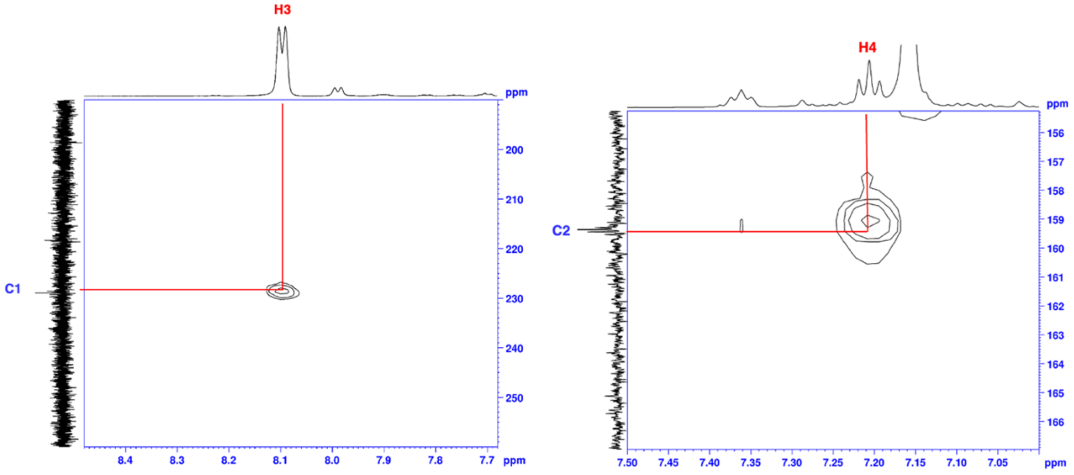

Figure S5. NMR spectra of **Ni-1b** in C_6D_6. (a) ^{1}H NMR (600 MHz). (b) $^{13}C\{^1H\}$ (151 MHz). (c) $^{31}P\{^1H\}$ (202 MHz). (d) ^{1}H-^{13}C HSQC expansion (600 MHz, 151 MHz). (e) ^{1}H-^{13}C HMBC expansion (600 MHz, 151 MHz)

S2.2.2 Ni(dibpp)(CPh_2), Ni-1c

(a)

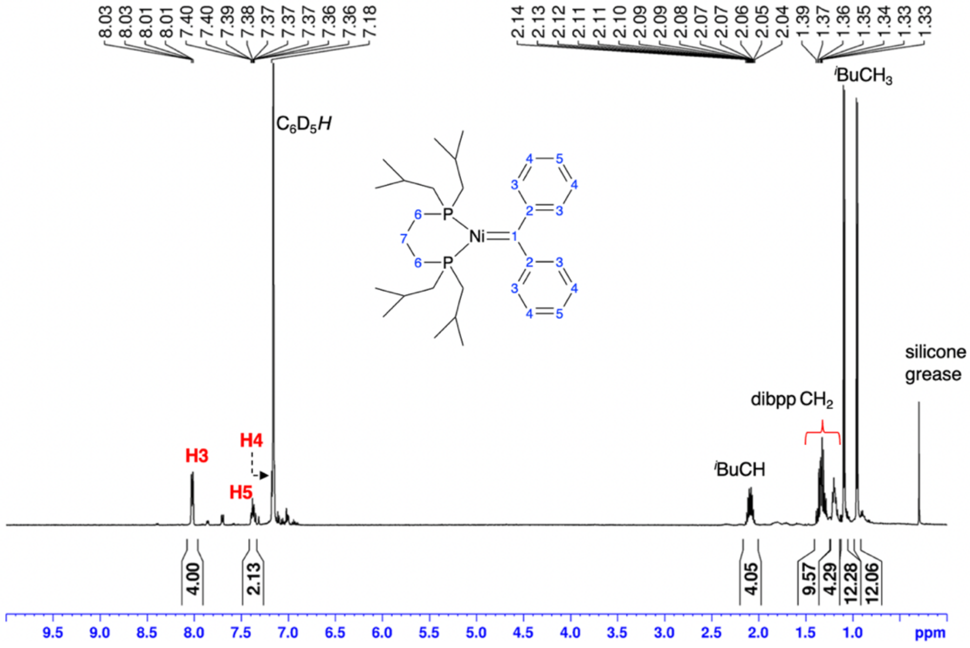

(b)

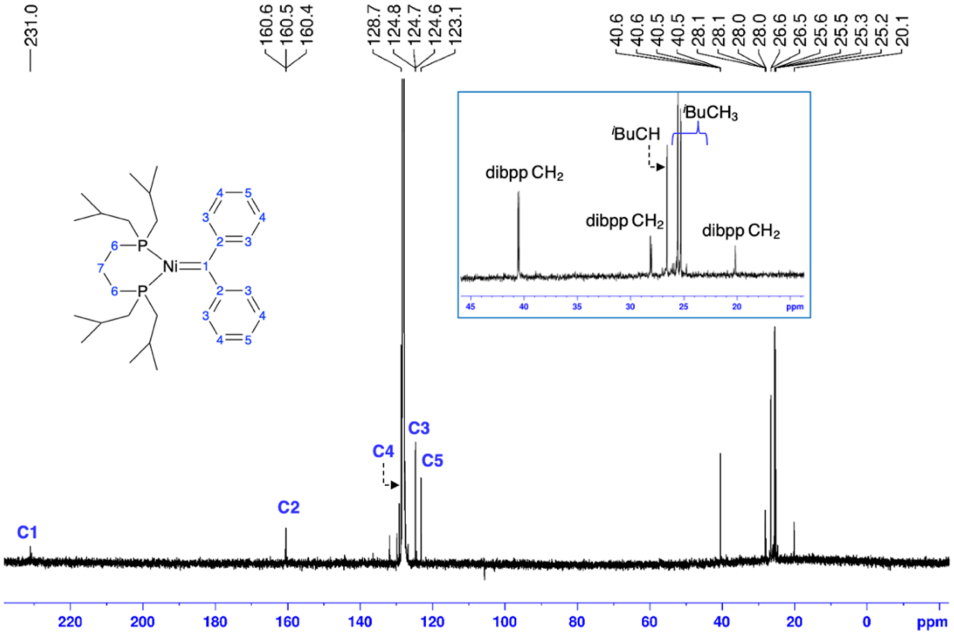
231.0
160.6
160.5
160.4
128.7
124.8
124.7
124.6
123.1
40.6
40.6
40.5
40.5
28.1
28.1
28.0
28.0
26.6
26.5
25.6
25.5
25.3
25.2
20.1
P
Ni
P
1
2
3
4
5
6
7
ⁱBuCH
ⁱBuCH₃
dibpp CH₂
dibpp CH₂
dibpp CH₂
C1
C2
C3
C4
C5
ppm

(c)

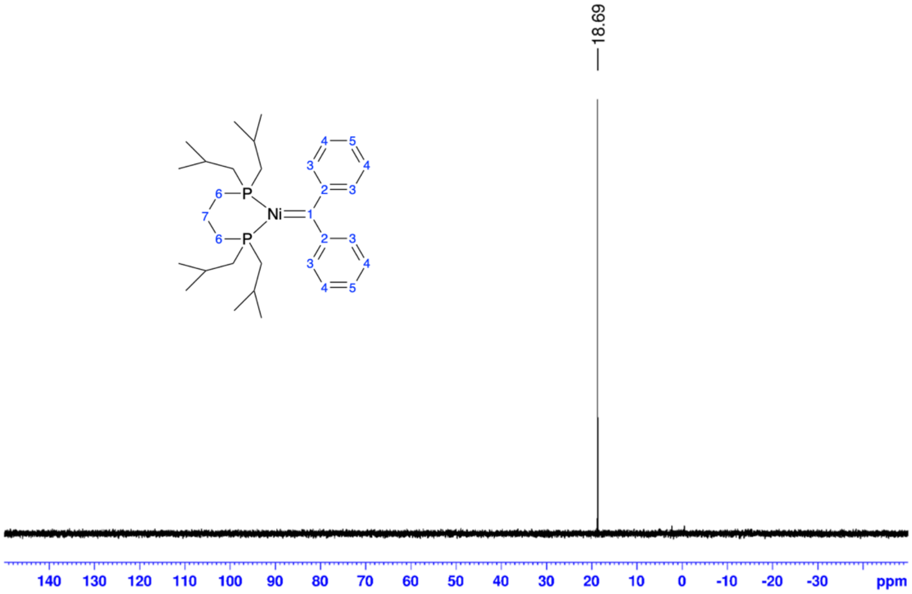
18.69
P
Ni
P
1
2
3
4
5
6
7
ppm

(d)

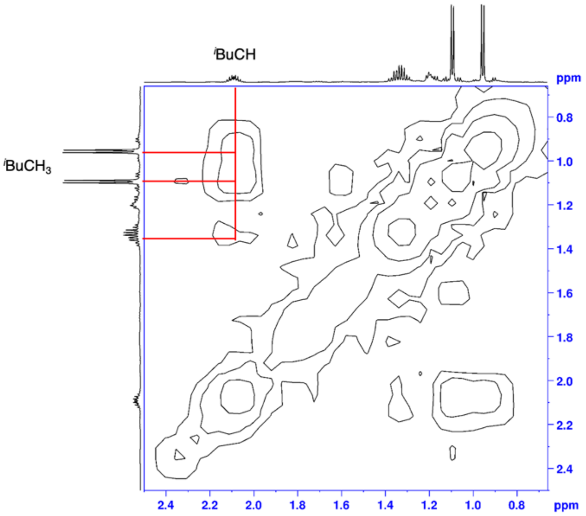

iBuCH
iBuCH3
ppm

(e)

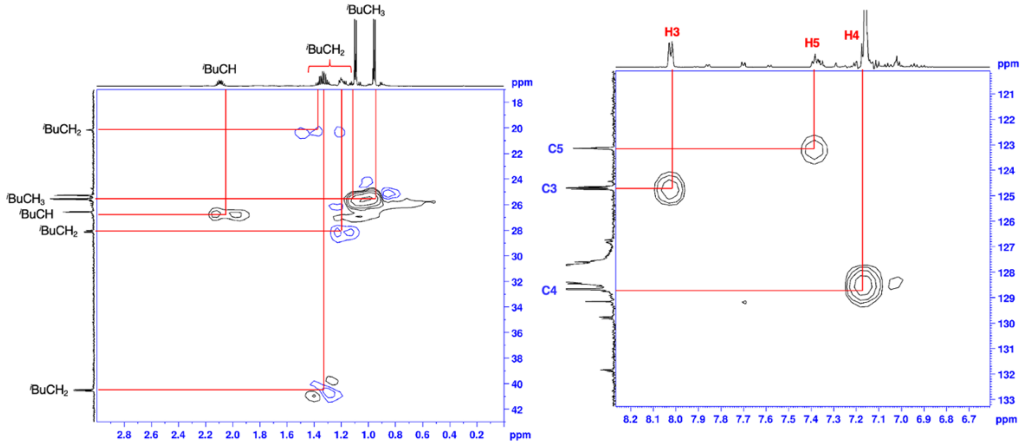

iBuCH
iBuCH2
iBuCH3
iBuCH2
iBuCH3
iBuCH
iBuCH2
iBuCH2
H3
H5
H4
C5
C3
C4
ppm

(f)

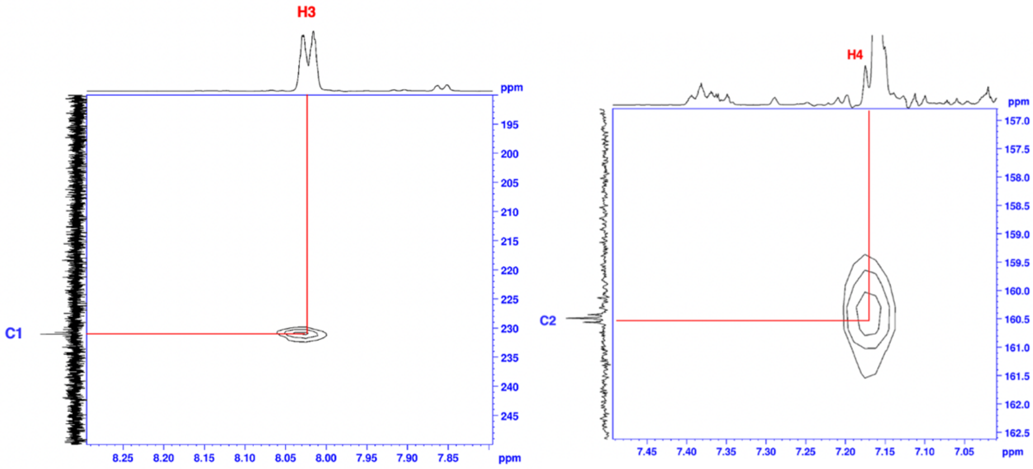

H3
C1
H4
C2
ppm

Figure S6. NMR spectra of **Ni-1c** in C_6D_6. (a) ^1H NMR (500 MHz). (b) $^{13}C\{^1H\}$ (151 MHz). (c) $^{31}P\{^1H\}$ (202 MHz). (d) ^1H-^1H COSY expansion (600 MHz). (e) ^1H-^{13}C HSQC expansion (600 MHz, 151 MHz). (f) ^1H-^{13}C HMBC expansion (600 MHz, 151 MHz)

S2.2.3 Ni(dppf)(CPh₂), Ni-1d

(a)

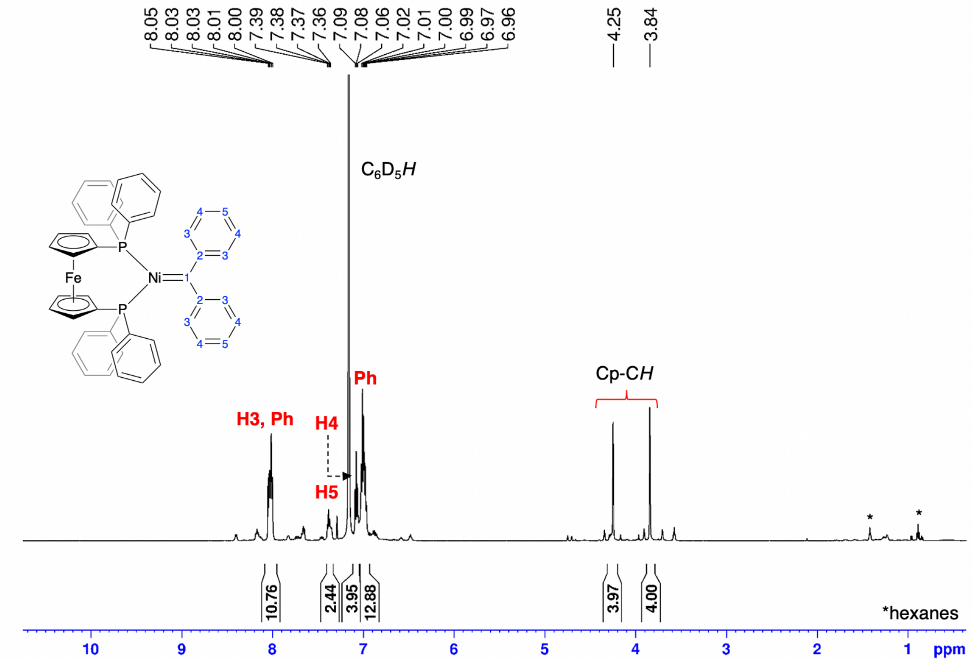

(b)

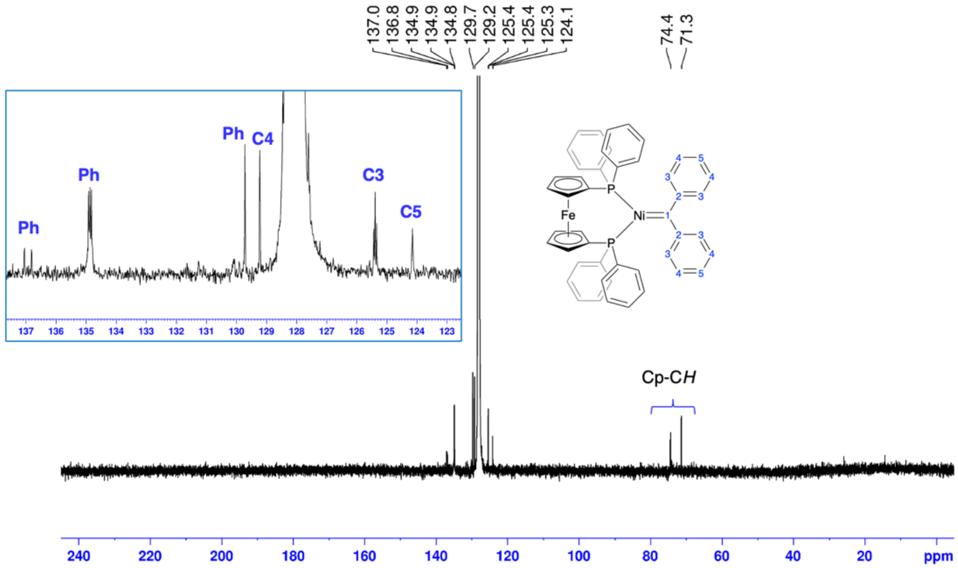

(c)

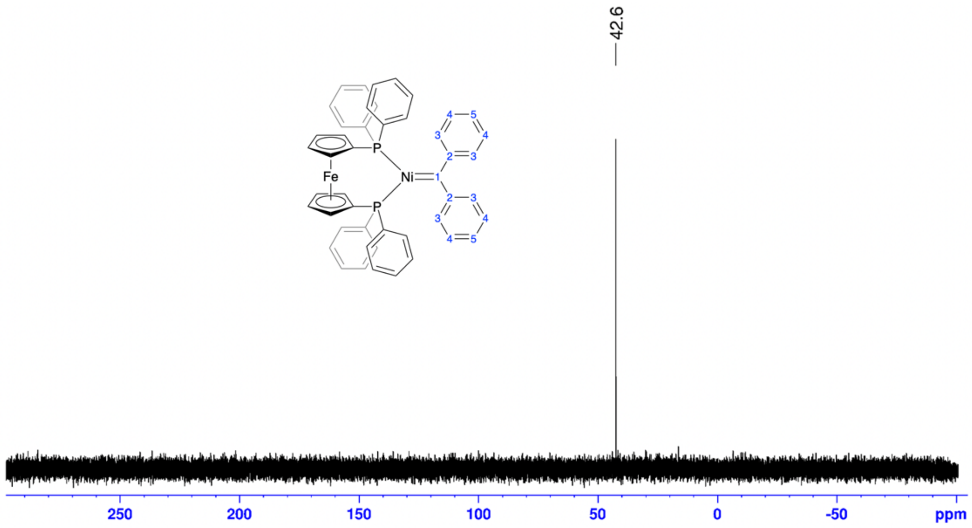
42.6
Fe
P
Ni
1
2
3
4
5
250
200
150
100
50
0
-50
ppm

(d)

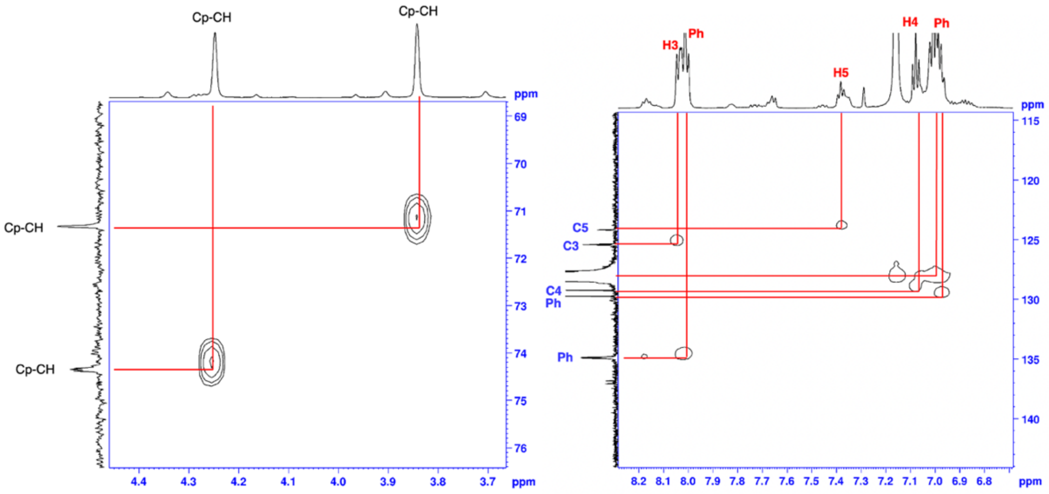
Cp-CH
Cp-CH
Cp-CH
Cp-CH
ppm
69
70
71
72
73
74
75
76
4.4
4.3
4.2
4.1
4.0
3.9
3.8
3.7
H3
Ph
H5
H4
Ph
C5
C3
C4
Ph
Ph
115
120
125
130
135
140
8.2
8.1
8.0
7.9
7.8
7.7
7.6
7.5
7.4
7.3
7.2
7.1
7.0
6.9
6.8

(e)

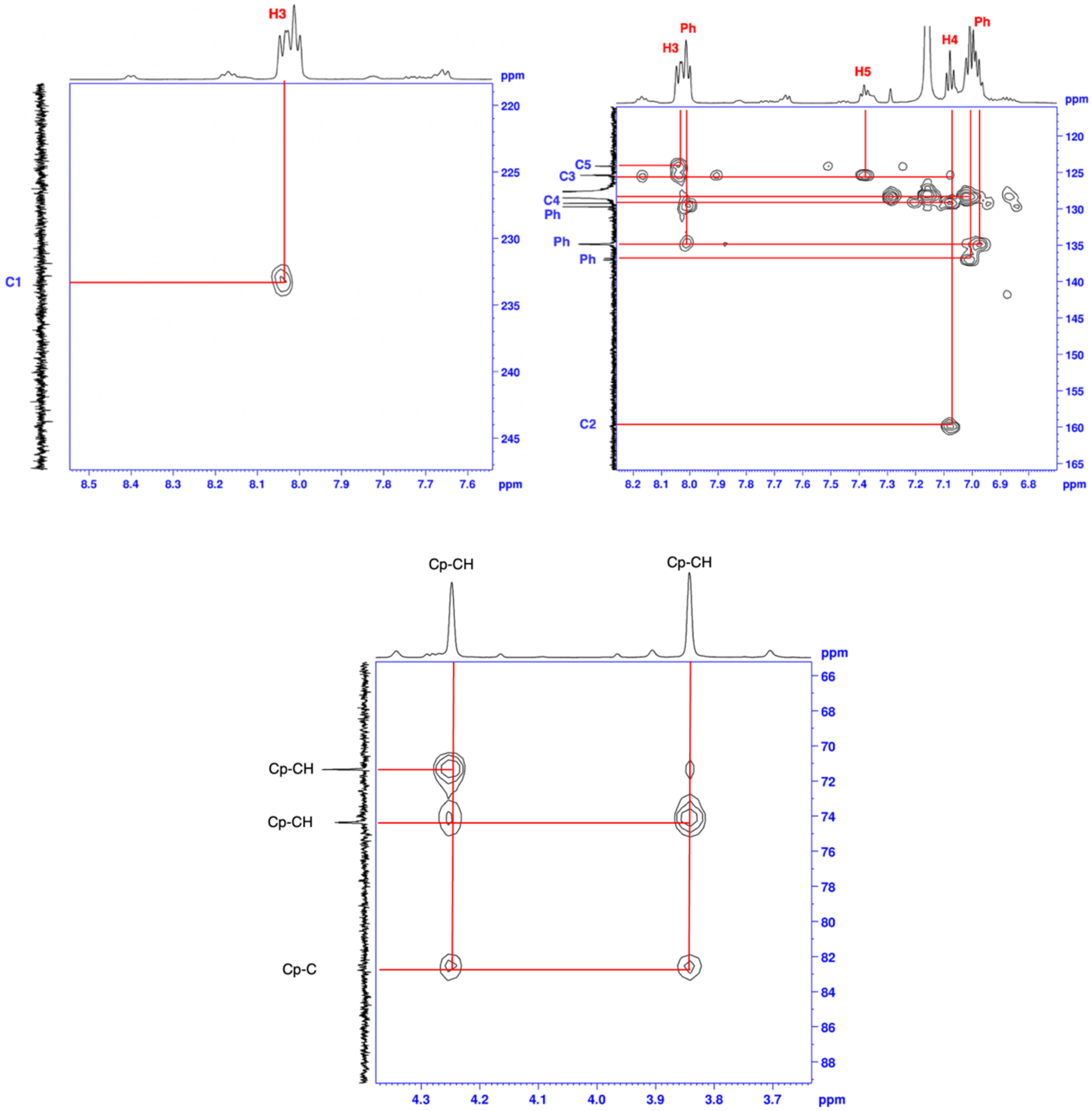

Figure S7. NMR spectra of **Ni-1d** in C_6D_6. (a) ^1H NMR (600 MHz). (b) $^{13}C\{^1H\}$ (151 MHz). (c) $^{31}P\{^1H\}$ (202 MHz). (d) ^1H-^{13}C HSQC expansion (600 MHz, 151 MHz). (e) ^1H-^{13}C HMBC expansion (600 MHz, 151 MHz)

S2.2.4 Ni(dpephos)(CPh_2), Ni-1e

(a)

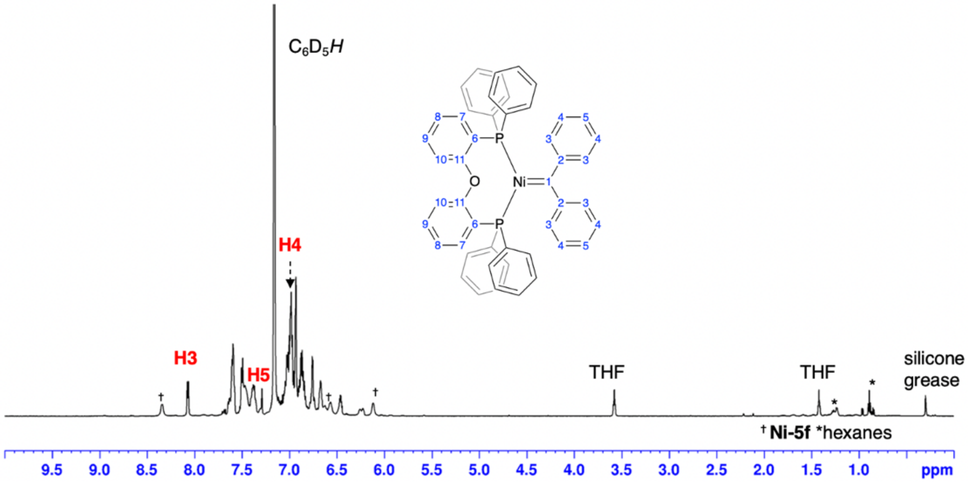

(b)

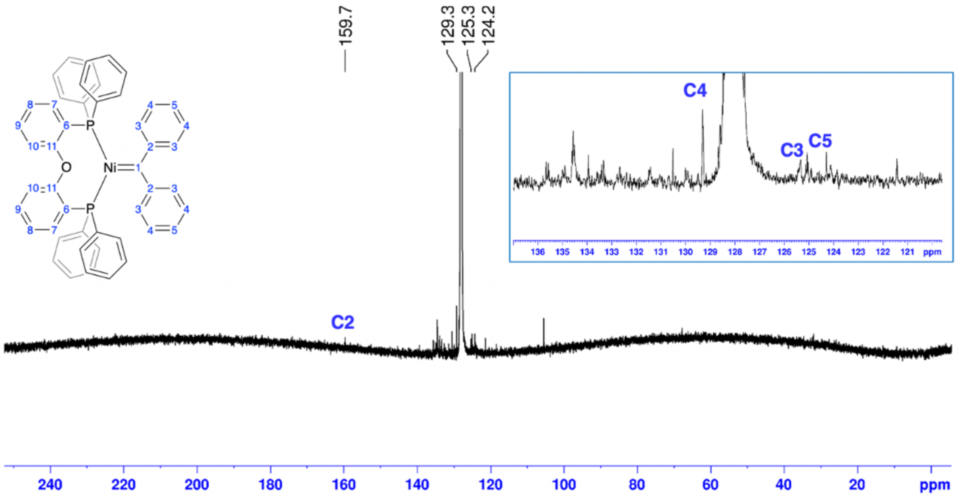

(c)

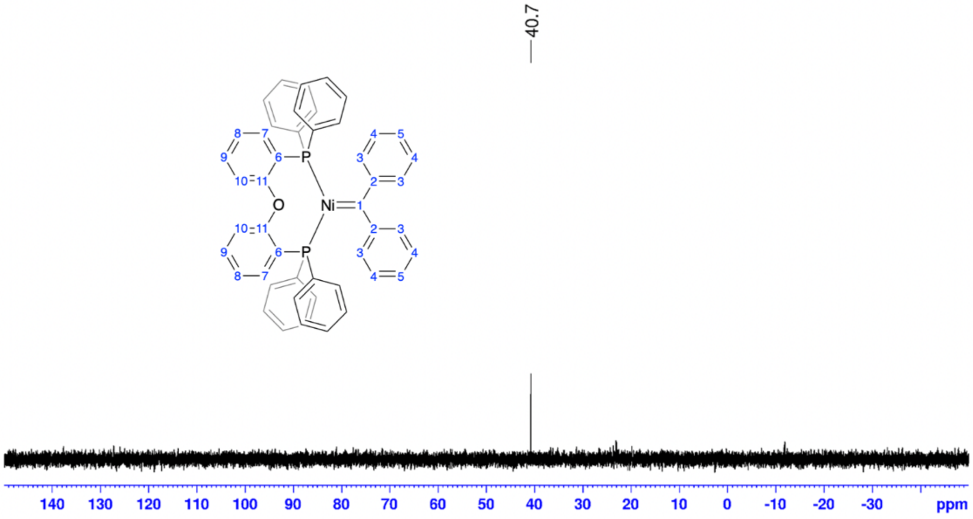

(d)

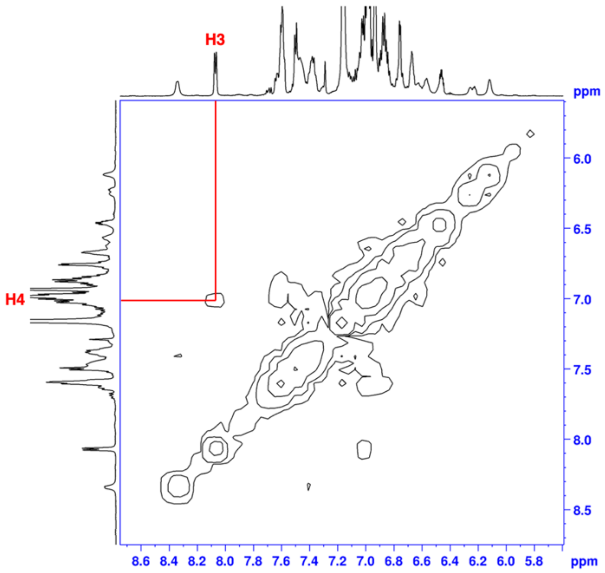
H3
H4
ppm
6.0
6.5
7.0
7.5
8.0
8.5
8.6 8.4 8.2 8.0 7.8 7.6 7.4 7.2 7.0 6.8 6.6 6.4 6.2 6.0 5.8
ppm

(e)

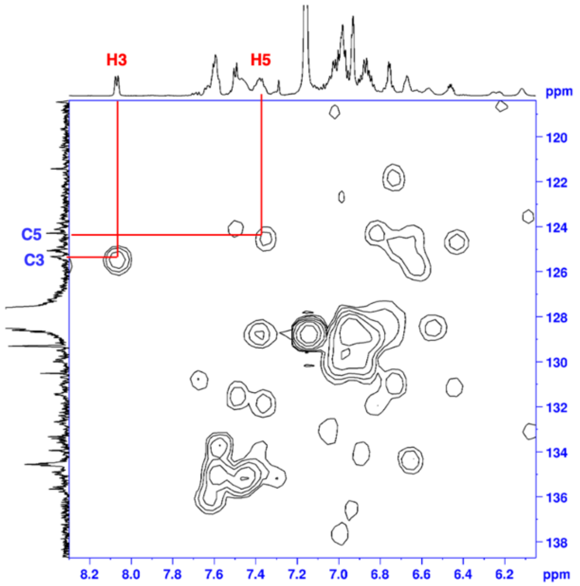
H3
H5
ppm
C5
C3
120
122
124
126
128
130
132
134
136
138
8.2 8.0 7.8 7.6 7.4 7.2 7.0 6.8 6.6 6.4 6.2
ppm

(f)

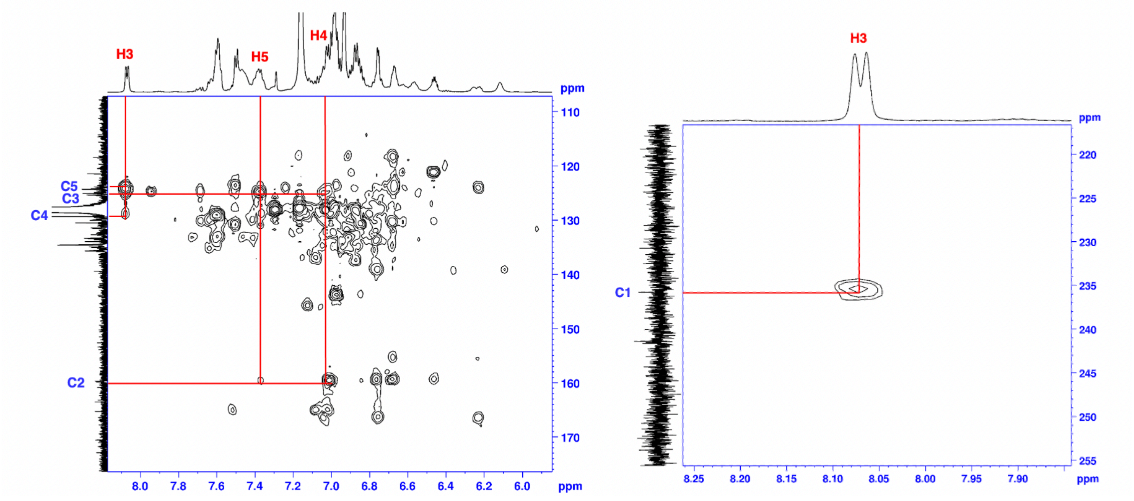

Figure S8. NMR spectra of **Ni-1e** in C_6D_6. (a) ^{1}H NMR (600 MHz). (b) $^{13}C\{^1H\}$ (151 MHz). (c) $^{31}P\{^1H\}$ (202 MHz). (d) ^{1}H-^{1}H COSY expansion (600 MHz). (e) ^{1}H-^{13}C HSQC expansion (600 MHz, 151 MHz). (f) ^{1}H-^{13}C HMBC expansion (600 MHz, 151 MHz)

S2.3 In Situ Characterization of NiCB Complex Ni-5c

S2.3.1 Ni(dibpp)[$CPh_2CH_2CH(SO_2Ph)$], Ni-5c

(a)

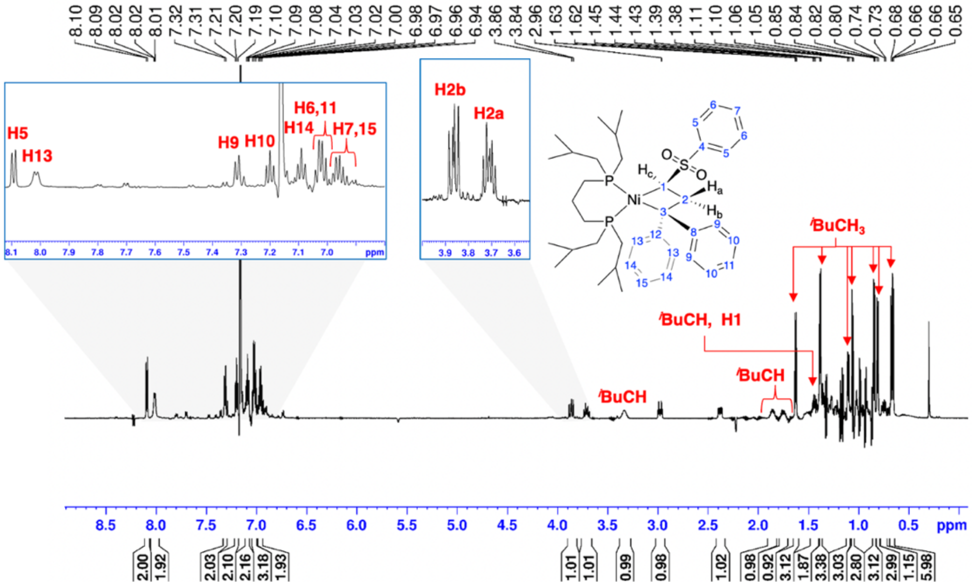

(b)

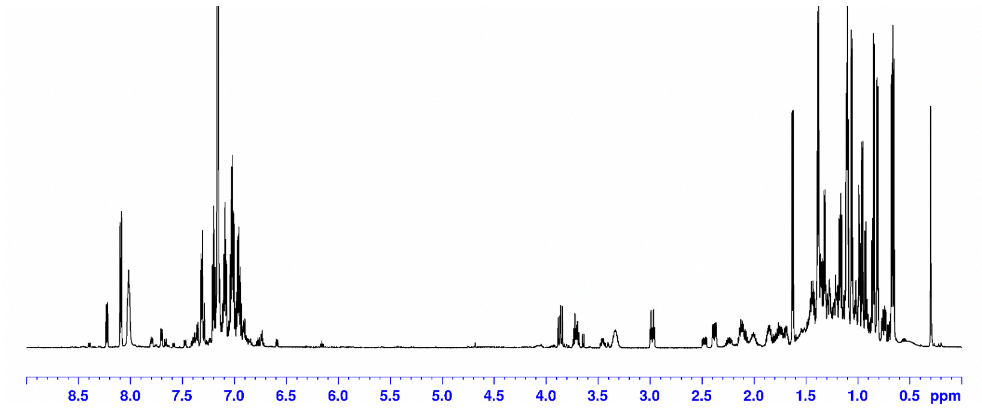

8.5
8.0
7.5
7.0
6.5
6.0
5.5
5.0
4.5
4.0
3.5
3.0
2.5
2.0
1.5
1.0
0.5
ppm

(c)

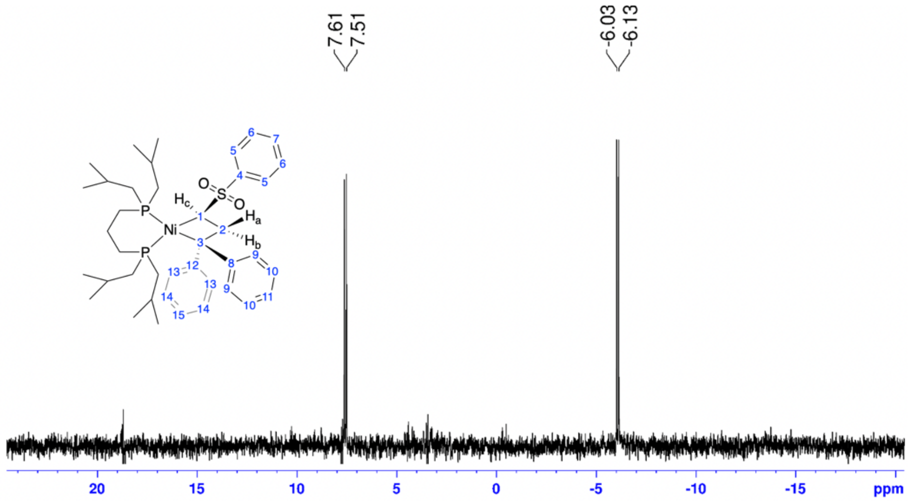

7.61
7.51
-6.03
-6.13
20
15
10
5
0
-5
-10
-15
ppm

(d)

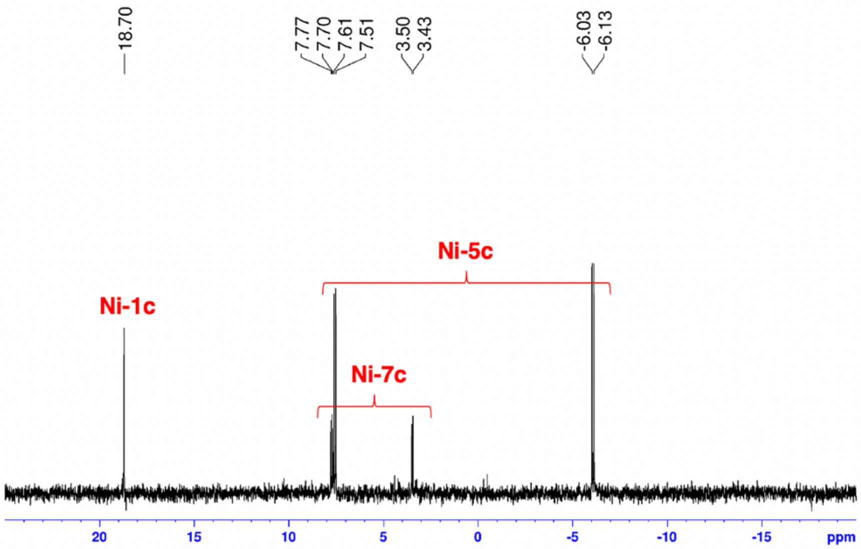

18.70
7.77
7.70
7.61
7.51
3.50
3.43
-6.03
-6.13
Ni-1c
Ni-5c
Ni-7c
20
15
10
5
0
-5
-10
-15
ppm

(e)

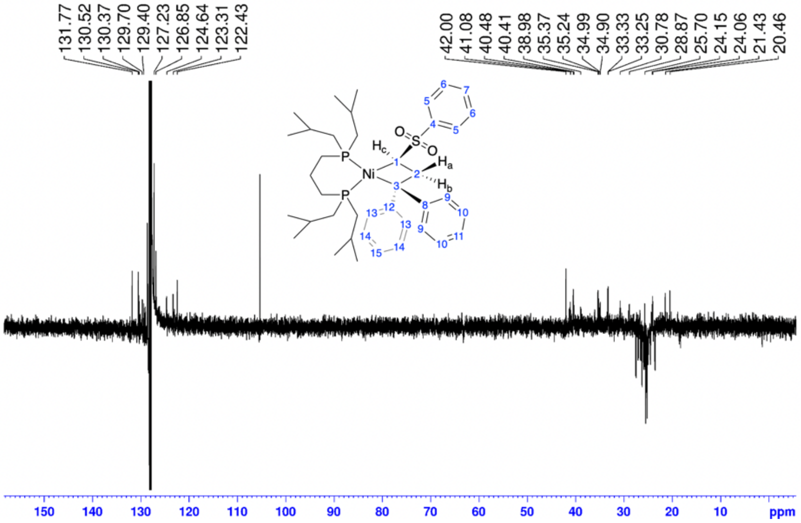
131.77
130.52
130.37
129.70
129.40
127.23
126.85
124.64
123.31
122.43
42.00
41.08
40.48
40.41
38.98
35.37
35.24
34.99
34.90
33.33
33.25
30.78
28.87
25.70
24.15
24.06
21.43
20.46
ppm

(f)

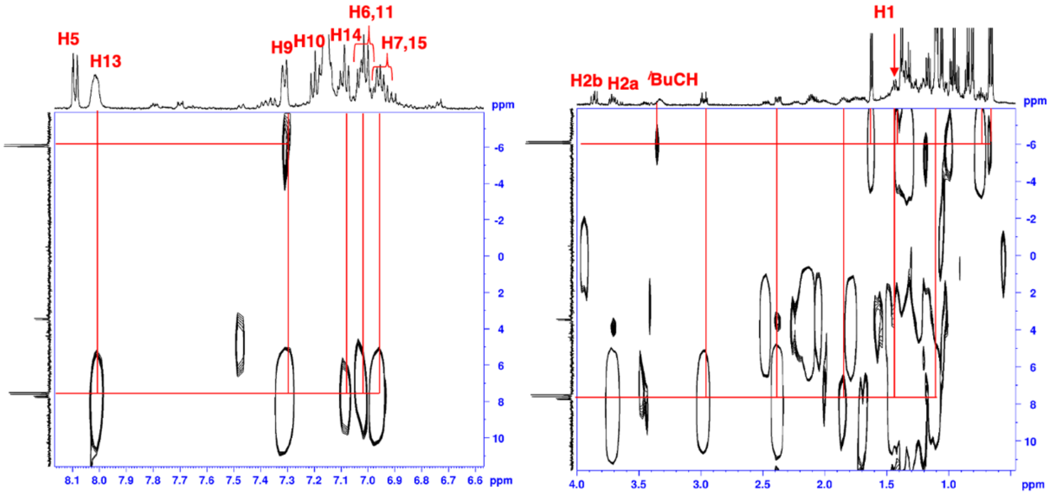
H5
H13
H9
H10
H14
H6,11
H7,15
H2b
H2a
^{i}BuCH
H1
ppm

(g)

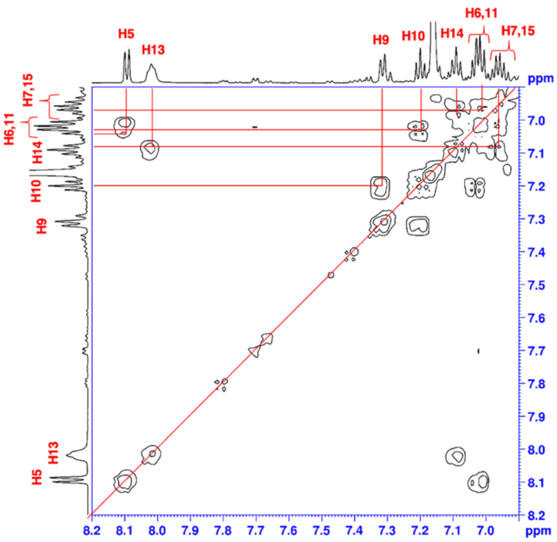

(h)

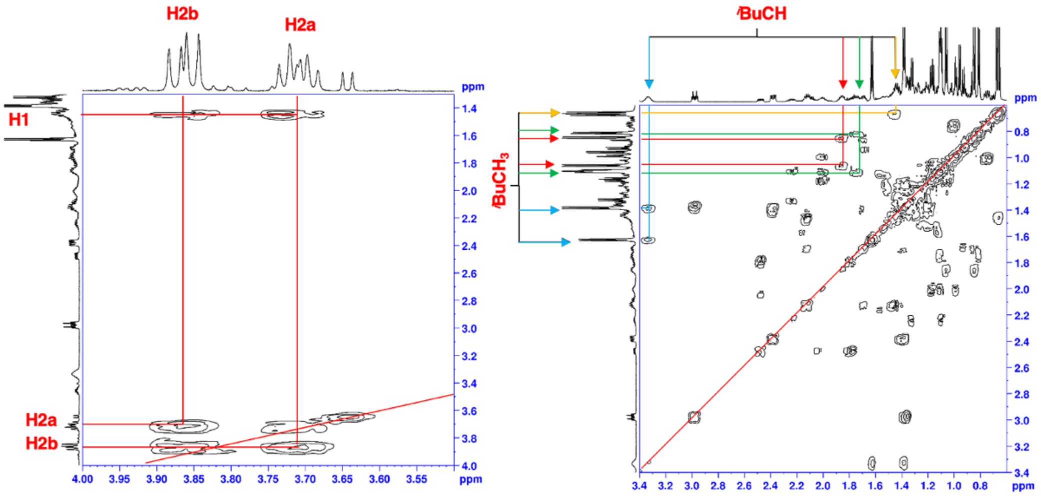

(i)

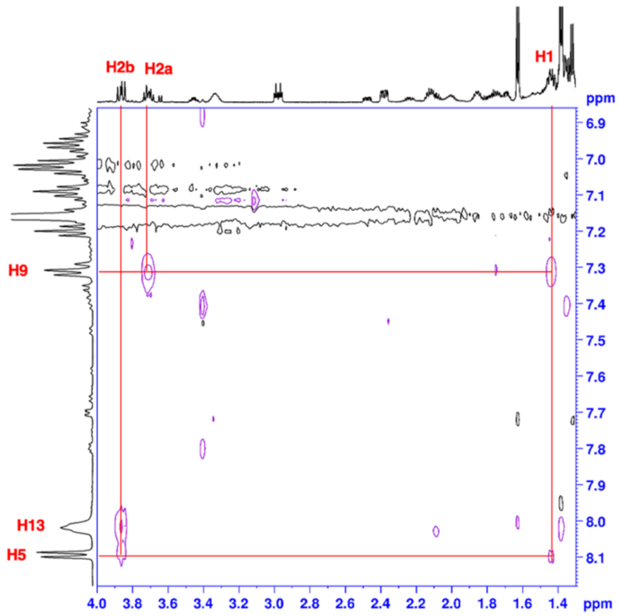

(j)

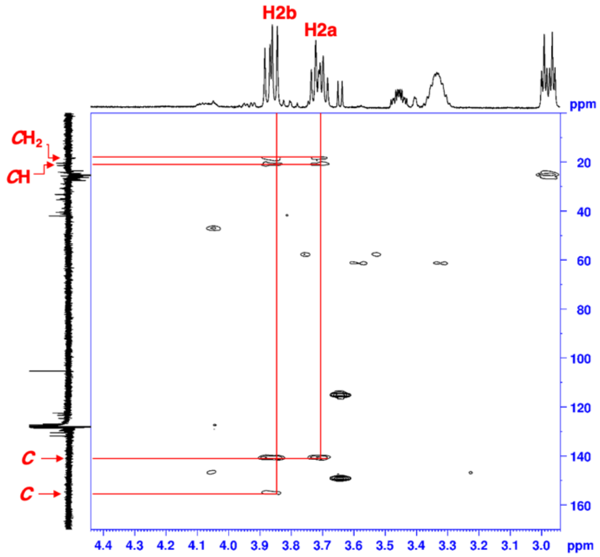

(k)

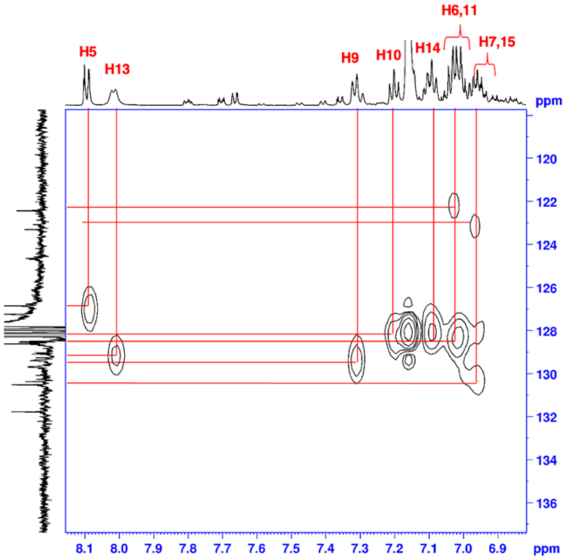

Figure S9. NMR spectra of **Ni-5c** in C_6D_6. (a) ^{1}H NMR (600 MHz: signals for **Ni-1c**, **Ni-7c** and **5** subtracted). (b) ^{1}H NMR (600 MHz). (c) ^{31}P{^{1}H} (202 MHz: signals for **Ni-1c** and **Ni-7c** subtracted). (d) ^{31}P{^{1}H} (202 MHz). (e) DEPT135 (151 MHz). (f) ^{1}H-^{31}P HMBC expansion (600 MHz, 202 MHz). (g) ^{1}H-^{1}H COSY expansion (600 MHz) h) ^{1}H-^{1}H COSY expansion (600 MHz). (i) ^{1}H-^{1}H NOESY expansion (600 MHz). (j) ^{1}H-^{13}C HMBC expansion (600 MHz, 151 MHz) k) ^{1}H-^{13}C HSQC expansion (600 MHz, 151 MHz).

S2.4 Characterization of Ni(PP)(Olefin) Adducts

S2.4.1 Ni(dibpp)(PVS), Ni-7c

(a)

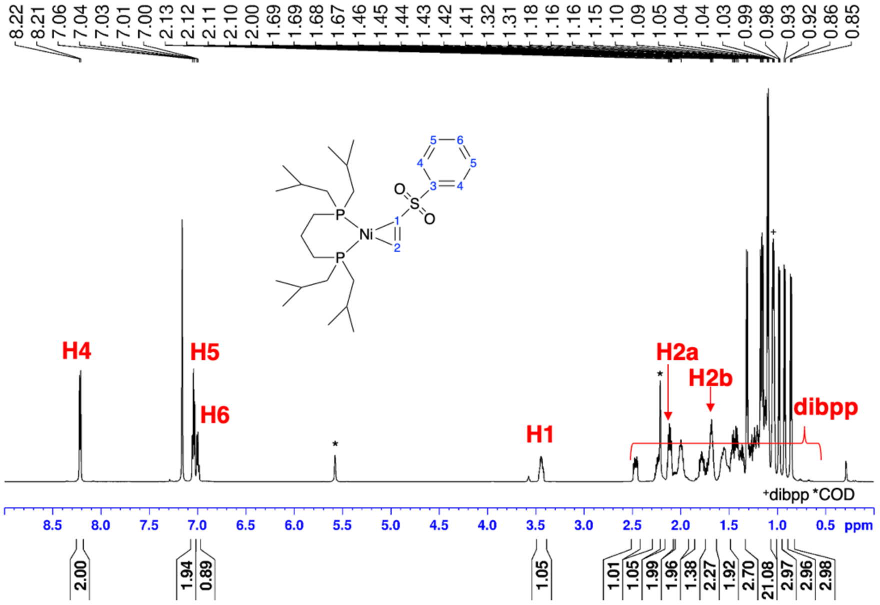

(b)

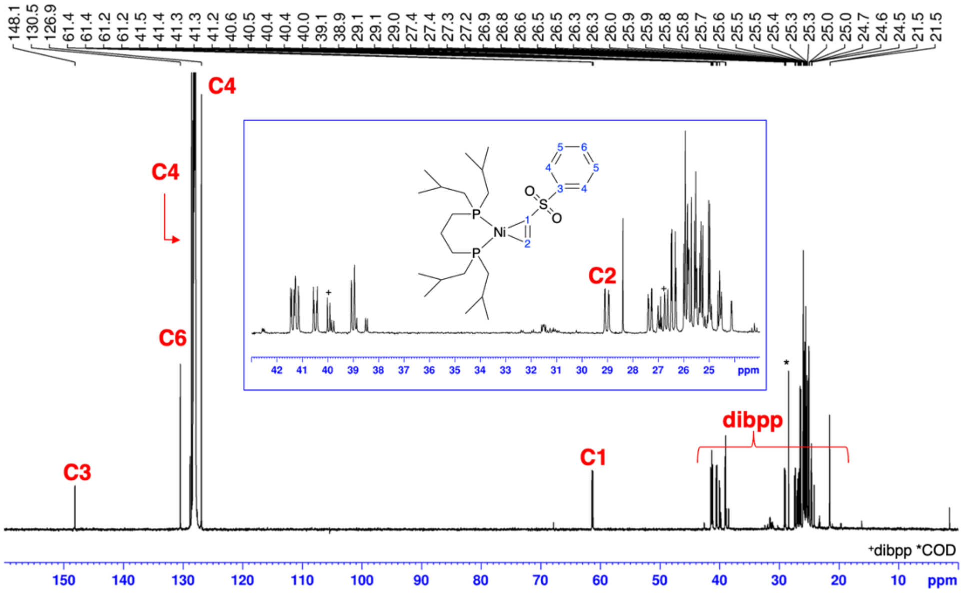

148.1 130.5 126.9 61.4 61.4 61.2 61.2 41.5 41.4 41.3 41.3 41.2 40.6 40.5 40.4 40.4 40.0 39.1 38.9 29.1 29.1 29.0 27.4 27.4 27.3 27.2 26.9 26.8 26.6 26.5 26.5 26.3 26.3 26.0 25.9 25.9 25.8 25.8 25.7 25.6 25.5 25.5 25.4 25.3 25.3 25.0 25.0 24.7 24.6 24.5 21.5 21.5
C4
C4
C6
C3
C1
C2
dibpp
P
P
Ni
S
O
O
1
2
3
4
4
5
5
6
+dibpp *COD
ppm

(c)

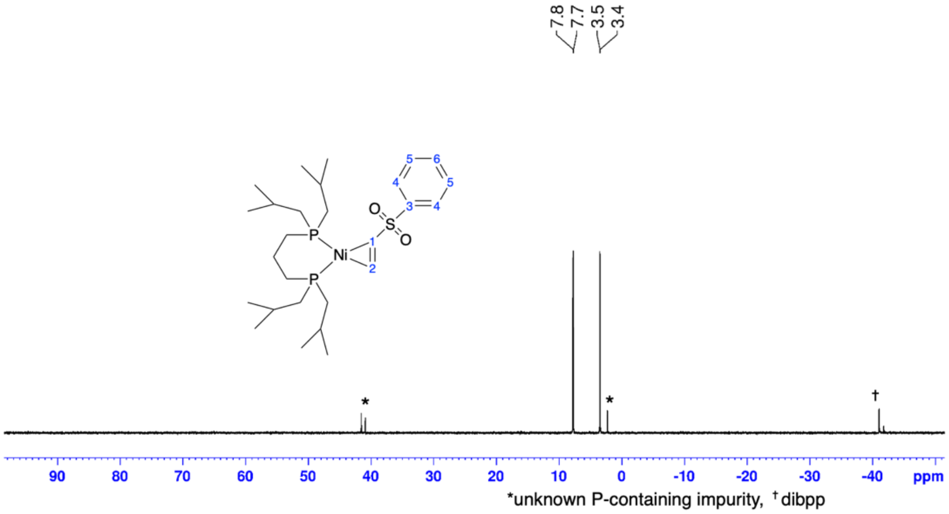

7.8 7.7 3.5 3.4
P
P
Ni
S
O
O
ppm
*unknown P-containing impurity, †dibpp

(d)

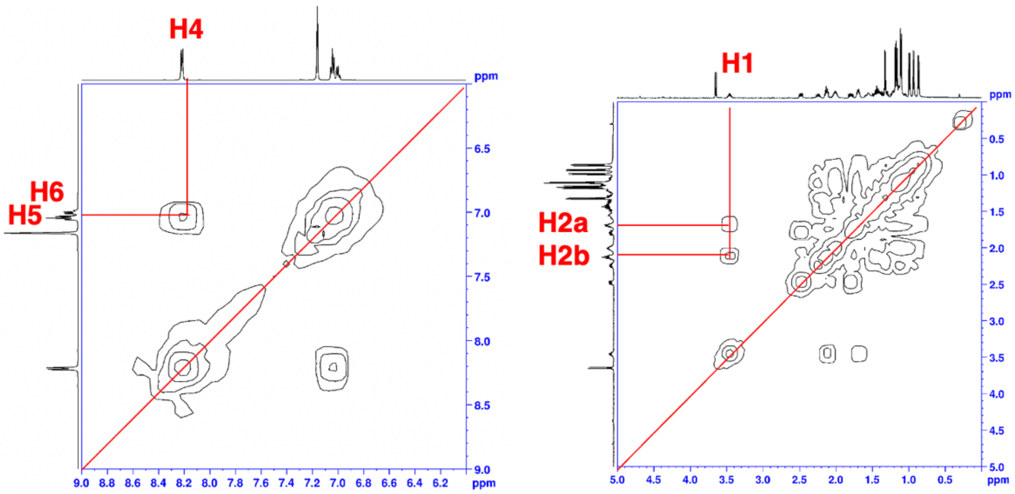

H4
H6
H5
H1
H2a
H2b
ppm

(e)

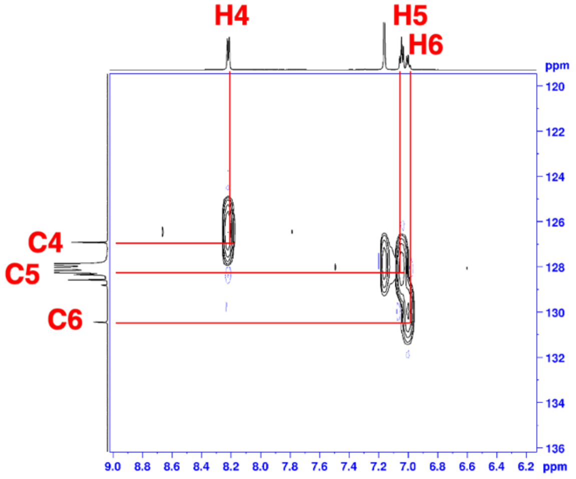

(f)

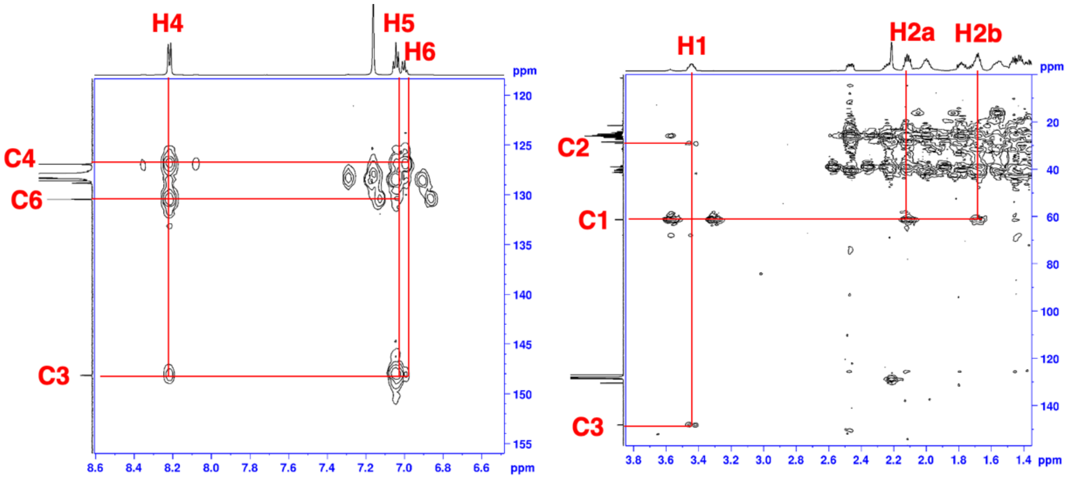

Figure S10. NMR Characterization of **Ni-7c** (C_6D_6). (a) ^{1}H NMR (600 MHz). (b) ^{13}C{^{1}H} (151 MHz). (c) ^{31}P{^{1}H} (202 MHz). (d) ^{1}H-^{1}H COSY expansion (600 MHz). (e) ^{1}H-^{13}C HSQC expansion (600 MHz, 151 MHz). (f) ^{1}H-^{13}C HMBC expansion (600 MHz, 151 MHz).

S2.4.2 Ni(dibpp)(styrene) Ni-8c

(a)

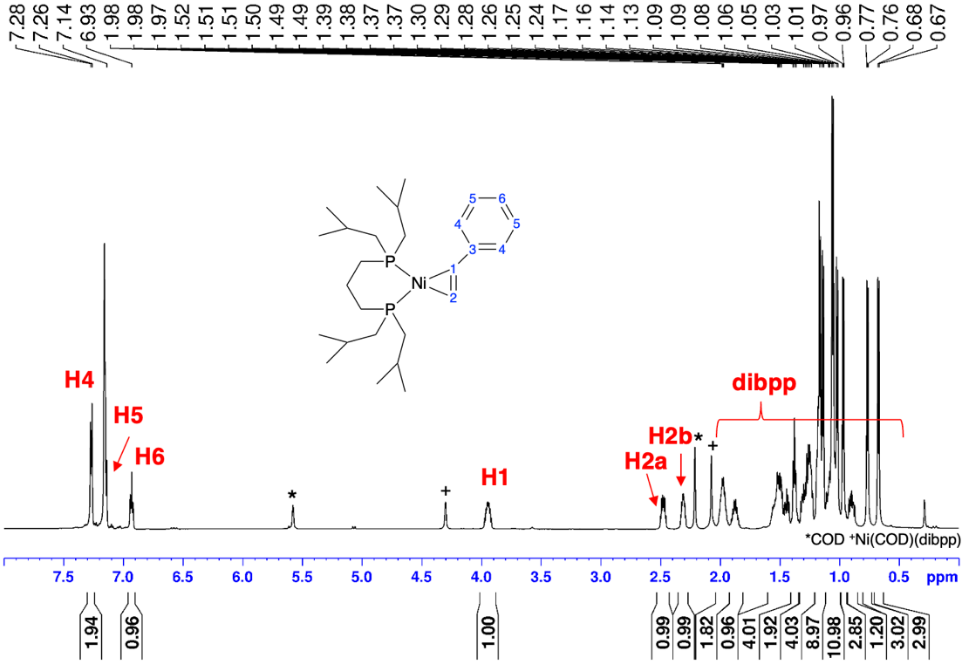

(b)

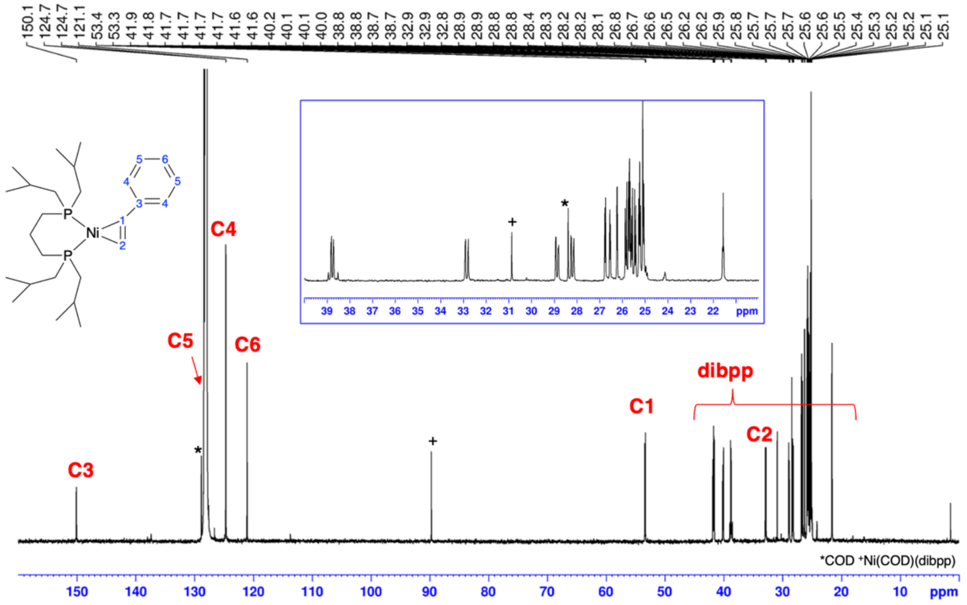

(c)

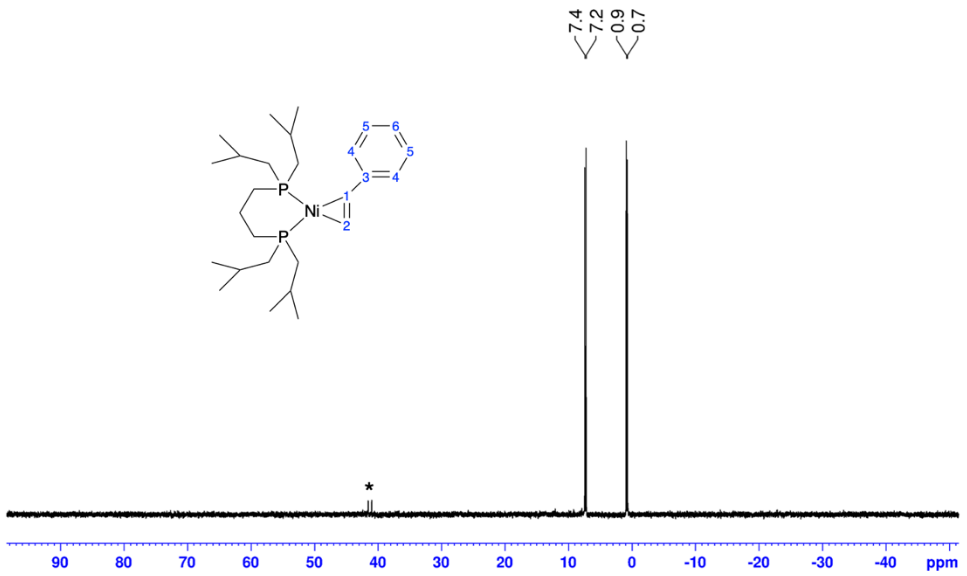

7.4
7.2
0.9
0.7
Ni
P
P
1
2
3
4
5
6
5
4
*
90
80
70
60
50
40
30
20
10
0
-10
-20
-30
-40
ppm

(d)

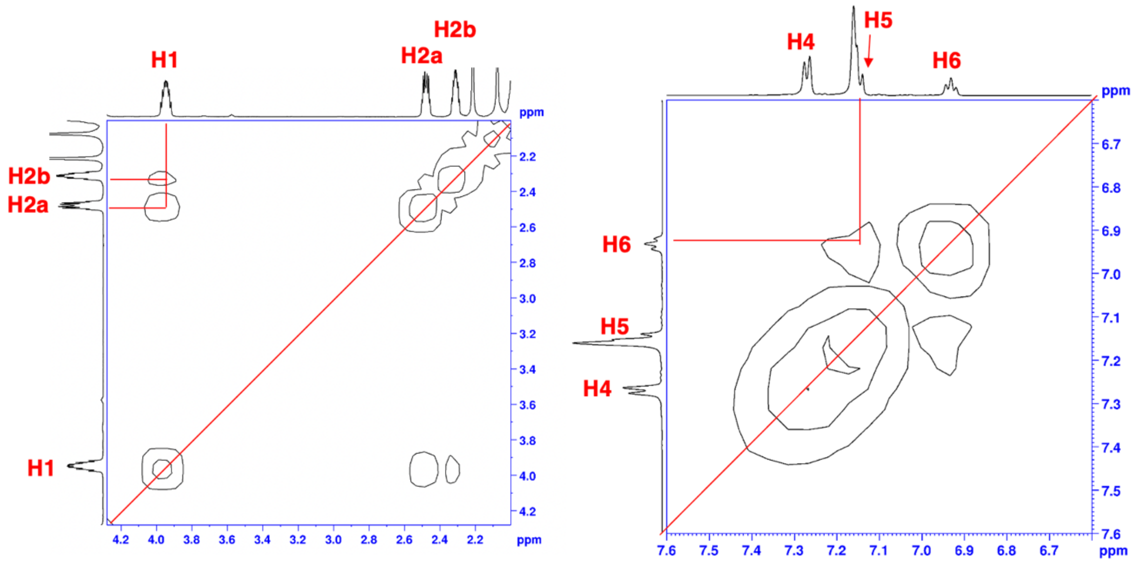

H1
H2a
H2b
H2b
H2a
H1
ppm
H4
H5
H6
H6
H5
H4
ppm

(e)

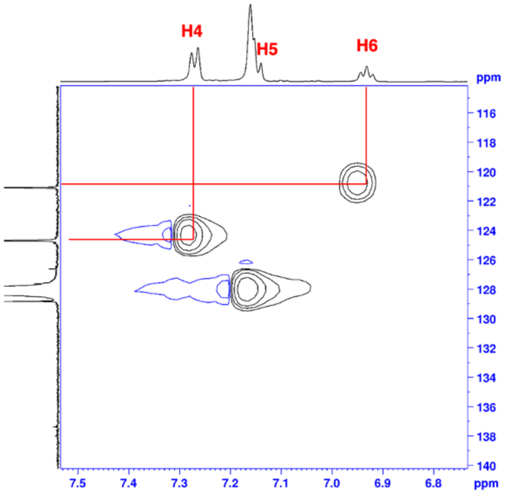

(f)

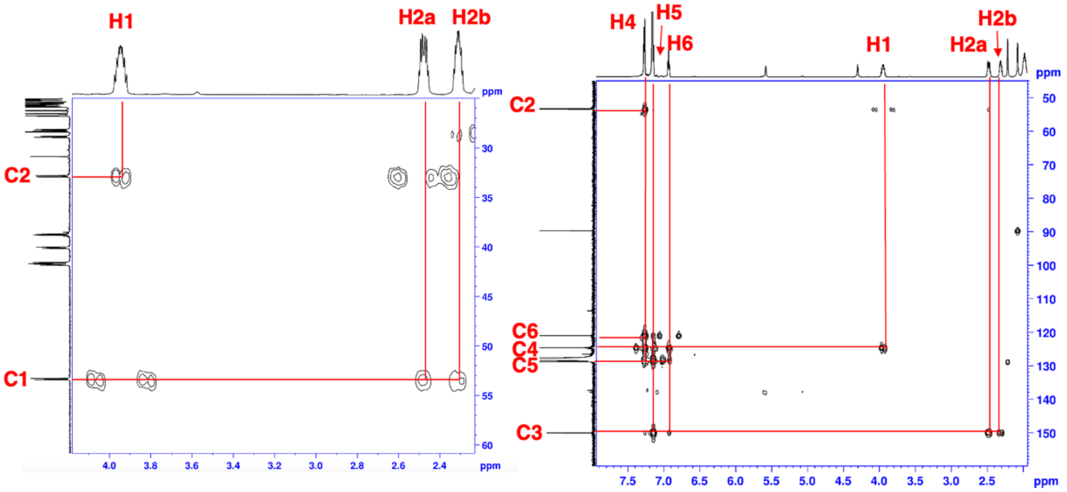

Figure S11. NMR spectra of **Ni-8c** (C_6D_6). (a) ^{1}H NMR (600 MHz). (b) ^{13}C{^{1}H} (151 MHz). (c) ^{31}P{^{1}H} (202 MHz). (d) ^{1}H-^{1}H COSY expansion (600 MHz). (e) ^{1}H-^{13}C HSQC expansion (600 MHz, 151 MHz). (f) ^{1}H-^{13}C HMBC expansion (600 MHz, 151 MHz).

S2.5 Decomposition in Solution of Carbene Complexes Ni-1

(a)

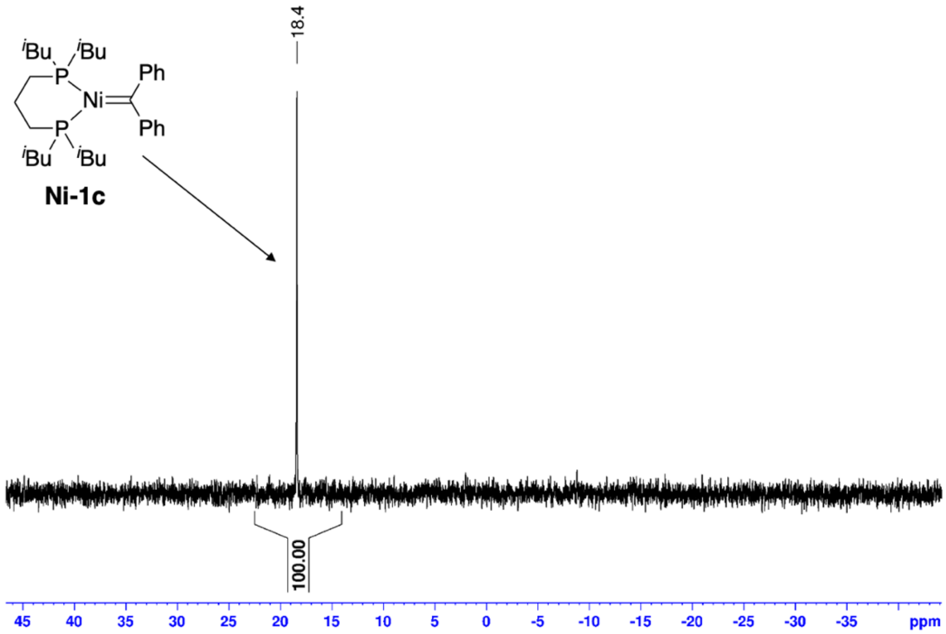

(b)

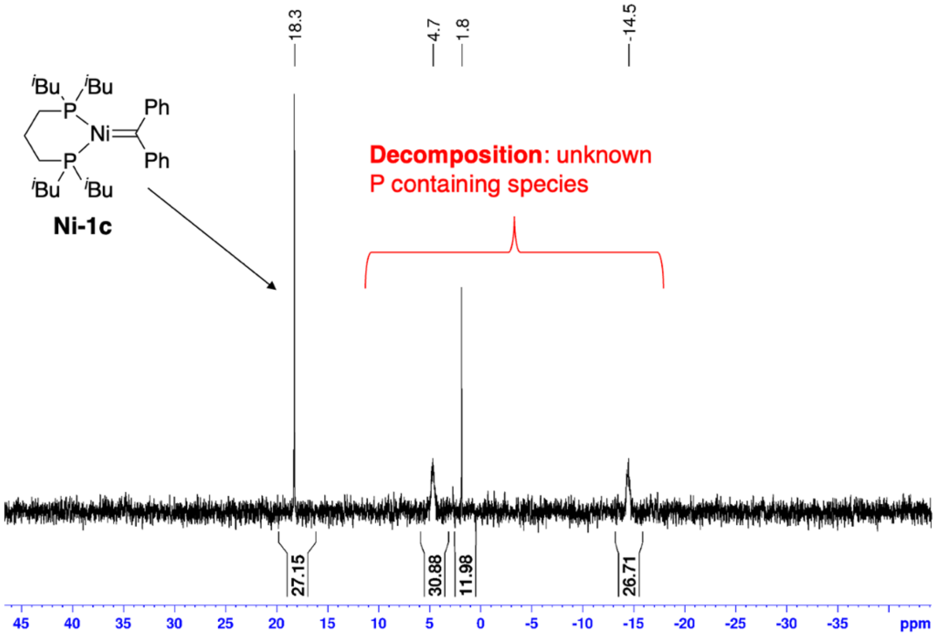

Figure S12. $^{31}P\{^1H\}$NMR (121 MHz, C_6D_6) spectrum showing decomposition of **Ni-1c** on workup.

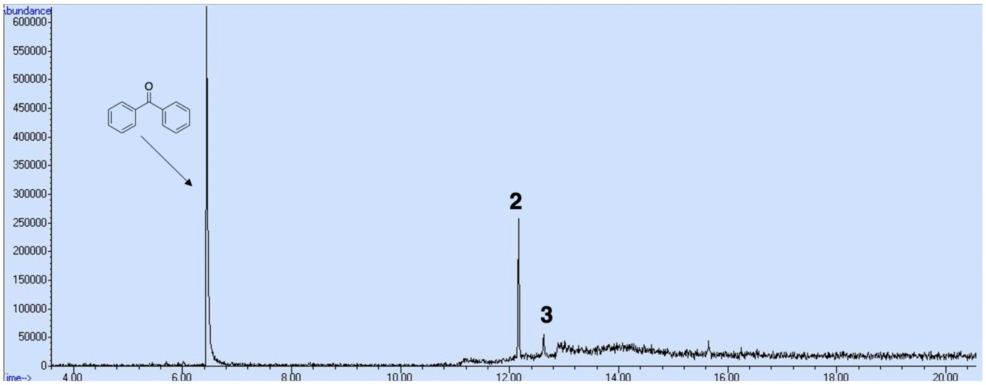

Figure S13. GC/MS chromatogram of concentrated sample of **Ni-1c** showing formation of tetraphenylethylene **2**.

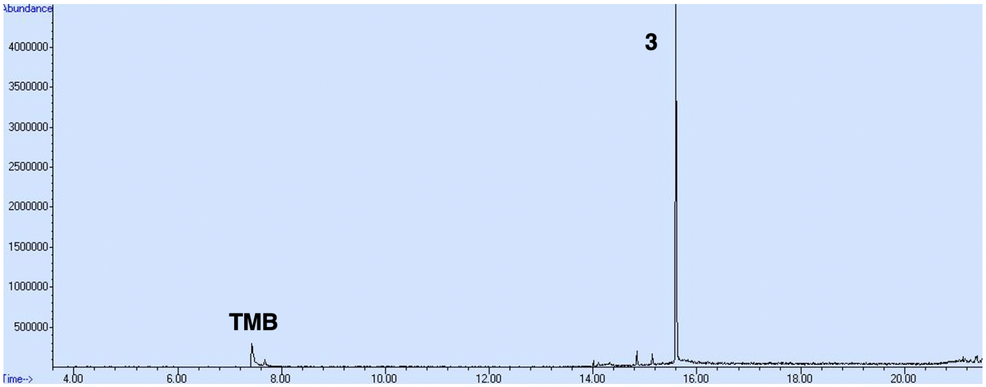

Figure S14. GC/MS chromatogram for reaction of **Ni-1c** with HCl.

(a)

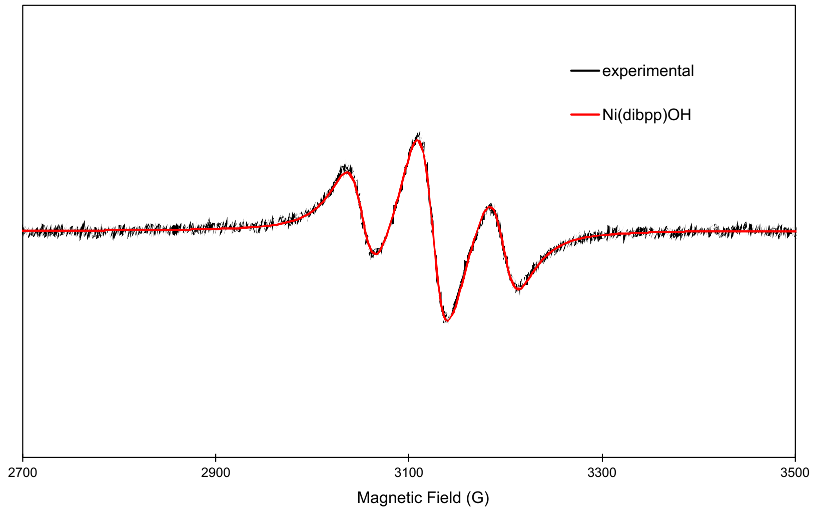

(b)

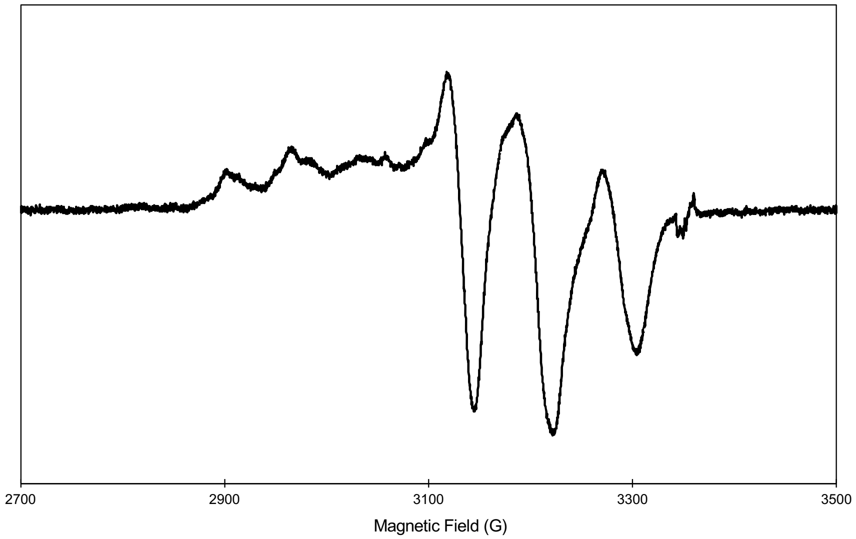

Figure S15. (a) EPR spectrum (9.386 MHz, RT) for reaction of **Ni-1c** with H_2O. Spectrum is consistent with Ni(dibpp)OH (**Ni-4c**) modeled using (g = 2.15, a = 73 G). (b) EPR spectrum (9.391 MHz, 90 K) of reaction for **Ni-1c.**

S2.6 Mass Spectra

S2.6.1 MALDI Mass Spectra of Ni-2b and Ni-1b

(a)

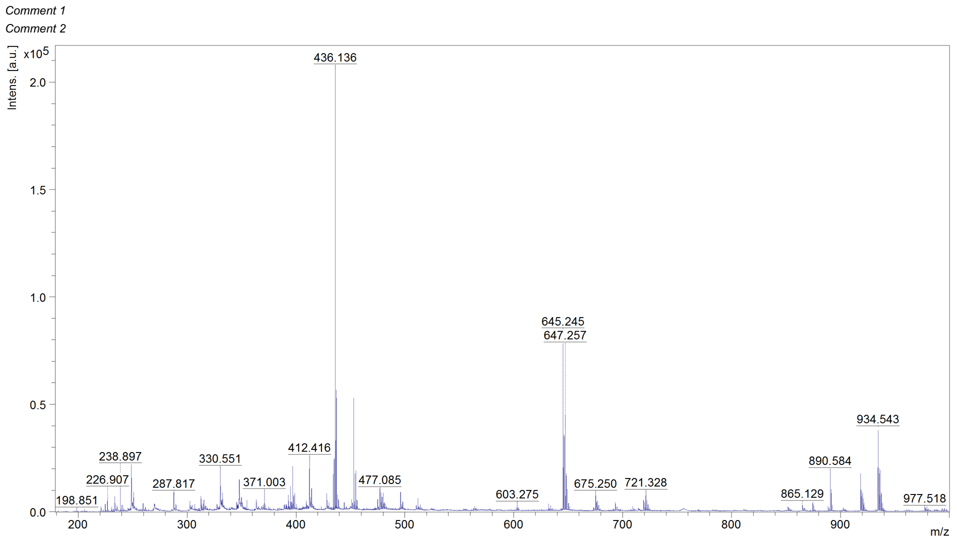

(b)

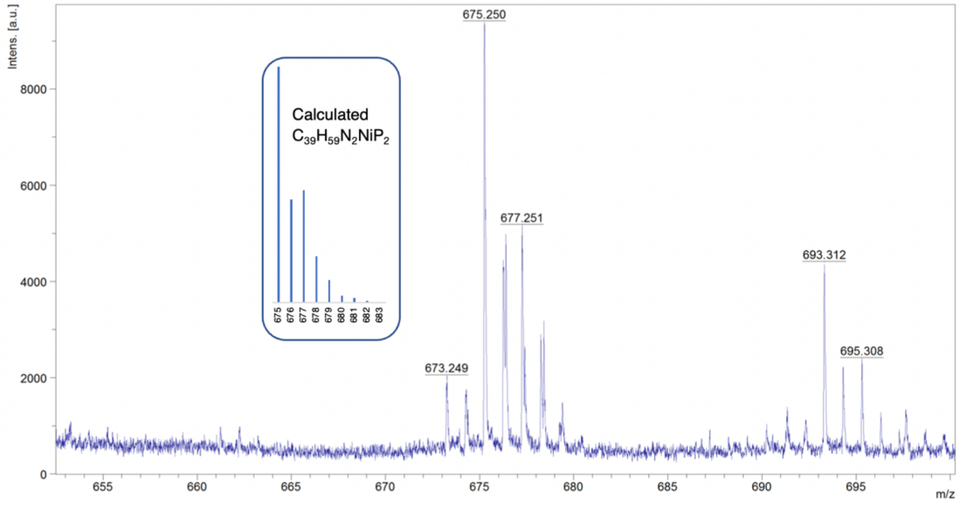

Figure S16. MALDI-TOF MS of **Ni-2b**. (a) Full spectrum. (b) Expansion of the [M^++H] signal with simulated isotope pattern (inset) shown for comparison.

(a)

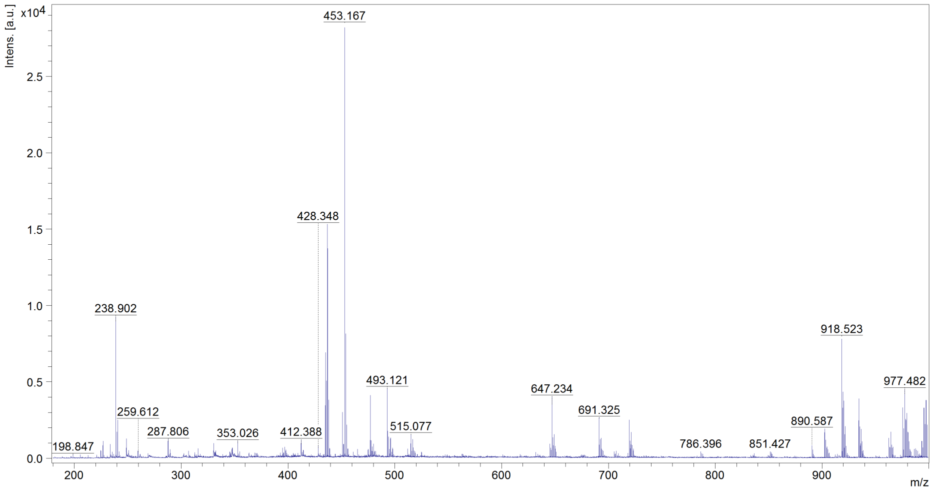

(b)

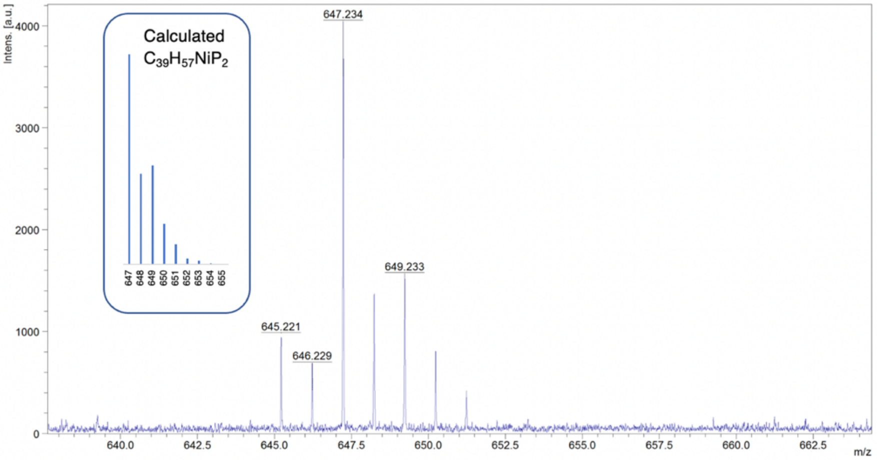

Figure S17. MALDI-TOF MS of **Ni-1b**. (a) Full spectrum. (b) Expansion of the $[M^{+}+H]$ signal, with simulated isotope pattern (inset) shown for comparison

S2.6.2 Mass Spectra of Ni-2c (LIFDI) and Ni-1c (MALDI)

(a)

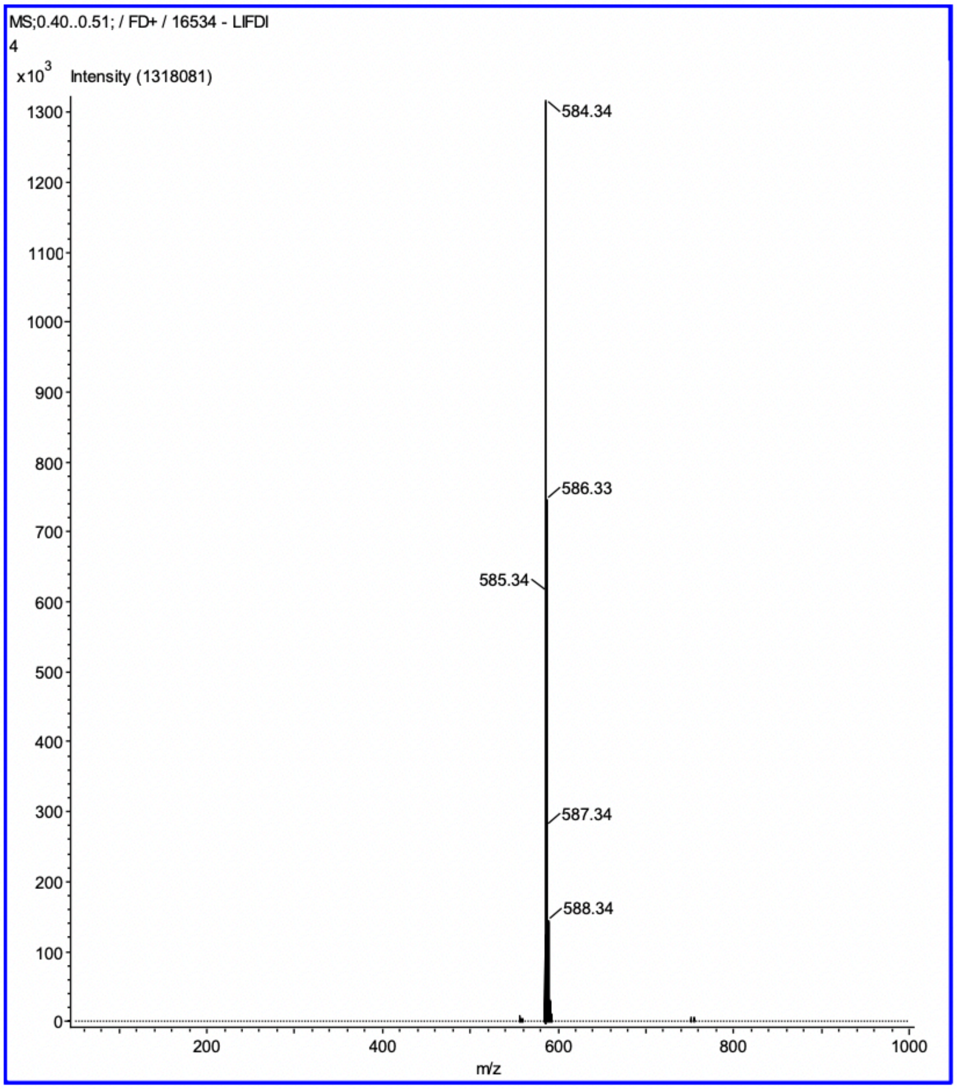

(b)

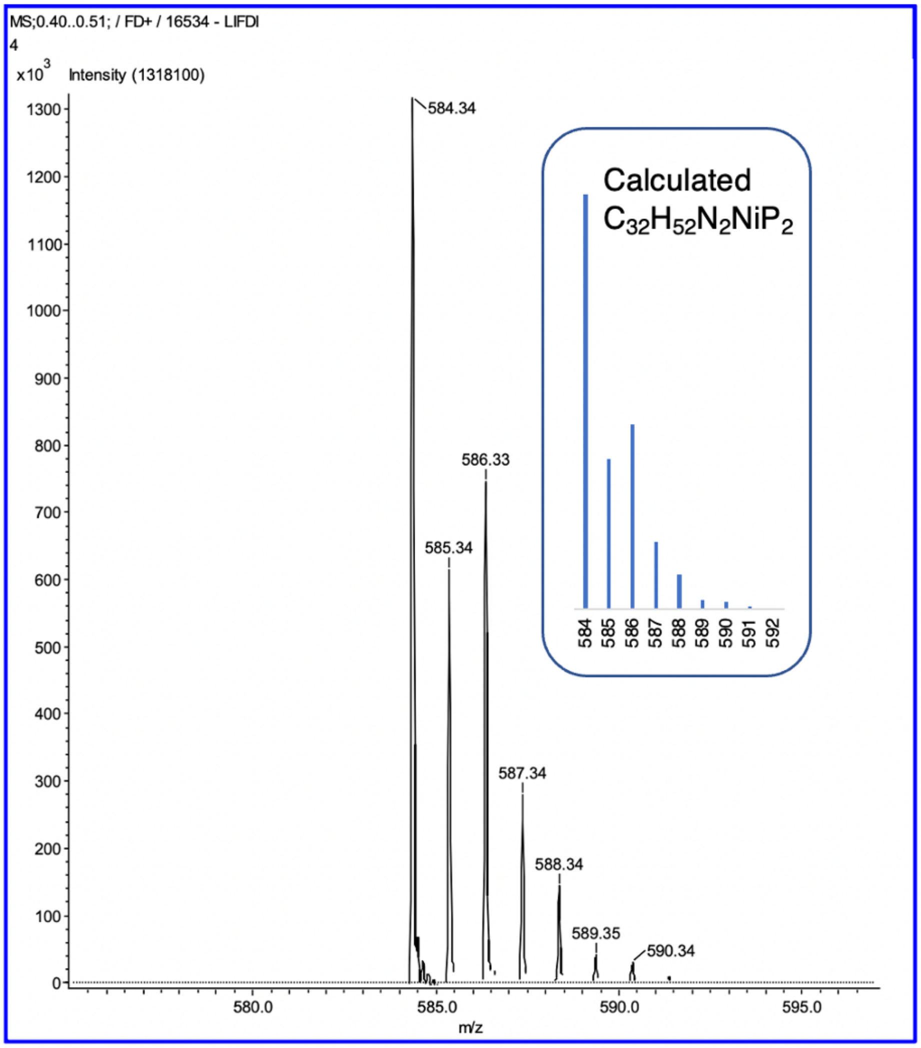

Figure S18. LIFDI-MS of **Ni-2c**. **(**a) Full spectrum. (b) Expansion of [M^+] signal, with simulated isotope pattern (inset) shown for comparison.

(a)

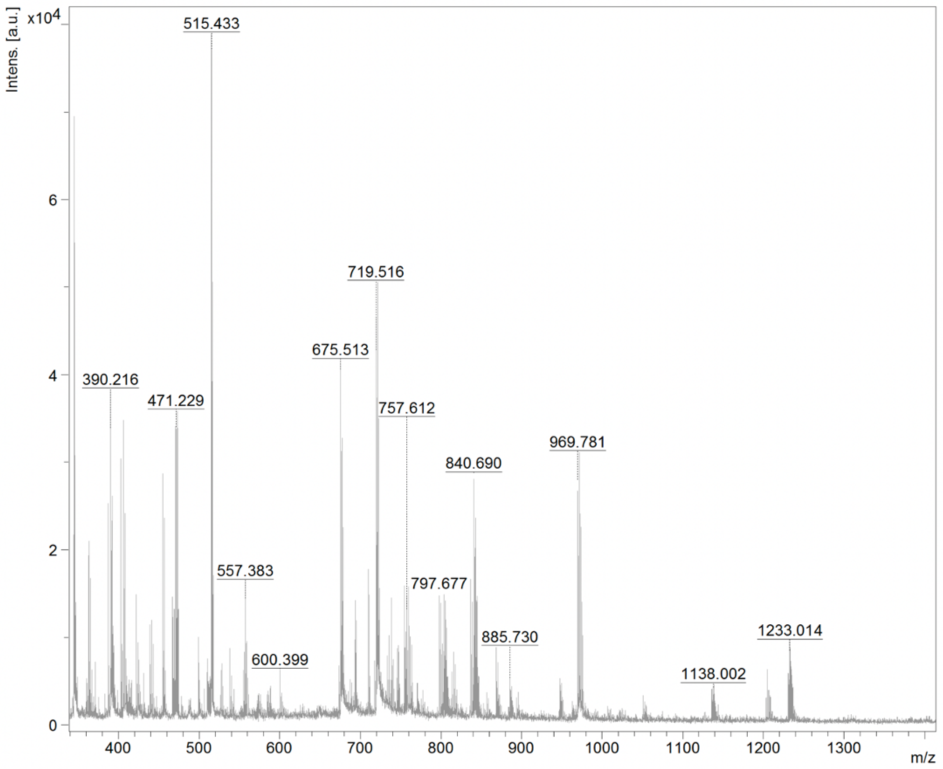

(b)

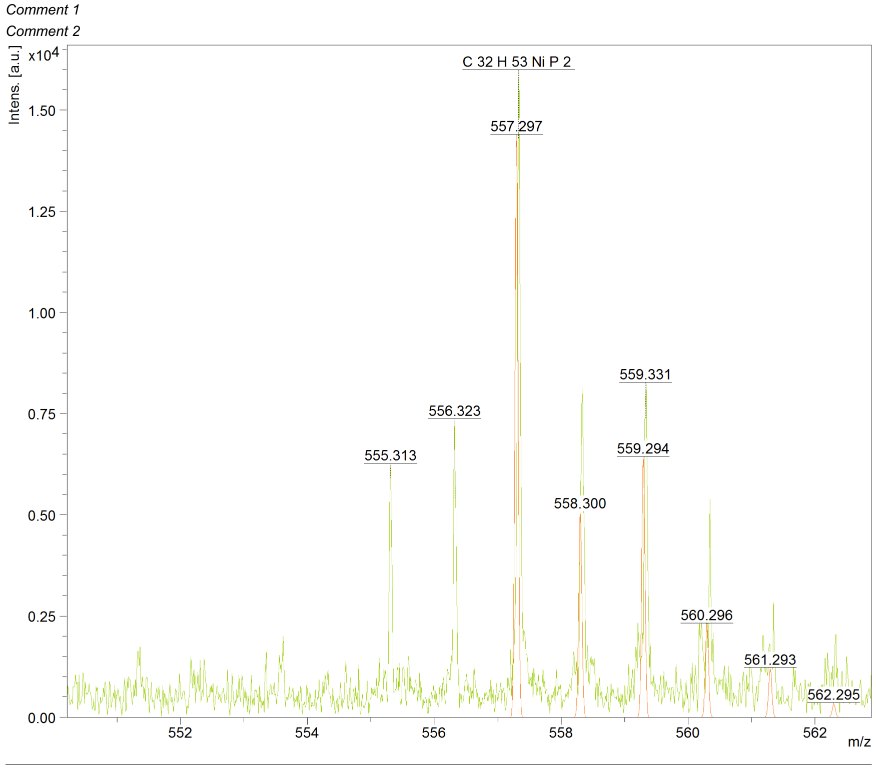

Figure S19. MALDI-MS of **Ni-1c. (a)** Full MALDI spectrum. (b) Expansion of [M^{+}+H] signal, with simulated pattern in inset.

S2.6.3 LIFDI Mass Spectrum of Ni-1d

(a)

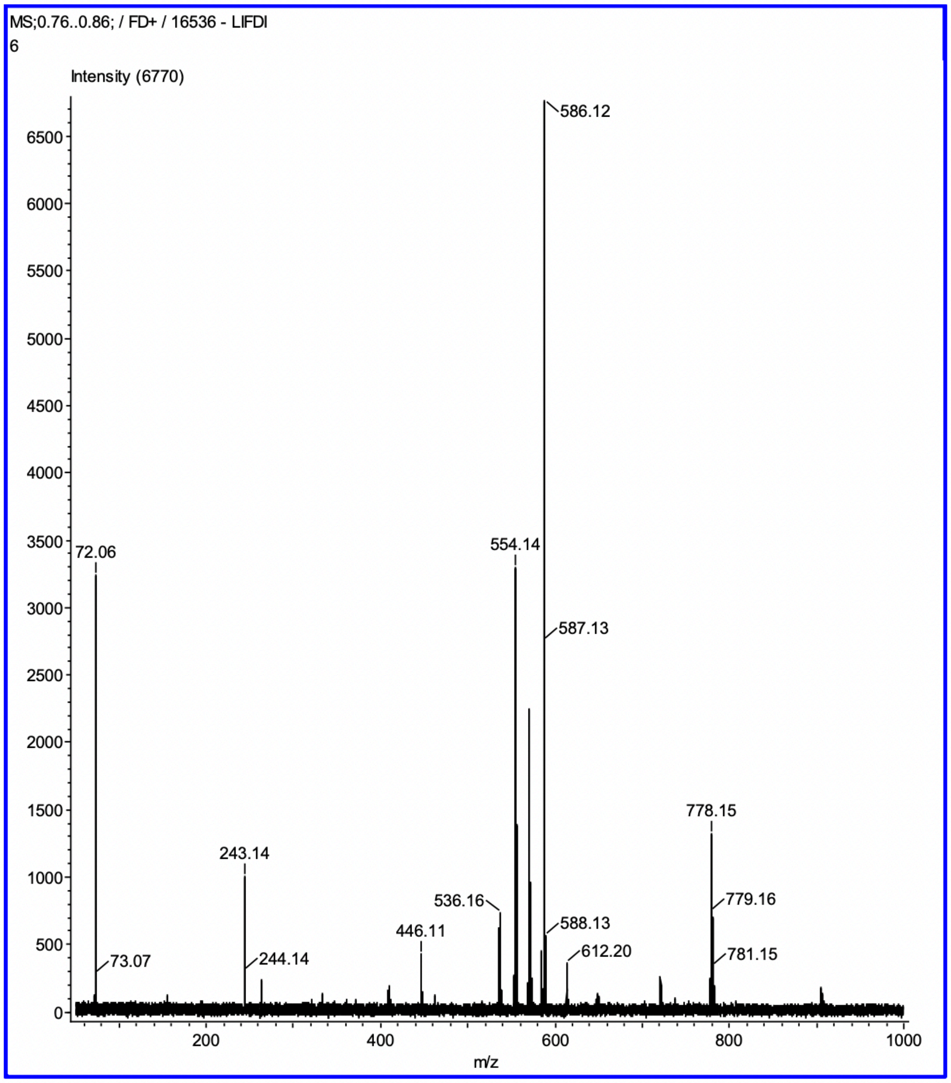

(b)

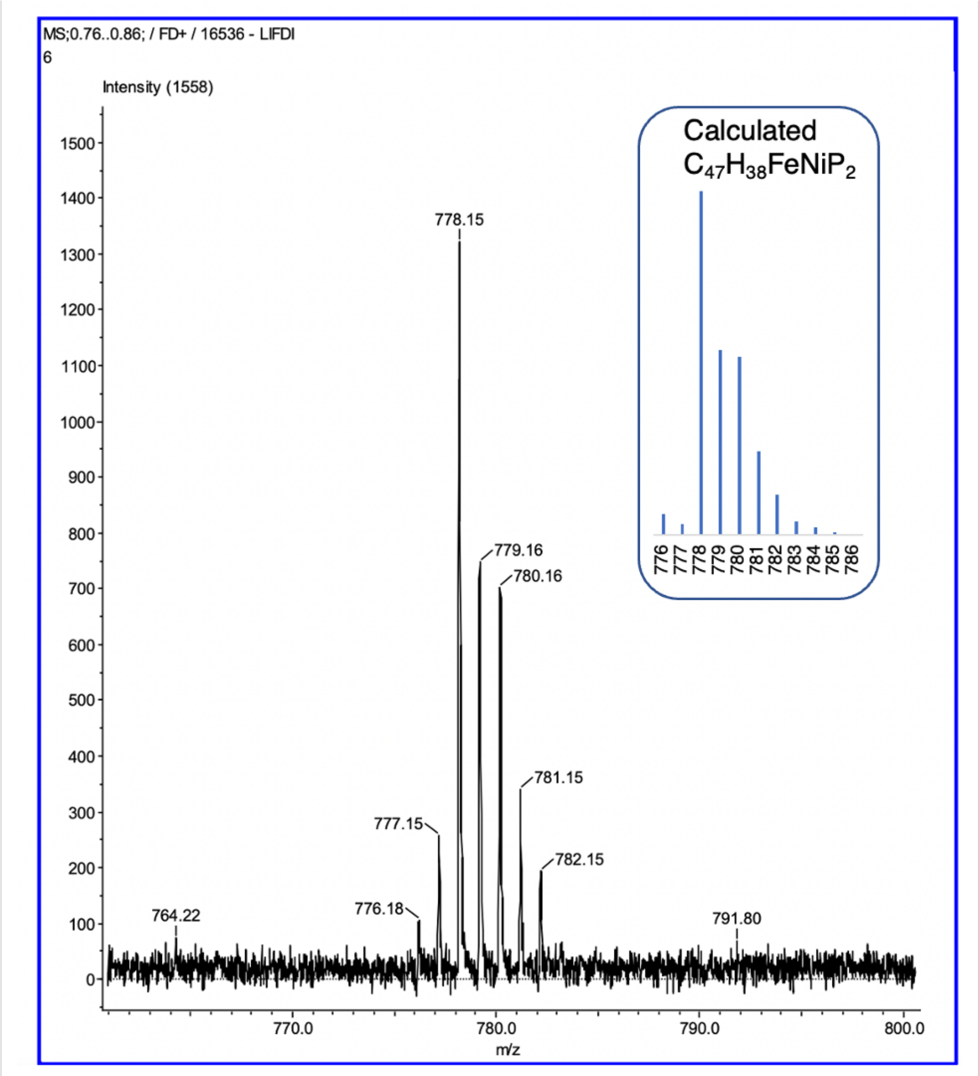

Figure S20. LIFDI-MS of **Ni-1d**, formed via loss of N_2 from **Ni-2d** under the ionization conditions. No **Ni-2d** is observed. (a) Full LIFDI spectrum. (b) Expansion of [M^+] signal for **Ni-1d**, with simulated isotope pattern (inset) shown for comparison.

S2.6.4 LIFDI Mass Spectrum of Ni-1e from Ni-2e

(a)

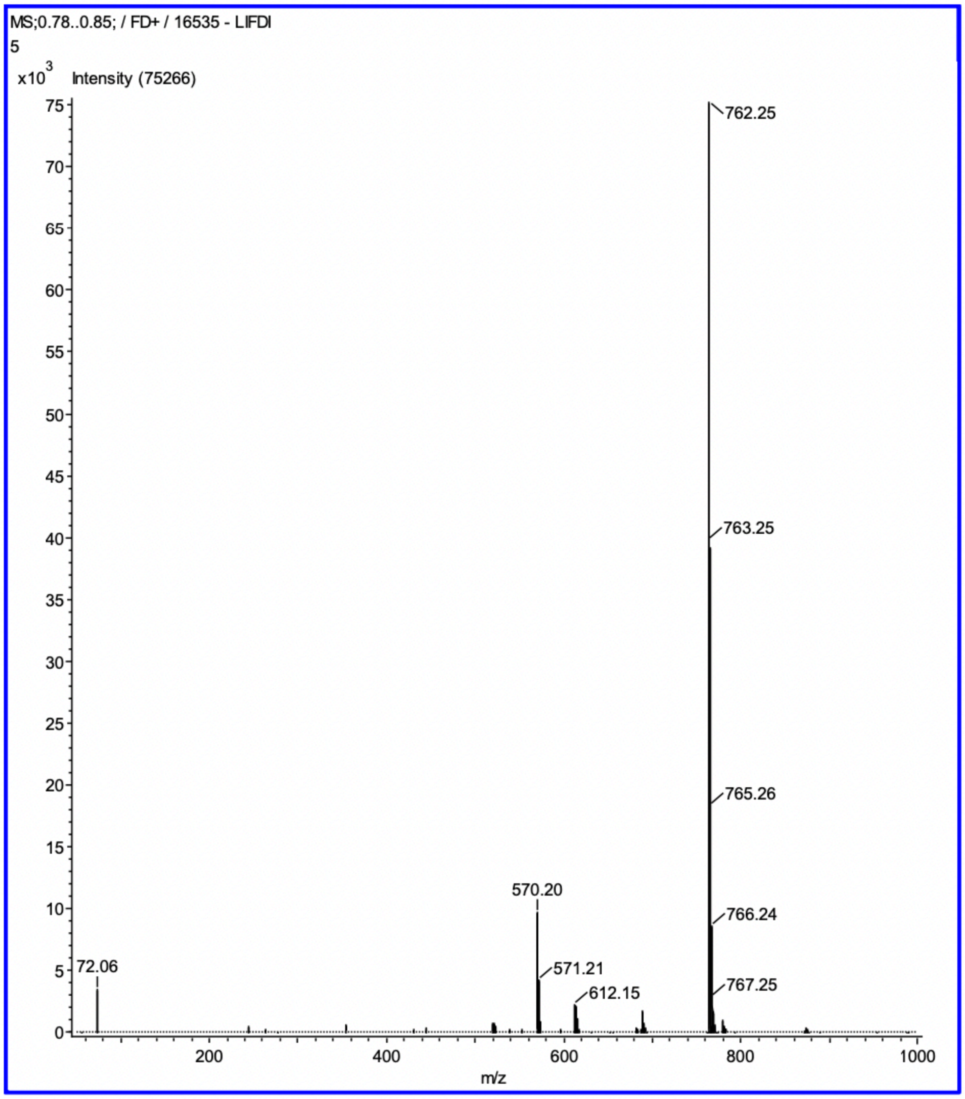

(b)

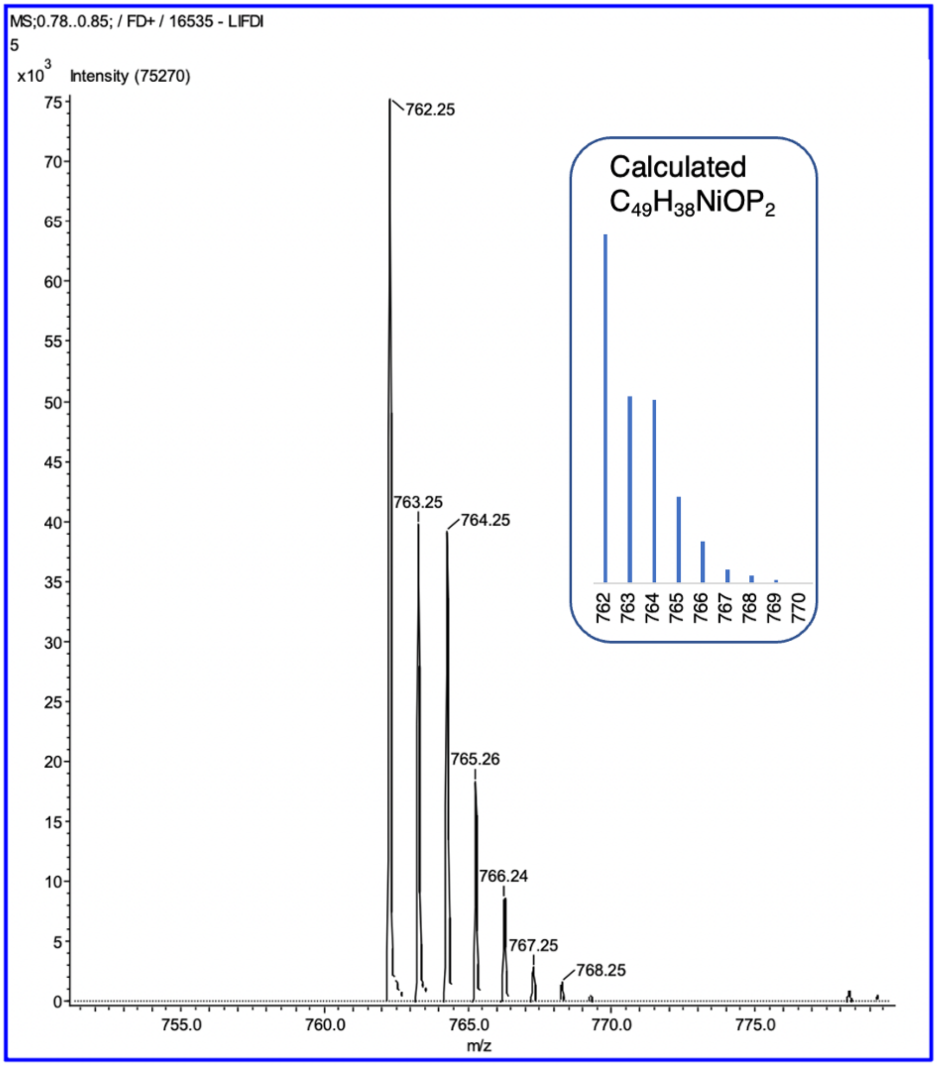

Figure S21. LIFDI-MS of **Ni-1e**, formed via loss of N_2 from **Ni-2d** under the ionization conditions. No **Ni-2e** is observed. (a) Full LIFDI spectrum. (b) Expansion of [M^+] signal for **Ni-1e**, with simulated isotope pattern (inset) shown for comparison.

S2.6.5 Mass Spectrum Showing Isotopically-Labelled Product on D_2O Hydrolysis of Ni-1c

(a)

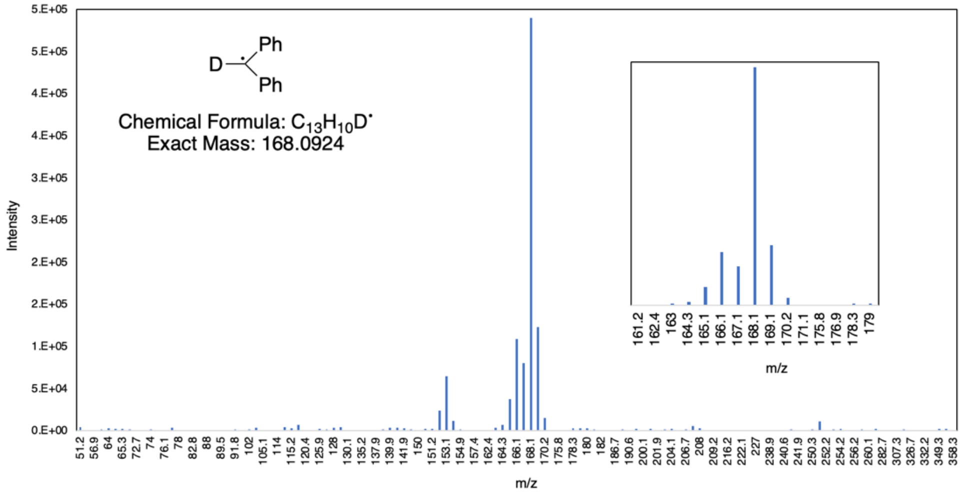

(b)

Compound Information

Name / ID:
Formula: C13H10D
m/z: 168.0924
Adduct: [M]+
Adduct m/z: 168.0918

Results

Deuteration: **85.65 %**
Deuteration Ratio Spectra
D0: 14.35 %
D1: 85.65 %

Spectra

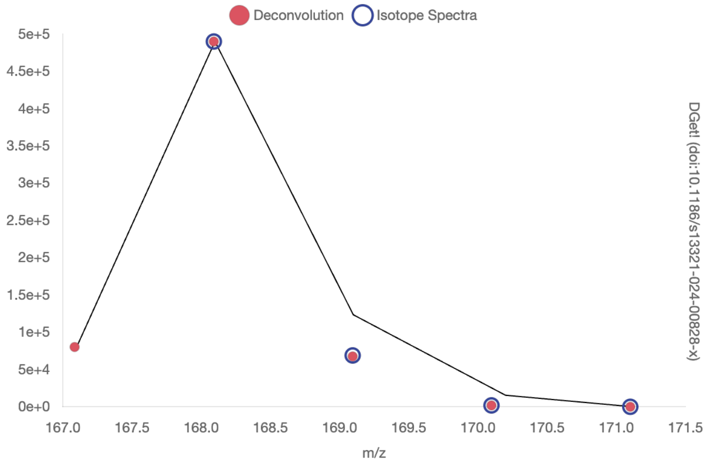

Report generated using the DGet (0.26) web application (0.27.3).

Figure S22. (a) Electron-impact MS of tetraphenylethane-d_2 peak in GC/MS trace for the **Ni-1c** + D_2O experiment. (b) Calculated % deuteration: DGet! Report.

S2.7 Analysis of Reactions of Ni-1 Complexes with Styrene

(a)

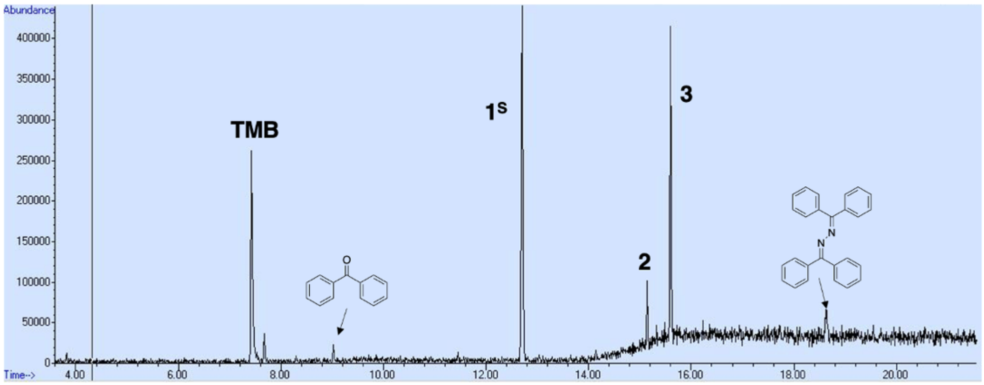

(b)

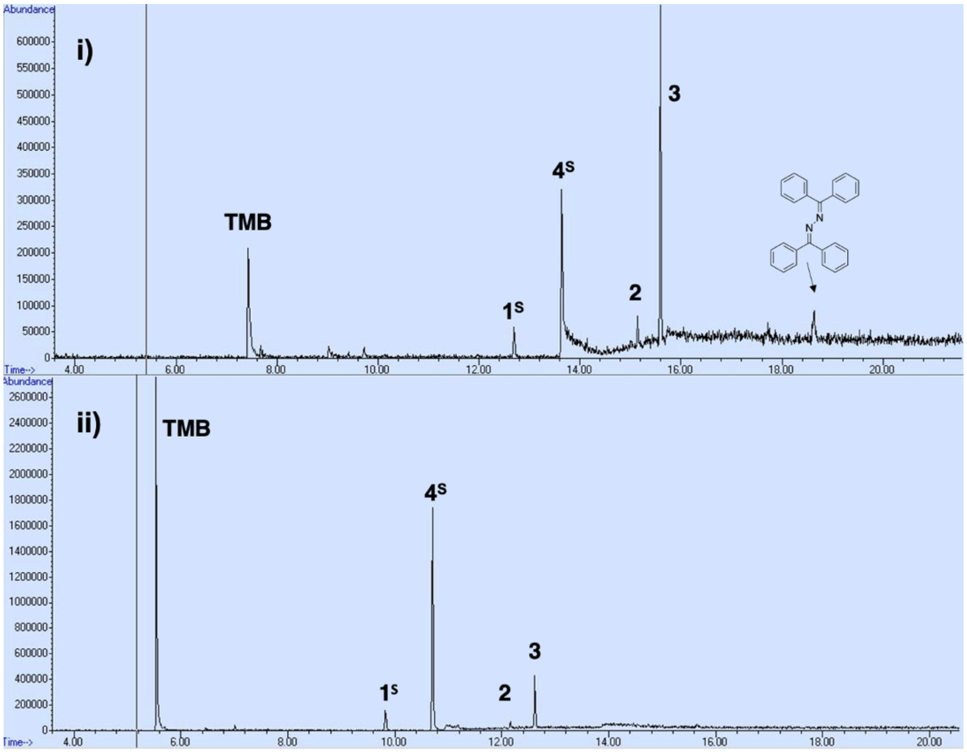

(c)

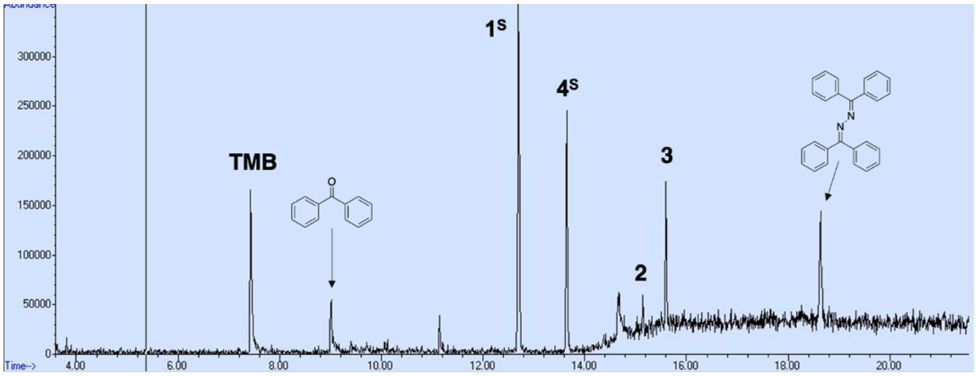

(d)

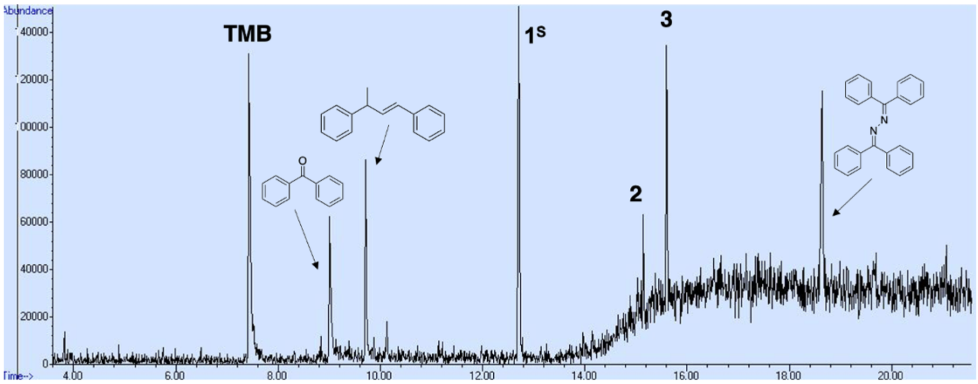

Figure S23. GC/MS chromatograms of the reactions of **Ni-1b-e** with styrene at 24 h. (a) **Ni-1b**. (b) **Ni-1c**: from precursor synthesized via i) standard procedure; ii) with drying step. (c) **Ni-1d**. (d) **Ni-1e**.

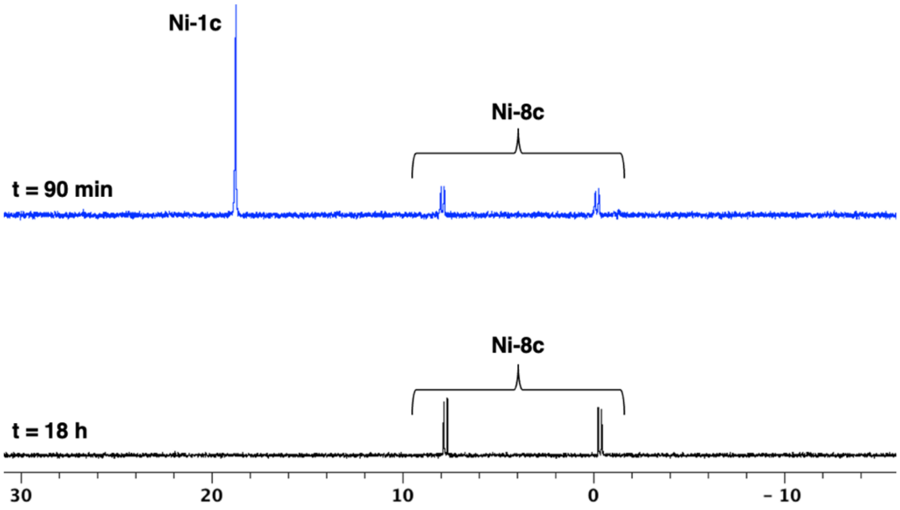

Figure S24. $^{31}P\{^{1}H\}$NMR (202 MHz, C_7H_8,–50 °C) spectrum for the reaction of **Ni-1c** with styrene at 90 min and 18 h, indicating the absence of an observable intermediate.

S3. X-ray Crystallographic Analysis

S3.1 X-ray Analysis of NiCB Complex Ni-5c

Table S5. Crystallographic Experimental Details

A. Crystal Data	
formula	$C_{40}H_{60}NiO_2P_2S$
formula weight	725.59
crystal colour and habit[a]	red plate
crystal dimensions (mm)	0.31 × 0.15 × 0.08
crystal system	monoclinic
space group	$P2_1/n$ (an alternate setting of $P2_1/c$ [No. 14])
unit cell parameters[b]	
a (Å)	11.2144(9)
b (Å)	18.6941(14)
c (Å)	18.4280(15)
β (deg)	95.335(3)
V (Å^3)	3846.6(5)
Z	4
ρ_{calcd} (g cm^{-3})	1.253
μ (mm^{-1})	0.674
B. Data Collection and Refinement Conditions	
diffractometer	Bruker D8/APEX II CCD[c]
radiation (λ [Å])	graphite-monochromated Mo Kα (0.71073)
temperature (°C)	–173
scan type	ω and ϕ scans (0.5°) (10 s exposures)
data collection 2θ limit (deg)	55.17
total data collected	54145 ($-14 \le h \le 14$, $-24 \le k \le 24$, $-23 \le l \le 23$)
independent reflections	8846 (R_{int} = 0.0882)
number of observed reflections (*NO*)	6590 [$F_o^2 \ge 2\sigma(F_o^2)$]
structure solution method	intrinsic phasing (*SHELXT-2018/2*[d])
refinement method	full-matrix least-squares on F^2 (*SHELXL–2019/1*[e])
absorption correction method	multi-scan (*SADABS*)
range of transmission factors	0.7456–0.6781
data/restraints/parameters	8846 / 0 / 423
goodness-of-fit (S)[f] [all data]	1.125
final R indices[g]	
R_1 [$F_o^2 \ge 2\sigma(F_o^2)$]	0.0534
wR_2 [all data]	0.1104
largest difference peak and hole	0.702 and –0.476 e Å^{-3}

[a]Obtained by recrystallization from diethyl ether. [b]Obtained by least-squares refinement of 9779 reflections with 4.44° < 2θ < 55.08°. [c]Programs for diffractometer operation, data collection, data reduction and absorption correction were those supplied by Bruker. Data collected at the University of Ottawa. [d]Sheldrick, G. M. *Acta Crystallogr.* **2015**, *A71*, 3–8. (*SHELXT-2018/2*). [e]Sheldrick, G. M. *Acta Crystallogr.* **2015**, *C71*, 3–8. (*SHELXL-2019/1*). [f]$S = [\Sigma w(F_o^2 - F_c^2)^2/(n - p)]^{1/2}$ (n = number of data; p = number of parameters varied; $w = [\sigma^2(F_o^2) + 7.4828P]^{-1}$ where $P = [\mathrm{Max}(F_o^2, 0) + 2F_c^2]/3$). [g]$R_1 = \Sigma||F_o| - |F_c||/\Sigma|F_o|$; $wR_2 = [\Sigma w(F_o^2 - F_c^2)^2/\Sigma w(F_o^4)]^{1/2}$.

Table S6. Atomic Coordinates and Equivalent Isotropic Displacement Parameters

Atom	*x*	*y*	*z*	U_{eq}, Å^2
Ni1	0.28260(3)	0.56553(2)	0.27488(2)	0.01370(9)*
S1	0.21076(6)	0.40797(4)	0.31908(4)	0.01488(15)*
P1	0.28938(7)	0.52024(4)	0.16369(4)	0.01581(16)*
P2	0.43977(6)	0.63575(4)	0.26316(4)	0.01525(16)*
O1	0.17364(18)	0.36686(10)	0.25398(10)	0.0186(4)*
O2	0.33610(17)	0.40484(11)	0.34561(11)	0.0198(4)*
C1	0.1635(2)	0.49593(15)	0.30853(15)	0.0165(6)*
C2	0.1636(3)	0.53355(15)	0.38200(15)	0.0174(6)*
C3	0.2129(2)	0.60663(14)	0.36387(15)	0.0147(6)*
C4	0.3388(3)	0.58515(15)	0.09714(16)	0.0218(6)*
C5	0.3248(3)	0.66276(16)	0.12137(16)	0.0225(7)*
C6	0.4252(3)	0.68774(16)	0.17742(16)	0.0210(6)*
C11	0.1298(2)	0.37464(14)	0.39069(15)	0.0154(6)*
C12	0.1913(3)	0.34800(15)	0.45355(16)	0.0202(6)*
C13	0.1259(3)	0.32470(16)	0.50942(17)	0.0236(7)*
C14	0.0029(3)	0.32848(16)	0.50260(17)	0.0233(7)*
C15	-0.0578(3)	0.35588(17)	0.43996(17)	0.0252(7)*
C16	0.0056(3)	0.37940(16)	0.38391(16)	0.0213(6)*
C21	0.1229(2)	0.65305(15)	0.32078(15)	0.0155(6)*
C22	0.1554(2)	0.71676(15)	0.28678(15)	0.0160(6)*
C23	0.0729(3)	0.75760(16)	0.24451(16)	0.0206(6)*
C24	-0.0461(3)	0.73725(16)	0.23410(16)	0.0210(6)*
C25	-0.0808(3)	0.67528(16)	0.26692(16)	0.0213(6)*
C26	0.0013(2)	0.63385(16)	0.30889(15)	0.0181(6)*
C31	0.2903(2)	0.63891(15)	0.42581(15)	0.0154(6)*
C32	0.3781(3)	0.59656(16)	0.46457(16)	0.0198(6)*
C33	0.4493(3)	0.62279(17)	0.52394(16)	0.0238(7)*
C34	0.4359(3)	0.69236(18)	0.54705(16)	0.0264(7)*
C35	0.3500(3)	0.73506(17)	0.50984(17)	0.0255(7)*
C36	0.2783(3)	0.70893(16)	0.45031(16)	0.0191(6)*
C41	0.1397(2)	0.49239(16)	0.12499(16)	0.0197(6)*
C42	0.0378(3)	0.54641(17)	0.13146(17)	0.0254(7)*
C43	-0.0803(3)	0.5074(2)	0.13679(19)	0.0350(8)*
C44	0.0242(3)	0.59924(19)	0.06829(19)	0.0351(8)*
C51	0.3815(3)	0.43934(15)	0.15363(15)	0.0185(6)*
C52	0.4388(3)	0.42431(16)	0.08267(16)	0.0225(7)*
C53	0.3470(3)	0.41295(19)	0.01755(17)	0.0309(8)*
C54	0.5198(3)	0.35899(18)	0.09300(18)	0.0296(7)*
C61	0.5720(2)	0.57879(15)	0.25304(16)	0.0182(6)*
C62	0.6002(3)	0.52043(16)	0.31085(17)	0.0212(6)*

Table S6. Atomic Coordinates and Displacement Parameters (continued)

Atom	x	y	z	U_{eq}, Å^2
C63	0.6863(3)	0.46539(18)	0.2831(2)	0.0340(8)*
C64	0.6556(3)	0.55010(17)	0.38328(17)	0.0260(7)*
C71	0.4894(2)	0.70409(15)	0.33131(16)	0.0181(6)*
C72	0.6145(3)	0.73908(15)	0.33565(16)	0.0193(6)*
C73	0.6369(3)	0.78384(17)	0.26912(18)	0.0270(7)*
C74	0.6279(3)	0.78649(17)	0.40365(18)	0.0263(7)*

Anisotropically-refined atoms are marked with an asterisk (*). The form of the anisotropic displacement parameter is: $\exp[-2\pi^2(h^2a^{*2}U_{11} + k^2b^{*2}U_{22} + l^2c^{*2}U_{33} + 2klb^*c^*U_{23} + 2hla^*c^*U_{13} + 2hka^*b^*U_{12})]$.

Table S7. Selected Interatomic Distances (Å)

Atom1	Atom2	Distance	Atom1	Atom2	Distance
Ni1	P1	2.2248(8)	C15	C16	1.379(4)
Ni1	P2	2.2243(8)	C21	C22	1.410(4)
Ni1	C1	2.004(3)	C21	C26	1.407(4)
Ni1	C3	2.032(3)	C22	C23	1.383(4)
S1	O1	1.453(2)	C23	C24	1.383(4)
S1	O2	1.446(2)	C24	C25	1.380(4)
S1	C1	1.733(3)	C25	C26	1.384(4)
S1	C11	1.782(3)	C31	C32	1.406(4)
P1	C4	1.847(3)	C31	C36	1.395(4)
P1	C41	1.837(3)	C32	C33	1.383(4)
P1	C51	1.851(3)	C33	C34	1.381(5)
P2	C6	1.849(3)	C34	C35	1.383(5)
P2	C61	1.849(3)	C35	C36	1.387(4)
P2	C71	1.841(3)	C41	C42	1.538(4)
C1	C2	1.526(4)	C42	C43	1.522(5)
C2	C3	1.522(4)	C42	C44	1.523(4)
C3	C21	1.501(4)	C51	C52	1.536(4)
C3	C31	1.496(4)	C52	C53	1.521(4)
C4	C5	1.530(4)	C52	C54	1.523(4)
C5	C6	1.529(4)	C61	C62	1.537(4)
C11	C12	1.384(4)	C62	C63	1.531(4)
C11	C16	1.390(4)	C62	C64	1.523(4)
C12	C13	1.389(4)	C71	C72	1.544(4)
C13	C14	1.375(4)	C72	C73	1.524(4)
C14	C15	1.383(4)	C72	C74	1.531(4)

Table S8. Selected Interatomic Angles (deg)

Atom1	Atom2	Atom3	Angle	Atom1	Atom2	Atom3	Angle
P1	Ni1	P2	92.28(3)	C12	C11	C16	121.0(3)
P1	Ni1	C1	96.82(8)	C11	C12	C13	118.5(3)
P1	Ni1	C3	159.35(8)	C12	C13	C14	120.7(3)
P2	Ni1	C1	165.99(9)	C13	C14	C15	120.5(3)
P2	Ni1	C3	102.89(8)	C14	C15	C16	119.7(3)
C1	Ni1	C3	71.23(11)	C11	C16	C15	119.6(3)
O1	S1	O2	116.92(12)	C3	C21	C22	122.3(2)
O1	S1	C1	110.38(13)	C3	C21	C26	121.9(3)
O1	S1	C11	107.45(12)	C22	C21	C26	115.7(3)
O2	S1	C1	110.72(13)	C21	C22	C23	122.0(3)
O2	S1	C11	106.35(13)	C22	C23	C24	120.8(3)
C1	S1	C11	104.08(13)	C23	C24	C25	118.6(3)
Ni1	P1	C4	113.56(10)	C24	C25	C26	120.9(3)
Ni1	P1	C41	110.92(10)	C21	C26	C25	122.0(3)
Ni1	P1	C51	118.07(10)	C3	C31	C32	119.4(3)
C4	P1	C41	104.36(14)	C3	C31	C36	124.0(3)
C4	P1	C51	105.42(13)	C32	C31	C36	116.6(3)
C41	P1	C51	103.18(13)	C31	C32	C33	121.9(3)
Ni1	P2	C6	112.66(10)	C32	C33	C34	120.5(3)
Ni1	P2	C61	108.66(9)	C33	C34	C35	118.8(3)
Ni1	P2	C71	122.36(10)	C34	C35	C36	121.0(3)
C6	P2	C61	102.98(14)	C31	C36	C35	121.3(3)
C6	P2	C71	102.43(14)	P1	C41	C42	116.3(2)
C61	P2	C71	105.91(13)	C41	C42	C43	110.3(3)
Ni1	C1	S1	116.44(15)	C41	C42	C44	112.9(3)
Ni1	C1	C2	91.71(17)	C43	C42	C44	109.5(3)
S1	C1	C2	111.3(2)	P1	C51	C52	121.1(2)
C1	C2	C3	100.9(2)	C51	C52	C53	113.0(3)
Ni1	C3	C2	90.71(17)	C51	C52	C54	109.4(2)
Ni1	C3	C21	94.70(17)	C53	C52	C54	109.8(3)
Ni1	C3	C31	121.92(19)	P2	C61	C62	117.0(2)
C2	C3	C21	113.3(2)	C61	C62	C63	110.0(3)
C2	C3	C31	113.0(2)	C61	C62	C64	112.9(2)
C21	C3	C31	119.0(2)	C63	C62	C64	108.6(3)
P1	C4	C5	112.6(2)	P2	C71	C72	123.0(2)
C4	C5	C6	113.4(3)	C71	C72	C73	114.1(2)
P2	C6	C5	114.7(2)	C71	C72	C74	107.9(2)
S1	C11	C12	119.8(2)	C73	C72	C74	109.2(2)
S1	C11	C16	119.1(2)				

Table S9. Torsional Angles (deg)

Atom1	Atom2	Atom3	Atom4	Angle	Atom1	Atom2	Atom3	Atom4	Angle
P2	Ni1	P1	C4	-34.20(12)	O1	S1	C11	C16	-61.8(3)
P2	Ni1	P1	C41	-151.35(11)	O2	S1	C11	C12	-4.3(3)
P2	Ni1	P1	C51	89.94(11)	O2	S1	C11	C16	172.3(2)
C1	Ni1	P1	C4	156.47(14)	C1	S1	C11	C12	-121.3(2)
C1	Ni1	P1	C41	39.32(13)	C1	S1	C11	C16	55.3(3)
C1	Ni1	P1	C51	-79.40(14)	Ni1	P1	C4	C5	-19.8(3)
C3	Ni1	P1	C4	103.4(3)	C41	P1	C4	C5	101.1(2)
C3	Ni1	P1	C41	-13.7(3)	C51	P1	C4	C5	-150.6(2)
C3	Ni1	P1	C51	-132.4(2)	Ni1	P1	C41	C42	47.2(2)
P1	Ni1	P2	C6	49.87(11)	C4	P1	C41	C42	-75.4(2)
P1	Ni1	P2	C61	-63.58(10)	C51	P1	C41	C42	174.6(2)
P1	Ni1	P2	C71	172.60(11)	Ni1	P1	C51	C52	-151.4(2)
C1	Ni1	P2	C6	-179.5(4)	C4	P1	C51	C52	-23.4(3)
C1	Ni1	P2	C61	67.0(4)	C41	P1	C51	C52	85.8(3)
C1	Ni1	P2	C71	-56.8(4)	Ni1	P2	C6	C5	-13.2(3)
C3	Ni1	P2	C6	-116.02(14)	C61	P2	C6	C5	103.7(2)
C3	Ni1	P2	C61	130.53(13)	C71	P2	C6	C5	-146.5(2)
C3	Ni1	P2	C71	6.71(14)	Ni1	P2	C61	C62	-52.2(2)
P1	Ni1	C1	S1	67.03(15)	C6	P2	C61	C62	-171.9(2)
P1	Ni1	C1	C2	-178.12(15)	C71	P2	C61	C62	81.0(2)
P2	Ni1	C1	S1	-63.2(4)	Ni1	P2	C71	C72	163.25(19)
P2	Ni1	C1	C2	51.7(4)	C6	P2	C71	C72	-69.4(3)
C3	Ni1	C1	S1	-130.28(18)	C61	P2	C71	C72	38.2(3)
C3	Ni1	C1	C2	-15.43(16)	Ni1	C1	C2	C3	20.0(2)
P1	Ni1	C3	C2	72.4(3)	S1	C1	C2	C3	139.35(19)
P1	Ni1	C3	C21	-41.1(3)	C1	C2	C3	Ni1	-19.7(2)
P1	Ni1	C3	C31	-169.68(15)	C1	C2	C3	C21	75.7(3)
P2	Ni1	C3	C2	-151.31(14)	C1	C2	C3	C31	-145.2(2)
P2	Ni1	C3	C21	95.25(16)	Ni1	C3	C21	C22	-75.0(3)
P2	Ni1	C3	C31	-33.4(2)	Ni1	C3	C21	C26	101.9(3)
C1	Ni1	C3	C2	15.46(16)	C2	C3	C21	C22	-167.8(2)
C1	Ni1	C3	C21	-97.97(18)	C2	C3	C21	C26	9.1(4)
C1	Ni1	C3	C31	133.4(2)	C31	C3	C21	C22	55.7(4)
O1	S1	C1	Ni1	-95.54(16)	C31	C3	C21	C26	-127.4(3)
O1	S1	C1	C2	161.21(18)	Ni1	C3	C31	C32	-60.3(3)
O2	S1	C1	Ni1	35.53(19)	Ni1	C3	C31	C36	122.3(3)
O2	S1	C1	C2	-67.7(2)	C2	C3	C31	C32	46.0(3)
C11	S1	C1	Ni1	149.44(15)	C2	C3	C31	C36	-131.4(3)
C11	S1	C1	C2	46.2(2)	C21	C3	C31	C32	-177.4(3)
O1	S1	C11	C12	121.6(2)	C21	C3	C31	C36	5.2(4)

Table S9. Torsional Angles (continued)

Atom1	Atom2	Atom3	Atom4	Angle	Atom1	Atom2	Atom3	Atom4	Angle
P1	C4	C5	C6	79.6(3)	C24	C25	C26	C21	0.8(4)
C4	C5	C6	P2	-59.9(3)	C3	C31	C32	C33	-177.6(3)
S1	C11	C12	C13	177.8(2)	C36	C31	C32	C33	0.0(4)
C16	C11	C12	C13	1.2(4)	C3	C31	C36	C35	177.6(3)
S1	C11	C16	C15	-177.9(2)	C32	C31	C36	C35	0.1(4)
C12	C11	C16	C15	-1.4(4)	C31	C32	C33	C34	-0.1(5)
C11	C12	C13	C14	-0.4(4)	C32	C33	C34	C35	0.2(5)
C12	C13	C14	C15	-0.3(5)	C33	C34	C35	C36	-0.1(5)
C13	C14	C15	C16	0.2(5)	C34	C35	C36	C31	-0.1(5)
C14	C15	C16	C11	0.6(5)	P1	C41	C42	C43	-149.6(2)
C3	C21	C22	C23	177.1(3)	P1	C41	C42	C44	87.5(3)
C26	C21	C22	C23	0.0(4)	P1	C51	C52	C53	-63.5(3)
C3	C21	C26	C25	-177.6(3)	P1	C51	C52	C54	173.9(2)
C22	C21	C26	C25	-0.5(4)	P2	C61	C62	C63	163.4(2)
C21	C22	C23	C24	0.2(4)	P2	C61	C62	C64	-75.2(3)
C22	C23	C24	C25	0.1(4)	P2	C71	C72	C73	64.6(3)
C23	C24	C25	C26	-0.6(4)	P2	C71	C72	C74	-173.8(2)

Table S10. Anisotropic Displacement Parameters (U_{ij}, Å^2)

Atom	U_{11}	U_{22}	U_{33}	U_{23}	U_{13}	U_{12}
Ni1	0.01481(18)	0.01248(18)	0.01381(18)	-0.00037(14)	0.00137(13)	-0.00101(14)
S1	0.0147(3)	0.0137(3)	0.0163(3)	0.0006(3)	0.0015(3)	-0.0008(3)
P1	0.0187(4)	0.0147(4)	0.0141(4)	0.0008(3)	0.0018(3)	0.0005(3)
P2	0.0148(4)	0.0136(4)	0.0174(4)	0.0009(3)	0.0018(3)	-0.0006(3)
O1	0.0223(11)	0.0152(10)	0.0184(10)	-0.0017(8)	0.0022(8)	-0.0019(8)
O2	0.0153(10)	0.0210(11)	0.0231(11)	0.0028(9)	0.0018(8)	0.0017(8)
C1	0.0157(14)	0.0151(14)	0.0187(14)	-0.0022(11)	0.0013(11)	-0.0014(11)
C2	0.0191(15)	0.0178(15)	0.0150(14)	0.0006(11)	0.0008(11)	-0.0015(12)
C3	0.0166(14)	0.0125(14)	0.0149(14)	-0.0009(11)	0.0015(11)	-0.0011(11)
C4	0.0304(17)	0.0195(16)	0.0161(15)	0.0005(12)	0.0054(12)	-0.0014(13)
C5	0.0301(17)	0.0180(15)	0.0185(15)	0.0032(12)	-0.0025(13)	0.0006(13)
C6	0.0222(16)	0.0173(15)	0.0234(16)	0.0050(12)	0.0012(12)	-0.0047(12)
C11	0.0189(14)	0.0117(14)	0.0160(14)	-0.0018(11)	0.0035(11)	-0.0036(11)
C12	0.0196(15)	0.0153(15)	0.0253(16)	-0.0004(12)	0.0006(12)	-0.0007(12)
C13	0.0275(17)	0.0224(16)	0.0207(16)	0.0037(13)	0.0015(13)	-0.0001(13)
C14	0.0270(16)	0.0234(16)	0.0207(15)	-0.0004(13)	0.0094(13)	-0.0042(13)
C15	0.0196(16)	0.0310(18)	0.0256(16)	0.0005(14)	0.0048(13)	-0.0028(13)
C16	0.0199(15)	0.0248(16)	0.0184(15)	0.0023(12)	-0.0019(12)	-0.0010(12)
C21	0.0170(14)	0.0155(14)	0.0141(14)	-0.0031(11)	0.0021(11)	0.0003(11)
C22	0.0136(13)	0.0150(14)	0.0198(15)	-0.0016(11)	0.0038(11)	-0.0004(11)
C23	0.0209(16)	0.0234(16)	0.0178(15)	0.0035(12)	0.0039(12)	0.0031(12)
C24	0.0149(14)	0.0278(17)	0.0196(15)	0.0019(13)	-0.0011(11)	0.0048(12)
C25	0.0115(14)	0.0270(17)	0.0254(16)	-0.0048(13)	0.0022(12)	-0.0019(12)
C26	0.0162(14)	0.0191(15)	0.0198(15)	-0.0019(12)	0.0060(11)	-0.0015(11)
C31	0.0146(14)	0.0176(14)	0.0143(14)	-0.0009(11)	0.0032(11)	-0.0024(11)
C32	0.0207(15)	0.0171(15)	0.0217(15)	0.0018(12)	0.0026(12)	-0.0018(12)
C33	0.0222(16)	0.0292(18)	0.0194(15)	0.0065(13)	-0.0006(12)	-0.0037(13)
C34	0.0209(16)	0.041(2)	0.0168(15)	-0.0047(14)	-0.0030(12)	-0.0088(14)
C35	0.0220(16)	0.0257(17)	0.0292(17)	-0.0102(14)	0.0043(13)	-0.0054(13)
C36	0.0138(14)	0.0216(16)	0.0220(15)	-0.0014(12)	0.0015(11)	-0.0013(12)
C41	0.0183(15)	0.0240(16)	0.0164(14)	-0.0022(12)	0.0000(11)	0.0007(12)
C42	0.0282(17)	0.0290(18)	0.0179(15)	-0.0037(13)	-0.0047(13)	0.0104(14)
C43	0.0220(17)	0.051(2)	0.0318(19)	0.0015(17)	0.0002(14)	0.0099(16)
C44	0.037(2)	0.0307(19)	0.034(2)	0.0003(15)	-0.0159(16)	0.0064(16)
C51	0.0186(14)	0.0182(15)	0.0192(15)	0.0005(12)	0.0034(11)	0.0032(12)
C52	0.0222(16)	0.0224(16)	0.0240(16)	-0.0002(13)	0.0073(12)	-0.0012(12)
C53	0.0355(19)	0.0343(19)	0.0237(17)	-0.0061(14)	0.0072(14)	0.0034(15)
C54	0.0308(18)	0.0288(18)	0.0307(18)	-0.0054(15)	0.0108(14)	0.0055(14)
C61	0.0163(14)	0.0161(15)	0.0228(15)	0.0002(12)	0.0057(12)	-0.0003(11)
C62	0.0166(15)	0.0187(15)	0.0287(17)	0.0025(13)	0.0039(12)	-0.0002(12)

Table S10. Anisotropic Displacement Parameters (continued)

Atom	U_{11}	U_{22}	U_{33}	U_{23}	U_{13}	U_{12}
C63	0.0308(19)	0.0304(19)	0.039(2)	-0.0073(16)	-0.0047(15)	0.0115(15)
C64	0.0261(17)	0.0240(17)	0.0277(17)	0.0053(14)	0.0013(13)	0.0041(13)
C71	0.0139(14)	0.0179(15)	0.0224(15)	-0.0013(12)	0.0014(11)	0.0009(11)
C72	0.0148(14)	0.0164(15)	0.0267(16)	0.0001(12)	0.0019(12)	-0.0003(11)
C73	0.0205(16)	0.0246(17)	0.0355(19)	0.0066(14)	0.0003(13)	-0.0052(13)
C74	0.0222(16)	0.0236(17)	0.0322(18)	-0.0060(14)	-0.0016(13)	-0.0046(13)

The form of the anisotropic displacement parameter is:
$\exp[-2\pi^2(h^2a^{*2}U_{11} + k^2b^{*2}U_{22} + l^2c^{*2}U_{33} + 2klb^*c^*U_{23} + 2hla^*c^*U_{13} + 2hka^*b^*U_{12})]$

Table S11. Derived Atomic Coordinates and Displacement Parameters for Hydrogen Atoms

Atom	*x*	*y*	*z*	U_{eq}, Å^2
H1	0.084089	0.499638	0.279254	0.020
H2A	0.081834	0.537175	0.397782	0.021
H2B	0.216413	0.508950	0.420203	0.021
H4A	0.423892	0.576083	0.090097	0.026
H4B	0.291323	0.577876	0.049671	0.026
H5A	0.322721	0.694239	0.078063	0.027
H5B	0.247221	0.667862	0.142497	0.027
H6A	0.411035	0.738514	0.189271	0.025
H6B	0.502000	0.685178	0.155070	0.025
H12	0.276265	0.345709	0.458356	0.024
H13	0.166511	0.305955	0.552822	0.028
H14	-0.040661	0.312141	0.541190	0.028
H15	-0.142739	0.358506	0.435562	0.030
H16	-0.035417	0.398731	0.340900	0.026
H22	0.236471	0.732106	0.293089	0.019
H23	0.098182	0.800178	0.222336	0.025
H24	-0.102619	0.765341	0.204979	0.025
H25	-0.162381	0.660854	0.260616	0.026
H26	-0.025098	0.591156	0.330307	0.022
H32	0.388898	0.548562	0.449545	0.024
H33	0.507745	0.592739	0.549007	0.029
H34	0.484849	0.710542	0.587754	0.032
H35	0.339995	0.782990	0.525276	0.031
H36	0.219891	0.739332	0.425724	0.023
H41A	0.143817	0.481488	0.072714	0.024
H41B	0.118709	0.447443	0.149206	0.024
H42	0.056410	0.574225	0.177462	0.031
H43A	-0.100803	0.480061	0.092009	0.052
H43B	-0.143593	0.542342	0.143231	0.052
H43C	-0.072357	0.474770	0.178564	0.052
H44A	0.100201	0.624379	0.064950	0.053
H44B	-0.038560	0.633985	0.076532	0.053
H44C	0.002299	0.573356	0.022755	0.053
H51A	0.447171	0.440388	0.193433	0.022
H51B	0.330830	0.397536	0.162795	0.022
H52	0.489526	0.466379	0.072136	0.027
H53A	0.290133	0.375811	0.029156	0.046
H53B	0.387984	0.397960	-0.024650	0.046
H53C	0.303906	0.457746	0.006201	0.046
H54A	0.581960	0.367810	0.132954	0.044

Table S11. Derived Parameters for Hydrogen Atoms (continued)

Atom	*x*	*y*	*z*	U_{eq}, Å^2
H54B	0.557337	0.349771	0.047989	0.044
H54C	0.472044	0.317342	0.104714	0.044
H61A	0.560733	0.555333	0.204724	0.022
H61B	0.642906	0.610294	0.252941	0.022
H62	0.523836	0.495541	0.319460	0.025
H63A	0.651901	0.445942	0.236422	0.051
H63B	0.699109	0.426507	0.318683	0.051
H63C	0.763016	0.488475	0.276578	0.051
H64A	0.731881	0.573406	0.376075	0.039
H64B	0.669606	0.510915	0.418368	0.039
H64C	0.600965	0.585047	0.401982	0.039
H71A	0.430373	0.743534	0.325389	0.022
H71B	0.481212	0.682589	0.379690	0.022
H72	0.676340	0.700443	0.341392	0.023
H73A	0.636321	0.752758	0.226269	0.041
H73B	0.714937	0.807531	0.277463	0.041
H73C	0.573861	0.820019	0.260863	0.041
H74A	0.567902	0.824726	0.398806	0.039
H74B	0.708267	0.807558	0.408947	0.039
H74C	0.616115	0.757549	0.446746	0.039

S3.2 X-ray Crystallographic Analysis of Ni(dcpe)(N_2CPh_2), Ni-2b

Table S12. Crystallographic Experimental Details

A. Crystal Data	
formula	$C_{47}H_{74}N_2NiO_2P_2$
formula weight	819.73
crystal colour and habit[a]	orange block
crystal dimensions (mm)	0.52 × 0.26 × 0.25
crystal system	orthorhombic
space group	$P2_12_12_1$ (No. 19)
unit cell parameters[b]	
a (Å)	14.3236(5)
b (Å)	16.3223(6)
c (Å)	18.8742(7)
V ($Å^3$)	4412.7(3)
Z	4
ρ_{calcd} (g cm^{-3})	1.234
μ (mm^{-1})	0.551
B. Data Collection and Refinement Conditions	
diffractometer	Bruker D8/APEX II CCD[c]
radiation (λ [Å])	graphite-monochromated Mo Kα (0.71073)
temperature (°C)	–173
scan type	ω scans (0.5°) (30 s exposures)
data collection 2θ limit (deg)	52.42
total data collected	51348 ($-17 \leq h \leq 17$, $-20 \leq k \leq 19$, $-23 \leq l \leq 17$)
independent reflections	8762 (R_{int} = 0.0541)
number of observed reflections (*NO*)	6734 [$F_o^2 \geq 2\sigma(F_o^2)$]
structure solution method	intrinsic phasing (*SHELXT-2018/2*[d])
refinement method	full-matrix least-squares on F^2 (*SHELXL–2019/1*[e,f])
absorption correction method	multi-scan (*SADABS*)
range of transmission factors	0.7453–0.5876
data/restraints/parameters	8762 / 662[g] / 552
Flack absolute structure parameter[h]	–0.016(6)
goodness-of-fit (*S*)[i] [all data]	1.108
final *R* indices[j]	
R_1 [$F_o^2 \geq 2\sigma(F_o^2)$]	0.0617
wR_2 [all data]	0.1886
largest difference peak and hole	0.916 and –0.429 e $Å^{-3}$

[a]Obtained by recrystallization from THF-hexanes. [b]Obtained by least-squares refinement of 9870 reflections with 4.36° < 2θ < 49.98°. [c]Programs for diffractometer operation, data collection, data reduction and absorption correction were those supplied by Bruker. Data collected at the University of Ottawa X-ray facility. [d]Sheldrick, G. M. *Acta Crystallogr.* **2015**, *A71*, 3–8. (*SHELXT-2018/2*). [e]Sheldrick, G. M. *Acta Crystallogr.* **2015**, *C71*, 3–8. (*SHELXL-2019/1*). [f]Attempts to refine peaks of residual electron density as disordered or partial-occupancy solvent tetrahydrofuran oxygen or carbon atoms were unsuccessful for one of the solvent sites. The data were corrected for disordered electron density through use of the SQUEEZE procedure as implemented in *PLATON* (Spek, A. L. *Acta Crystallogr.* **2015**, *C71*, 9–18. *PLATON* - a multipurpose crystallographic tool. Utrecht University, Utrecht, The Netherlands). A total solvent-accessible void volume of 450 $Å^3$ with a total electron count of 150 (consistent with 4 molecules of solvent tetrahydrofuran, or 1 molecule per formula unit of the nickel complex) was found in the unit cell. [g]The disordered cyclohexyl groups had the following restraints applied: sets of atoms were restrained by use of the *SHELXL* **SADI** instruction, P2–C31 & P2–C31A; P2–C41 & P2–C41A; P2···C32, P2···C32A, P2···C36, P2···C36A; P2···C42, P2···C42A, P2···C46, P2···C46A. Additionally, the cyclohexyl rings defined by carbon atoms C31 to C36, C31A to C36A, C41 to C46 and C41A to C46A were restrained to have approximately the same geometry as that of

the well-behaved cyclohexyl group (C51 to C56) by use of the *SHELXL* **SAME** instruction. Furthermore, the anisotropic displacement parameters (ADPs) carbon atoms of the disordered groups were restrained by use of both the **RIGU** and **SIMU** instructions. Finally, the ADPs of the atoms of the solvent tetrahydrofuran molecule were restrained by use of the **RIGU** instruction. Number of restraints generated: **SAME/SADI** (134), **RIGU** (174), **SIMU** (354). [h]Flack, H. D. *Acta Crystallogr.* **1983**, *A39*, 876–881; Flack, H. D.; Bernardinelli, G. *Acta Crystallogr.* **1999**, *A55*, 908–915; Flack, H. D.; Bernardinelli, G. *J. Appl. Cryst.* **2000**, *33*, 1143–1148. The Flack parameter will refine to a value near zero if the structure is in the correct configuration and will refine to a value near one for the inverted configuration. [i]$S = [\Sigma w(F_o^2 - F_c^2)^2/(n-p)]^{1/2}$ (n = number of data; p = number of parameters varied; $w = [\sigma^2(F_o^2) + (0.0984P)^2 + 3.9935P]^{-1}$ where $P = [\mathrm{Max}(F_o^2, 0) + 2F_c^2]/3$). [j]$R_1 = \Sigma||F_o| - |F_c||/\Sigma|F_o|$; $wR_2 = [\Sigma w(F_o^2 - F_c^2)^2/\Sigma w(F_o^4)]^{1/2}$.

Table S13. Atomic Coordinates and Equivalent Isotropic Displacement Parameters

(a) [Ni(dcpe)(N_2CPh_2)] atoms

Atom	*x*	*y*	*z*	U_{eq}, Å^2
Ni1	0.60932(6)	0.46894(5)	0.18784(4)	0.0379(2)*
P1	0.48193(12)	0.54054(11)	0.18850(11)	0.0471(4)*
P2	0.65776(12)	0.53174(12)	0.09420(8)	0.0412(4)*
N1	0.6382(4)	0.3969(4)	0.2609(3)	0.0440(14)*
N2	0.7041(5)	0.3984(4)	0.2174(3)	0.0547(16)*
C1	0.5002(7)	0.6250(6)	0.1260(6)	0.076(3)*
C2	0.5619(7)	0.6002(6)	0.0659(5)	0.068(2)*
C3	0.6222(5)	0.3628(4)	0.3243(3)	0.0447(16)*
C11	0.3731(5)	0.4874(5)	0.1620(4)	0.0502(17)*
C12	0.2994(7)	0.5410(7)	0.1260(5)	0.079(3)*
C13	0.2117(7)	0.4914(7)	0.1153(6)	0.084(3)*
C14	0.2298(8)	0.4165(8)	0.0678(6)	0.097(4)*
C15	0.3054(8)	0.3634(7)	0.1036(7)	0.089(3)*
C16	0.3928(7)	0.4129(6)	0.1177(6)	0.075(3)*
C21	0.4520(6)	0.5894(5)	0.2754(6)	0.067(2)*
C22	0.3715(7)	0.6460(6)	0.2792(6)	0.083(3)*
C23	0.3558(9)	0.6766(8)	0.3546(7)	0.104(4)*
C24	0.4408(9)	0.7150(8)	0.3839(7)	0.103(4)*
C25	0.5207(10)	0.6606(8)	0.3830(7)	0.110(4)*
C26	0.5407(7)	0.6293(7)	0.3063(6)	0.082(3)*
C31[a]	0.7597(8)	0.5998(9)	0.1067(9)	0.047(3)*
C32[a]	0.7409(9)	0.6582(8)	0.1687(8)	0.056(3)*
C33[a]	0.8240(10)	0.7124(9)	0.1845(8)	0.071(4)*
C34[a]	0.9103(8)	0.6648(11)	0.1944(7)	0.068(4)*
C35[a]	0.9305(8)	0.6078(13)	0.1350(8)	0.078(4)*
C36[a]	0.8476(7)	0.5527(11)	0.1206(10)	0.065(4)*
C31A[b]	0.7734(16)	0.585(2)	0.101(2)	0.046(8)*
C32A[b]	0.772(2)	0.647(2)	0.161(3)	0.070(9)*
C33A[b]	0.865(3)	0.680(3)	0.181(3)	0.078(8)*
C34A[b]	0.937(2)	0.619(3)	0.188(2)	0.068(8)*
C35A[b]	0.9418(18)	0.562(3)	0.1264(19)	0.062(8)*
C36A[b]	0.8501(14)	0.523(2)	0.112(2)	0.053(7)*
C41[c]	0.6937(8)	0.4699(6)	0.0157(5)	0.043(2)*
C42[c]	0.7052(17)	0.5164(8)	-0.0524(5)	0.055(4)*
C43[c]	0.7227(16)	0.4654(10)	-0.1162(6)	0.085(6)*
C44[c]	0.6808(11)	0.3861(9)	-0.1201(6)	0.065(4)*
C45[c]	0.6644(13)	0.3414(8)	-0.0530(7)	0.063(4)*
C46[c]	0.6315(10)	0.3954(7)	0.0057(6)	0.059(3)*
C41A[d]	0.652(3)	0.4715(14)	0.0113(11)	0.043(7)*

Table S13. Atomic Coordinates and Displacement Parameters (continued)

Atom	x	y	z	U_{eq}, Å^2
C42A[d]	0.691(6)	0.514(2)	-0.0524(14)	0.051(9)*
C43A[d]	0.749(3)	0.465(2)	-0.100(2)	0.055(8)*
C44A[d]	0.721(4)	0.380(2)	-0.1068(17)	0.060(9)*
C45A[d]	0.691(4)	0.3361(19)	-0.0433(16)	0.046(8)*
C46A[d]	0.686(2)	0.3863(11)	0.0208(12)	0.030(6)*
C51	0.5291(5)	0.3816(4)	0.3549(4)	0.0461(17)*
C52	0.4475(5)	0.3598(4)	0.3191(4)	0.0545(18)*
C53	0.3601(6)	0.3791(5)	0.3476(5)	0.066(2)*
C54	0.3515(7)	0.4199(6)	0.4121(5)	0.073(3)*
C55	0.4338(7)	0.4423(6)	0.4486(5)	0.076(3)*
C56	0.5204(6)	0.4216(5)	0.4206(4)	0.056(2)*
C61	0.6905(5)	0.3143(4)	0.3623(4)	0.0434(16)*
C62	0.6732(5)	0.2782(4)	0.4280(4)	0.0455(16)*
C63	0.7414(7)	0.2346(5)	0.4629(4)	0.058(2)*
C64	0.8297(7)	0.2260(5)	0.4354(4)	0.063(2)*
C65	0.8478(7)	0.2601(5)	0.3704(5)	0.064(2)*
C66	0.7802(5)	0.3034(5)	0.3338(4)	0.0506(18)*

(b) solvent tetrahydrofuran atoms

Atom	x	y	z	U_{eq}, Å^2
O1S	0.4035(8)	0.5250(8)	-0.0686(5)	0.137(3)*
C1S	0.3900(11)	0.4550(11)	-0.1110(7)	0.125(4)*
C2S	0.3862(15)	0.4844(14)	-0.1841(8)	0.161(6)*
C3S	0.4409(16)	0.5606(14)	-0.1827(9)	0.169(7)*
C4S	0.4324(12)	0.5869(11)	-0.1132(7)	0.137(5)*

Anisotropically-refined atoms are marked with an asterisk (*). The form of the anisotropic displacement parameter is: $\exp[-2\pi^2(h^2a^{*2}U_{11} + k^2b^{*2}U_{22} + l^2c^{*2}U_{33} + 2klb^*c^*U_{23} + 2hla^*c^*U_{13} + 2hka^*b^*U_{12})]$. Refined occupancies of 0.75(3)[a], 0.25(3)[b], 0.77(2)[c], 0.23(2)[d].

Table S14. Selected Interatomic Distances (Å)

(a) within the [Ni(dcpe)(N_2CPh_2)] molecule

Atom1	Atom2	Distance
Ni1	P1	2.1669(19)
Ni1	P2	2.1576(18)
Ni1	N1	1.859(6)
Ni1	N2	1.866(7)
P1	C1	1.833(8)
P1	C11	1.854(7)
P1	C21	1.874(10)
P2	C2	1.849(9)
P2	C31	1.850(10)†
P2	C31A	1.87(2)†
P2	C41	1.865(9)†
P2	C41A	1.85(2)†
N1	N2	1.251(8)
N1	C3	1.340(8)
C1	C2	1.494(14)
C3	C51	1.485(10)
C3	C61	1.448(10)
C11	C12	1.530(11)
C11	C16	1.502(11)
C12	C13	1.509(14)
C13	C14	1.539(17)
C14	C15	1.542(16)
C15	C16	1.514(13)
C21	C22	1.479(12)
C21	C26	1.542(13)
C22	C23	1.526(16)
C23	C24	1.476(17)
C24	C25	1.449(17)
C25	C26	1.562(15)
C31	C32	1.533(13)†
C31	C36	1.496(13)†
C32	C33	1.513(13)†
C33	C34	1.473(15)†

Atom1	Atom2	Distance
C34	C35	1.485(15)†
C35	C36	1.514(14)†
C31A	C32A	1.51(2)†
C31A	C36A	1.50(2)†
C32A	C33A	1.49(2)†
C33A	C34A	1.45(2)†
C34A	C35A	1.49(2)†
C35A	C36A	1.48(2)†
C41	C42	1.503(11)†
C41	C46	1.518(13)†
C42	C43	1.484(13)†
C43	C44	1.428(15)†
C44	C45	1.480(13)†
C45	C46	1.491(13)†
C41A	C42A	1.50(2)†
C41A	C46A	1.49(2)†
C42A	C43A	1.48(2)†
C43A	C44A	1.44(2)†
C44A	C45A	1.46(2)†
C45A	C46A	1.46(2)†
C51	C52	1.396(10)
C51	C56	1.407(10)
C52	C53	1.399(11)
C53	C54	1.392(12)
C54	C55	1.415(13)
C55	C56	1.390(11)
C61	C62	1.394(10)
C61	C66	1.404(11)
C62	C63	1.376(11)
C63	C64	1.375(13)
C64	C65	1.371(12)
C65	C66	1.383(11)

(b) within the solvent tetrahydrofuran molecules

Atom1	Atom2	Distance
O1S	C1S	1.409(17)
O1S	C4S	1.379(17)
C1S	C2S	1.462(18)

Atom1	Atom2	Distance
C2S	C3S	1.47(3)
C3S	C4S	1.39(2

†Restrained during refinement.

Table S15. Selected Interatomic Angles (deg)

(a) within the [Ni(dcpe)(N_2CPh_2)] molecule

Atom1	Atom2	Distance		Atom1	Atom2	Distance	
P1	Ni1	P2	91.10(8)	C11	C16	C15	112.0(8)
P1	Ni1	N1	121.63(18)	P1	C21	C22	119.1(8)
P1	Ni1	N2	160.7(2)	P1	C21	C26	108.8(6)
P2	Ni1	N1	146.77(19)	C22	C21	C26	111.2(7)
P2	Ni1	N2	107.7(2)	C21	C22	C23	111.3(10)
N1	Ni1	N2	39.3(3)	C22	C23	C24	111.5(10)
Ni1	P1	C1	106.4(3)	C23	C24	C25	112.8(10)
Ni1	P1	C11	117.0(2)	C24	C25	C26	110.8(11)
Ni1	P1	C21	115.3(3)	C21	C26	C25	109.7(9)
C1	P1	C11	107.3(4)	P2	C31	C32	109.4(8)†
C1	P1	C21	106.0(5)	P2	C31	C36	112.2(9)†
C11	P1	C21	104.1(4)	C32	C31	C36	109.5(10)†
Ni1	P2	C2	106.6(3)	C31	C32	C33	112.1(9)†
Ni1	P2	C31	115.7(5)	C32	C33	C34	112.1(10)†
Ni1	P2	C31A	116.4(14)	C33	C34	C35	113.5(11)†
Ni1	P2	C41	118.8(3)	C34	C35	C36	110.8(10)†
Ni1	P2	C41A	115.2(9)	C31	C36	C35	112.7(11)†
C2	P2	C31	105.1(6)	P2	C31A	C32A	110.5(17)†
C2	P2	C31A	113.4(12)	P2	C31A	C36A	110.3(18)†
C2	P2	C41	107.6(5)	C32A	C31A	C36A	111(2)†
C2	P2	C41A	92.4(10)	C31A	C32A	C33A	115(2)†
C31	P2	C41	102.0(6)	C32A	C33A	C34A	114(2)†
C31A	P2	C41A	110.3(17)	C33A	C34A	C35A	114(2)†
Ni1	N1	N2	70.7(4)	C34A	C35A	C36A	111(2)†
Ni1	N1	C3	152.5(5)	C31A	C36A	C35A	113(2)†
N2	N1	C3	136.3(7)	P2	C41	C42	115.9(8)†
Ni1	N2	N1	70.1(4)	P2	C41	C46	111.8(7)†
P1	C1	C2	111.7(6)	C42	C41	C46	111.2(11)†
P2	C2	C1	112.5(6)	C41	C42	C43	115.4(10)†
N1	C3	C51	114.5(6)	C42	C43	C44	118.6(11)†
N1	C3	C61	123.6(7)	C43	C44	C45	118.0(10)†
C51	C3	C61	121.8(6)	C44	C45	C46	113.2(10)†
P1	C11	C12	115.6(6)	C41	C46	C45	112.4(10)†
P1	C11	C16	111.8(5)	P2	C41A	C42A	114.5(19)†
C12	C11	C16	110.2(8)	P2	C41A	C46A	112.3(16)†
C11	C12	C13	109.1(8)	C42A	C41A	C46A	114(2)†
C12	C13	C14	111.3(9)	C41A	C42A	C43A	117(2)†
C13	C14	C15	108.0(8)	C42A	C43A	C44A	115(3)†
C14	C15	C16	110.9(9)	C43A	C44A	C45A	119(2)†

Table S16. Selected Interatomic Angles (continued)

Atom1	Atom2	Atom3	Angle	Atom1	Atom2	Atom3	Angle
C44A	C45A	C46A	115(2)†	C51	C56	C55	121.9(8)
C41A	C46A	C45A	116(2)†	C3	C61	C62	123.4(7)
C3	C51	C52	120.8(6)	C3	C61	C66	119.9(7)
C3	C51	C56	121.2(7)	C62	C61	C66	116.7(7)
C52	C51	C56	118.1(7)	C61	C62	C63	121.2(8)
C51	C52	C53	120.4(7)	C62	C63	C64	121.6(8)
C52	C53	C54	121.5(8)	C63	C64	C65	118.0(8)
C53	C54	C55	118.4(8)	C64	C65	C66	121.5(9)
C54	C55	C56	119.7(8)	C61	C66	C65	120.9(7)

(b) within the solvent tetrahydrofuran molecules

Atom1	Atom2	Atom3	Angle	Atom1	Atom2	Atom3	Angle
C1S	O1S	C4S	106.8(12)	C2S	C3S	C4S	103.4(17)
O1S	C1S	C2S	105.9(15)	O1S	C4S	C3S	112.2(18)
C1S	C2S	C3S	103.9(16)				

†Restrained during refinement.

Table S17. Torsional Angles (deg)

Atom1	Atom2	Atom3	Atom4	Angle	Atom1	Atom2	Atom3	Atom4	Angle
P2	Ni1	P1	C1	-12.7(4)	Ni1	P1	C21	C22	174.1(6)
P2	Ni1	P1	C11	107.2(3)	Ni1	P1	C21	C26	45.3(8)
P2	Ni1	P1	C21	-129.9(3)	C1	P1	C21	C22	56.7(8)
N1	Ni1	P1	C1	161.2(4)	C1	P1	C21	C26	-72.1(8)
N1	Ni1	P1	C11	-78.9(4)	C11	P1	C21	C22	-56.4(8)
N1	Ni1	P1	C21	44.0(4)	C11	P1	C21	C26	174.9(7)
N2	Ni1	P1	C1	154.9(8)	Ni1	P2	C2	C1	26.4(7)
N2	Ni1	P1	C11	-85.2(7)	C31	P2	C2	C1	-97.0(9)
N2	Ni1	P1	C21	37.7(7)	C31A	P2	C2	C1	-103.1(15)
P1	Ni1	P2	C2	-4.8(3)	C41	P2	C2	C1	154.8(7)
P1	Ni1	P2	C31	111.6(5)	C41A	P2	C2	C1	143.6(13)
P1	Ni1	P2	C31A	122.9(11)	Ni1	P2	C31	C32	-51.4(10)
P1	Ni1	P2	C41	-126.4(4)	Ni1	P2	C31	C36	70.3(11)
P1	Ni1	P2	C41A	-105.7(12)	C2	P2	C31	C32	65.9(10)
N1	Ni1	P2	C2	-175.4(5)	C2	P2	C31	C36	-172.4(10)
N1	Ni1	P2	C31	-59.0(6)	C41	P2	C31	C32	178.1(9)
N1	Ni1	P2	C31A	-47.7(12)	C41	P2	C31	C36	-60.2(11)
N1	Ni1	P2	C41	63.0(5)	Ni1	P2	C31A	C32A	-58(3)
N1	Ni1	P2	C41A	83.7(13)	Ni1	P2	C31A	C36A	65(3)
N2	Ni1	P2	C2	179.5(4)	C2	P2	C31A	C32A	67(3)
N2	Ni1	P2	C31	-64.1(5)	C2	P2	C31A	C36A	-170(2)
N2	Ni1	P2	C31A	-52.9(11)	C41A	P2	C31A	C32A	169(3)
N2	Ni1	P2	C41	57.9(5)	C41A	P2	C31A	C36A	-68(3)
N2	Ni1	P2	C41A	78.6(12)	Ni1	P2	C41	C42	166.4(11)
P1	Ni1	N1	N2	-176.7(4)	Ni1	P2	C41	C46	37.6(12)
P1	Ni1	N1	C3	-5.7(12)	C2	P2	C41	C42	45.3(13)
P2	Ni1	N1	N2	-7.7(6)	C2	P2	C41	C46	-83.5(11)
P2	Ni1	N1	C3	163.2(9)	C31	P2	C41	C42	-65.0(14)
N2	Ni1	N1	C3	171.0(14)	C31	P2	C41	C46	166.2(11)
P1	Ni1	N2	N1	8.6(9)	Ni1	P2	C41A	C42A	-177(3)
P2	Ni1	N2	N1	175.6(4)	Ni1	P2	C41A	C46A	-44(3)
Ni1	P1	C1	C2	32.4(8)	C2	P2	C41A	C42A	74(3)
C11	P1	C1	C2	-93.6(7)	C2	P2	C41A	C46A	-154(3)
C21	P1	C1	C2	155.6(7)	C31A	P2	C41A	C42A	-42(4)
Ni1	P1	C11	C12	-149.3(6)	C31A	P2	C41A	C46A	90(3)
Ni1	P1	C11	C16	-22.0(7)	C3	N1	N2	Ni1	-174.0(9)
C1	P1	C11	C12	-29.9(8)	Ni1	N1	C3	C51	11.7(14)
C1	P1	C11	C16	97.3(7)	Ni1	N1	C3	C61	-165.8(8)
C21	P1	C11	C12	82.2(7)	N2	N1	C3	C51	179.3(8)
C21	P1	C11	C16	-150.5(7)	N2	N1	C3	C61	1.8(13)

Table S17. Torsional Angles (continued)

Atom1	Atom2	Atom3	Atom4	Angle	Atom1	Atom2	Atom3	Atom4	Angle
P1	C1	C2	P2	-38.0(9)	C33A	C34A	C35A	C36A	-54(4)
N1	C3	C51	C52	59.7(9)	C34A	C35A	C36A	C31A	55(4)
N1	C3	C51	C56	-120.5(7)	P2	C41	C42	C43	-173.0(14)
C61	C3	C51	C52	-122.8(7)	C46	C41	C42	C43	-44(2)
C61	C3	C51	C56	57.0(10)	P2	C41	C46	C45	-175.0(9)
N1	C3	C61	C62	-179.4(7)	C42	C41	C46	C45	53.8(17)
N1	C3	C61	C66	2.3(10)	C41	C42	C43	C44	33(3)
C51	C3	C61	C62	3.3(10)	C42	C43	C44	C45	-30(2)
C51	C3	C61	C66	-175.1(7)	C43	C44	C45	C46	40(2)
P1	C11	C12	C13	-173.6(7)	C44	C45	C46	C41	-51.5(18)
C16	C11	C12	C13	58.3(10)	P2	C41A	C42A	C43A	137(5)
P1	C11	C16	C15	172.9(8)	C46A	C41A	C42A	C43A	6(6)
C12	C11	C16	C15	-57.1(11)	P2	C41A	C46A	C45A	-175(3)
C11	C12	C13	C14	-60.7(11)	C42A	C41A	C46A	C45A	-43(5)
C12	C13	C14	C15	59.2(12)	C41A	C42A	C43A	C44A	35(7)
C13	C14	C15	C16	-55.9(13)	C42A	C43A	C44A	C45A	-41(6)
C14	C15	C16	C11	56.6(13)	C43A	C44A	C45A	C46A	5(7)
P1	C21	C22	C23	177.6(7)	C44A	C45A	C46A	C41A	38(6)
C26	C21	C22	C23	-54.7(12)	C3	C51	C52	C53	-178.8(7)
P1	C21	C26	C25	-172.8(8)	C56	C51	C52	C53	1.4(10)
C22	C21	C26	C25	54.2(13)	C3	C51	C56	C55	177.5(7)
C21	C22	C23	C24	55.3(14)	C52	C51	C56	C55	-2.7(11)
C22	C23	C24	C25	-56.8(16)	C51	C52	C53	C54	-0.3(12)
C23	C24	C25	C26	56.8(15)	C52	C53	C54	C55	0.4(13)
C24	C25	C26	C21	-54.7(13)	C53	C54	C55	C56	-1.6(13)
P2	C31	C32	C33	176.7(11)	C54	C55	C56	C51	2.8(13)
C36	C31	C32	C33	53.4(15)	C3	C61	C62	C63	-178.1(7)
P2	C31	C36	C35	-176.7(12)	C66	C61	C62	C63	0.3(10)
C32	C31	C36	C35	-55.1(15)	C3	C61	C66	C65	177.3(7)
C31	C32	C33	C34	-52.7(15)	C62	C61	C66	C65	-1.2(11)
C32	C33	C34	C35	52.9(15)	C61	C62	C63	C64	1.3(11)
C33	C34	C35	C36	-53.5(17)	C62	C63	C64	C65	-2.1(12)
C34	C35	C36	C31	55.3(18)	C63	C64	C65	C66	1.2(13)
P2	C31A	C32A	C33A	168(4)	C64	C65	C66	C61	0.4(13)
C36A	C31A	C32A	C33A	45(5)	C4S	O1S	C1S	C2S	-15.9(17)
P2	C31A	C36A	C35A	-174(3)	C1S	O1S	C4S	C3S	-2(2)
C32A	C31A	C36A	C35A	-51(4)	O1S	C1S	C2S	C3S	26.4(19)
C31A	C32A	C33A	C34A	-45(5)	C1S	C2S	C3S	C4S	-27(2)
C32A	C33A	C34A	C35A	48(5)	C2S	C3S	C4S	O1S	18(2)

Table S18. Anisotropic Displacement Parameters (U_{ij}, Å^2)

Atom	U_{11}	U_{22}	U_{33}	U_{23}	U_{13}	U_{12}
Ni1	0.0363(4)	0.0415(4)	0.0358(4)	0.0053(4)	0.0011(3)	0.0000(4)
P1	0.0357(8)	0.0365(9)	0.0692(11)	0.0043(10)	0.0054(8)	0.0003(7)
P2	0.0415(9)	0.0464(9)	0.0357(8)	0.0083(8)	-0.0043(7)	-0.0031(9)
N1	0.048(4)	0.047(3)	0.037(3)	0.003(2)	0.010(3)	-0.002(3)
N2	0.063(4)	0.058(4)	0.043(3)	0.001(3)	-0.009(3)	0.006(3)
C1	0.060(5)	0.059(5)	0.109(8)	0.041(5)	0.001(5)	0.000(4)
C2	0.076(6)	0.064(5)	0.062(5)	0.029(4)	-0.019(5)	-0.006(5)
C3	0.055(4)	0.046(4)	0.033(3)	0.000(3)	0.001(3)	0.000(3)
C11	0.038(4)	0.055(4)	0.057(4)	0.008(3)	-0.004(3)	0.003(3)
C12	0.071(6)	0.098(8)	0.067(5)	0.018(6)	-0.018(5)	0.014(6)
C13	0.063(6)	0.111(9)	0.079(6)	0.014(6)	-0.030(5)	0.000(6)
C14	0.076(7)	0.131(10)	0.085(7)	-0.002(7)	-0.020(6)	-0.022(7)
C15	0.078(7)	0.074(7)	0.116(9)	-0.017(6)	-0.019(6)	-0.017(6)
C16	0.052(5)	0.070(6)	0.103(7)	-0.019(5)	-0.001(5)	-0.008(5)
C21	0.046(5)	0.048(4)	0.106(7)	-0.014(4)	0.009(5)	-0.004(4)
C22	0.066(6)	0.066(6)	0.117(8)	-0.001(6)	0.006(6)	0.003(5)
C23	0.097(9)	0.093(8)	0.122(10)	-0.027(7)	0.048(8)	0.006(7)
C24	0.106(9)	0.086(8)	0.117(10)	-0.035(7)	0.036(8)	-0.007(7)
C25	0.125(11)	0.096(9)	0.108(10)	-0.049(8)	0.000(8)	-0.005(8)
C26	0.064(6)	0.081(6)	0.100(7)	-0.029(6)	0.005(6)	0.000(5)
C31	0.043(5)	0.056(6)	0.042(6)	0.009(5)	-0.003(5)	-0.009(5)
C32	0.042(6)	0.068(7)	0.058(7)	-0.007(5)	0.004(5)	-0.006(5)
C33	0.057(7)	0.080(8)	0.075(7)	-0.007(6)	-0.002(6)	-0.019(6)
C34	0.037(6)	0.103(10)	0.065(7)	-0.011(7)	0.006(5)	-0.020(6)
C35	0.051(6)	0.110(11)	0.071(8)	-0.014(8)	0.015(5)	-0.019(6)
C36	0.038(5)	0.090(9)	0.068(8)	-0.006(7)	0.005(5)	-0.003(5)
C31A	0.050(13)	0.061(15)	0.028(13)	0.005(11)	0.005(10)	-0.018(11)
C32A	0.068(14)	0.082(15)	0.059(15)	-0.015(13)	0.000(12)	-0.010(12)
C33A	0.066(14)	0.096(15)	0.071(16)	-0.017(13)	-0.004(13)	-0.014(12)
C34A	0.059(13)	0.091(16)	0.055(14)	-0.007(14)	0.000(11)	-0.020(11)
C35A	0.054(12)	0.078(16)	0.052(14)	0.002(13)	0.004(9)	-0.020(10)
C36A	0.049(11)	0.068(14)	0.041(14)	0.003(11)	0.004(9)	-0.019(10)
C41	0.042(6)	0.054(5)	0.034(4)	0.007(4)	-0.001(4)	-0.008(5)
C42	0.068(9)	0.064(6)	0.032(5)	0.008(4)	0.003(4)	-0.017(5)
C43	0.148(14)	0.080(7)	0.028(5)	0.001(5)	0.015(7)	-0.018(8)
C44	0.072(9)	0.085(7)	0.039(5)	-0.006(5)	0.000(5)	-0.010(6)
C45	0.073(11)	0.063(6)	0.054(6)	-0.007(5)	0.003(6)	-0.008(6)
C46	0.072(8)	0.056(6)	0.049(5)	0.000(4)	0.011(5)	-0.018(5)
C41A	0.055(16)	0.042(11)	0.032(11)	0.005(8)	0.006(11)	0.003(10)
C42A	0.063(18)	0.054(12)	0.036(12)	0.010(10)	0.009(12)	-0.001(11)

Table S18. Anisotropic Displacement Parameters (continued)

Atom	U_{11}	U_{22}	U_{33}	U_{23}	U_{13}	U_{12}
C43A	0.052(14)	0.069(12)	0.044(14)	0.003(10)	0.005(12)	-0.003(11)
C44A	0.07(2)	0.066(12)	0.040(11)	0.001(9)	0.015(12)	0.003(12)
C45A	0.05(2)	0.051(11)	0.032(11)	-0.003(8)	0.000(11)	0.003(10)
C46A	0.026(13)	0.038(10)	0.027(9)	0.004(7)	0.002(8)	-0.008(9)
C51	0.051(4)	0.044(4)	0.044(4)	0.014(3)	0.009(3)	0.002(3)
C52	0.062(5)	0.039(4)	0.062(5)	0.007(4)	-0.001(4)	-0.001(3)
C53	0.049(5)	0.059(5)	0.090(7)	0.020(5)	0.001(4)	-0.001(4)
C54	0.062(5)	0.081(6)	0.078(6)	0.029(5)	0.021(5)	0.010(5)
C55	0.098(8)	0.081(6)	0.049(5)	0.012(4)	0.023(5)	0.010(6)
C56	0.070(5)	0.057(5)	0.040(4)	0.007(3)	0.006(4)	0.000(4)
C61	0.055(4)	0.038(4)	0.037(3)	-0.010(3)	-0.008(3)	-0.001(3)
C62	0.053(4)	0.042(4)	0.042(4)	-0.002(3)	-0.008(3)	-0.003(3)
C63	0.088(6)	0.045(4)	0.041(4)	0.002(3)	-0.019(4)	-0.010(4)
C64	0.077(6)	0.053(5)	0.060(5)	-0.002(4)	-0.023(4)	0.015(4)
C65	0.067(5)	0.058(5)	0.068(5)	0.000(4)	-0.005(4)	0.011(4)
C66	0.059(5)	0.047(4)	0.047(4)	0.002(3)	0.001(3)	0.009(4)
O1S	0.126(7)	0.190(9)	0.095(5)	0.009(6)	0.000(5)	-0.040(8)
C1S	0.095(8)	0.192(11)	0.087(7)	-0.005(7)	0.002(7)	-0.006(9)
C2S	0.181(16)	0.216(15)	0.086(7)	0.007(8)	-0.011(8)	0.003(12)
C3S	0.177(17)	0.214(15)	0.116(9)	0.030(9)	0.009(10)	0.021(12)
C4S	0.133(12)	0.183(11)	0.094(7)	0.050(7)	-0.012(8)	0.006(9)

The form of the anisotropic displacement parameter is:
$\exp[-2\pi^2(h^2a^{*2}U_{11} + k^2b^{*2}U_{22} + l^2c^{*2}U_{33} + 2klb^*c^*U_{23} + 2hla^*c^*U_{13} + 2hka^*b^*U_{12})]$

Table S19. Derived Atomic Coordinates and Displacement Parameters for Hydrogen Atoms

Atom	*x*	*y*	*z*	U_{eq}, Å^2
H1A	0.439086	0.643069	0.107068	0.091
H1B	0.528725	0.671874	0.151223	0.091
H2A	0.588883	0.649967	0.043915	0.081
H2B	0.523909	0.571978	0.029503	0.081
H11	0.343972	0.466922	0.206748	0.060
H12A	0.285798	0.589374	0.155885	0.094
H12B	0.323065	0.560494	0.079691	0.094
H13A	0.163140	0.526304	0.093368	0.101
H13B	0.188017	0.472715	0.161915	0.101
H14A	0.251408	0.434520	0.020431	0.116
H14B	0.171674	0.384491	0.061762	0.116
H15A	0.280921	0.341450	0.148817	0.107
H15B	0.320799	0.316380	0.072520	0.107
H16A	0.438962	0.377981	0.142429	0.090
H16B	0.420381	0.430142	0.072000	0.090
H21	0.436498	0.543150	0.308087	0.080
H22A	0.314657	0.617378	0.262450	0.100
H22B	0.382696	0.693323	0.247535	0.100
H23A	0.304270	0.717016	0.354797	0.125
H23B	0.337151	0.630044	0.385131	0.125
H24A	0.455631	0.764669	0.356008	0.124
H24B	0.428436	0.732254	0.433295	0.124
H25A	0.508613	0.613253	0.414516	0.132
H25B	0.576174	0.689986	0.401254	0.132
H26A	0.560229	0.675729	0.275992	0.098
H26B	0.592069	0.588753	0.307298	0.098
H31[a]	0.768479	0.633007	0.062723	0.056
H32A[a]	0.686362	0.692989	0.157255	0.067
H32B[a]	0.725420	0.625798	0.211461	0.067
H33A[a]	0.832848	0.751485	0.144935	0.085
H33B[a]	0.811332	0.744556	0.227943	0.085
H34A[a]	0.905236	0.632847	0.238843	0.082
H34B[a]	0.963426	0.703140	0.199707	0.082
H35A[a]	0.985487	0.573775	0.147020	0.093
H35B[a]	0.945422	0.639644	0.091895	0.093
H36A[a]	0.861490	0.517830	0.079001	0.078
H36B[a]	0.837736	0.516266	0.161819	0.078
H31A[b]	0.785491	0.614434	0.055874	0.055
H32C[b]	0.731267	0.692812	0.146247	0.083
H32D[b]	0.743084	0.620699	0.202683	0.083

Table S19. Derived Parameters for Hydrogen Atoms (continued)

Atom	*x*	*y*	*z*	U_{eq}, Å^2
H33C[b]	0.884151	0.721090	0.145718	0.093
H33D[b]	0.858097	0.708760	0.227361	0.093
H34C[b]	0.925925	0.586646	0.231451	0.082
H34D[b]	0.997911	0.646731	0.192856	0.082
H35C[b]	0.962433	0.592024	0.083812	0.074
H35D[b]	0.988427	0.518381	0.136322	0.074
H36C[b]	0.833482	0.487480	0.152738	0.063
H36D[b]	0.855155	0.488394	0.069607	0.063
H41[c]	0.756966	0.447771	0.027389	0.052
H42A[c]	0.648067	0.549167	-0.060620	0.066
H42B[c]	0.757829	0.555160	-0.046879	0.066
H43A[c]	0.701692	0.496977	-0.158035	0.103
H43B[c]	0.791039	0.458154	-0.120710	0.103
H44A[c]	0.720724	0.351335	-0.150538	0.078
H44B[c]	0.619915	0.391977	-0.144438	0.078
H45A[c]	0.723205	0.314408	-0.038311	0.076
H45B[c]	0.617353	0.298076	-0.061278	0.076
H46A[c]	0.629930	0.363538	0.050273	0.071
H46B[c]	0.567109	0.413890	-0.004692	0.071
H41A[d]	0.583587	0.465632	0.001275	0.051
H42C[d]	0.637833	0.536358	-0.080217	0.061
H42D[d]	0.728229	0.561402	-0.035893	0.061
H43C[d]	0.748412	0.489909	-0.148002	0.066
H43D[d]	0.814595	0.466232	-0.083027	0.066
H44C[d]	0.774056	0.349496	-0.127722	0.072
H44D[d]	0.669252	0.377878	-0.141375	0.072
H45C[d]	0.735322	0.290416	-0.034767	0.055
H45D[d]	0.629046	0.312003	-0.052342	0.055
H46C[d]	0.749198	0.388717	0.042180	0.037
H46D[d]	0.644737	0.358248	0.055113	0.037
H52	0.451346	0.331646	0.275173	0.065
H53	0.305301	0.364040	0.322420	0.079
H54	0.291712	0.432391	0.431081	0.088
H55	0.430026	0.471481	0.492100	0.091
H56	0.575212	0.434697	0.446520	0.067
H62	0.613341	0.283919	0.449007	0.055
H63	0.727004	0.209797	0.507098	0.070
H64	0.876832	0.197278	0.460641	0.076
H65	0.908044	0.253922	0.350124	0.077
H66	0.794634	0.325991	0.288763	0.061

Table S19. Derived Parameters for Hydrogen Atoms (continued)

Atom	*x*	*y*	*z*	U_{eq}, Å^2
H1SA	0.331033	0.427035	-0.098100	0.150
H1SB	0.442306	0.415978	-0.104785	0.150
H2SA	0.320944	0.495120	-0.198795	0.194
H2SB	0.414178	0.443998	-0.216983	0.194
H3SA	0.507003	0.550074	-0.195016	0.203
H3SB	0.414977	0.601476	-0.216099	0.203
H4SA	0.493359	0.608101	-0.096743	0.164
H4SB	0.386805	0.632480	-0.111188	0.164

Refined occupancies of 0.75(3)[a], 0.25(3)[b], 0.77(2)[c], 0.23(2)[d].

S4. Computational Details

Geometry Optimization. Molecular structures were constructed with the combined use of Chimera[9] (version 1.15) and Spartan[10] (version 24-1.2.0), which was also used to perform conformational searches using the MMFF94 force field method[11] coupled with constraints on the geometry of the metal center. Molecular geometries were then optimized using the r^{2}scan-3c[12] composite method implemented in Orca[13] (version 6.0.1) using the def2-mTZVPP basis set.[12] Geometries were converged until meeting the default converge criteria (energy change < 5.0000e-06 Eh, max. gradient < 3.0000e-04 Eh/bohr, RMS gradient < 1.0000e-04 Eh/bohr, max. displacement < 4.0000e-03 bohr, RMS displacement < 2.0000e-03 bohr, strict convergence = False). The electronic states were converged to "TightSCF" criteria (energy change < 1.0000e-08 a.u., max density change < 1.0000e-07 a.u., RMS-density change < 5.0000e-09 a.u., DIIS error < 5.0000e-07 a.u., orbital gradient < 1.0000e-05 a.u., orbital rotation < 1.0000e-05 a.u.) while deploying the "SlowConv" strategy with restricted or unrestricted for singlet or triplet spin states respectively. The nature of all critical points was confirmed by the analysis of the analytical Hessian's eigenvalues computed at the optimized geometry to confirm the positive curvature for minima and a single imaginary mode for transition states. The thermochemistry corrections, which were computed with the default ideal gas approximation for a temperature of 298 K and 1 atm pressure, were used to compute the corrections of the electronic energy later used to calculate Gibbs free energy as described below.

Single Point Energy Calculation. Single point electronic energies were computed with Orca[13] (version 6.0.1) using its implementation of the hybrid DFT functional[14] M06 coupled with zero-damping DFT-D3 empirical dispersion correction[15] and the CPCM solvation model for benzene with default configuration (i.e., Gaussian vdW surface and default element-dependent radii). All atoms were described by the def2-QZVPP[16] basis set, coupled with the corresponding def2-QZVPP/C and def2/J auxiliary basis sets for the resolution of the identity approximation termed "RIJCOSX".[17] The integration grid was set to "DEFGRID2". The electronic states were converged to "TightSCF" criteria (energy change < 1.0000e-08 a.u., max density change < 1.0000e-07 a.u., RMS-density change < 5.0000e-09 a.u., DIIS error < 5.0000e-07 a.u., orbital gradient < 1.0000e-05 a.u., orbital rotation < 1.0000e-05 a.u.) while deploying the "SlowConv" strategy with restricted or unrestricted for singlet or triplet spin states respectively.

Gibbs Free Energy Calculation. Gibbs free energies at 298.15 K were calculated via equation 1

$$G^{298\text{ K [1M]}} = E_{M06\text{-}D3ZERO} + G^{298.15\text{ K}}_{corr,\, r2scan\text{-}3c} + G^{298.15\text{ K}}_{1\text{atm}\rightarrow 1\text{M}} \qquad \text{Eq. 1}$$

considering the electronic energy from the single point energy model based on the M06 functional ($E_{M06\text{-}D3ZERO}$), the thermochemical correction to the Gibbs free energy obtained from the composite method r2scan-3c ($G^{298.15\text{ K}}_{corr,\, r2scan\text{-}3c}$), which was calculated by subtracting the electronic energy ($E_{r2scan\text{-}3c}$) to the Gibbs free energy ($G^{298.15\text{ K}}_{r2scan\text{-}3c}$) computed by the geometry optimization model, and the standard state correction ($G^{298.15\text{ K}}_{1\text{atm}\rightarrow 1\text{M}}$) corresponding to 1 M solution (but exhibiting infinite-dilution, ideal-gas-like behavior), which is equal to 1.89 kcal/mol (= RT·ln(24.46)) at room temperature. All values are reported in Table S20.

Natural Bond Order Analysis. The NBO program[18] (version 7.0.10) was used to perform natural bond orbital (NBO) and natural resonance theory (NRT) analyses of the electron density produced by the M06-D3ZERO single-point energy calculations. NBO and NRT analyses of **Ni-1c** were performed with unconstrained Lewis structures. In addition, to enable comparison with corresponding analyses reported for Grubbs-class catalysts,[19] the Lewis structure of **Ni-1c** was constrained (via the CHOOSE keyword) to explicitly consider a double Ni=C bond.

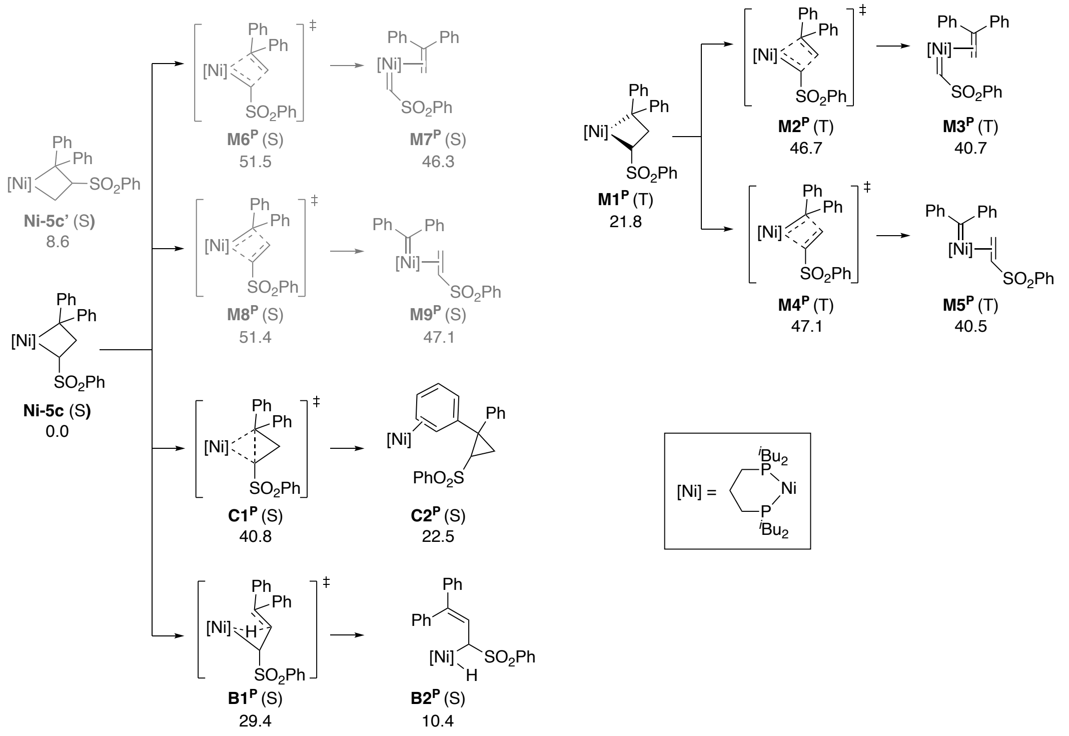

Figure S25. Overview of paths considered for reaction of **Ni-1c** with PVS. Gibbs free energies are reported in kcal/mol relative to NiCB **Ni-5c.** (S)/(T) labels indicate singlet or triplet spin state.

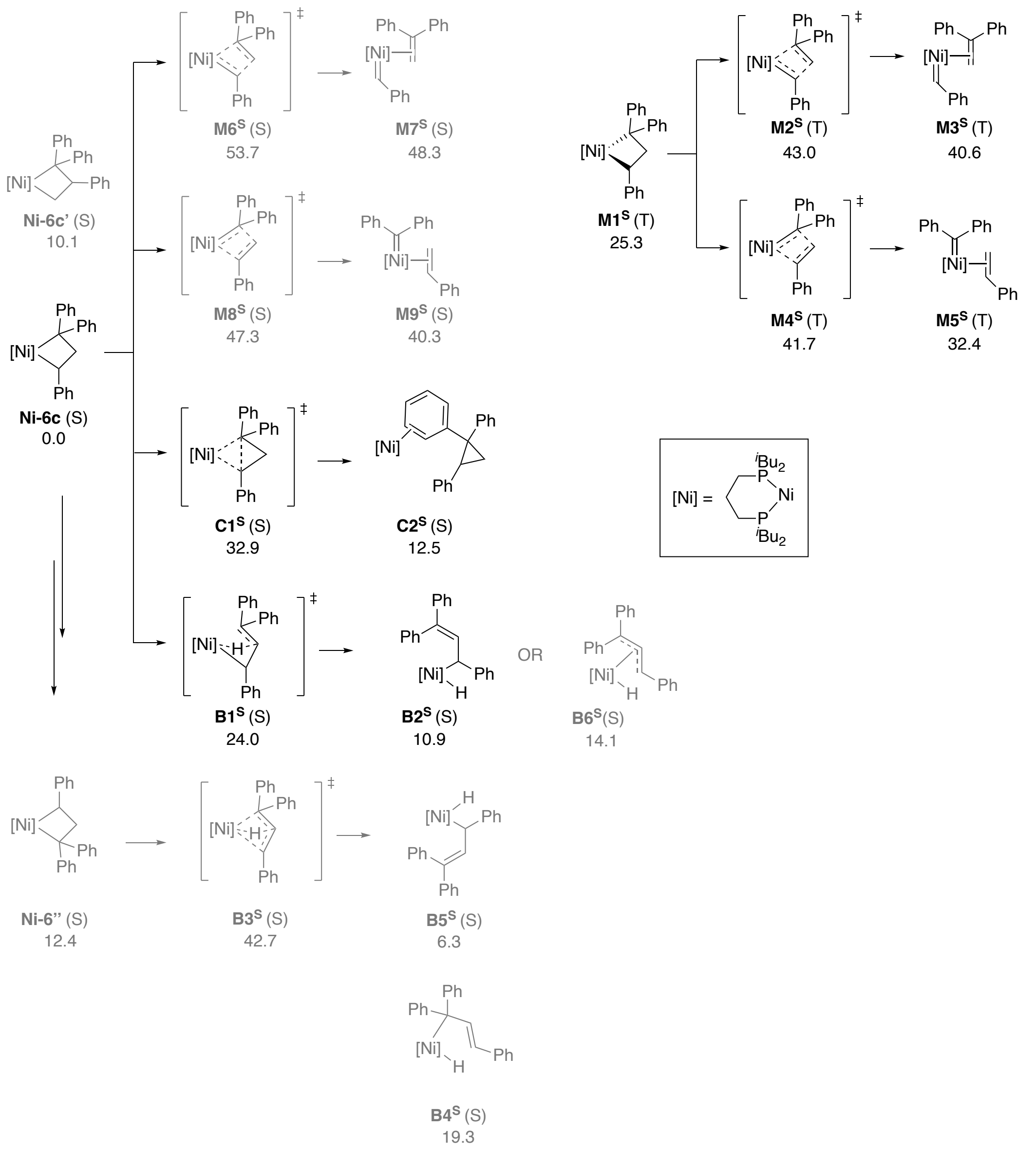

Figure S26. Overview of paths considered for reaction of **Ni-1c** with styrene. Gibbs free energies reported in kcal/mol vs NiCB **Ni-6c.** (S)/(T) labels indicate singlet or triplet spin state.

Note: The transition state (TS) for the β-H elimination pathway leading to hydride **B4^S** (see Figure S26) could not be found. Notably, this hydride is ca. 9 kcal/mol less stable than **B2^S**. This instability is presumably due in part to steric repulsion between the dibpp ligand and the phenyl groups, and in part to electronic effects favouring the secondary over the tertiary alkyl.[20] These steric and electronic effects are likely operating on any hypothetical β-H elimination TS leading to **B4^S**. Thus, any such TS is expected to have higher energy than **B1^S**, consist with the experimentally observed product. The search for such a TS with different approaches (e.g., by relaxed scans and the nudged elastic band (NEB) method)[21] repeatedly led to TS **B3^S** (see Figure S26), which has much higher energy than **B1^S** (42.7 vs 24.0 kcal/mol relative to **Ni-6c**). The intrinsic reaction coordinate (IRC) trajectories from **B3^S**, however, connect to a high-energy conformer of **Ni-6c** (**Ni-6c''**) and to hydride **B5^S**, an alternative, low-energy configuration of **B2^S**. Like **B2^S**, **B5^S** bears a secondary alkyl substituent. This reaction path is consistent with both the expected preference for a secondary-alkyl pathway[20] and the experimentally observed olefin product.

Table S20. Computational Data for Calculation of Gibbs Free Energies.

Model ID[a]	Spin Multiplicity	$E_{r2scan\text{-}3c}$ [a.u.]	$G^{298.15\,K}_{r2scan\text{-}3c}$ [a.u.]	$G^{298.15\,K}_{corr,\,r2scan\text{-}3c}$ [a.u.]	$E_{M06\text{-}D3ZERO}$ [a.u.]	$G^{298\,K\,[1M]}$ [a.u.]	$\Delta G^{298\,K\,[1M]a}$ [kcal/mol]
**Ni-1c** (S)	1	-3441.434631	-3440.738715	0.695916	-3441.432093	-3440.733160	-
**Ni-1c** (T)	3	-3441.416180	-3440.722895	0.693285	-3441.423415	-3440.727114	-
**Ni-5c**	1	-4299.638516	-4298.799735	0.838781	-4299.705800	-4298.864002	0.0
**Ni-5c'**	1	-4299.626058	-4298.788417	0.837642	-4299.691025	-4298.850366	8.6
**M6^P**	1	-4299.555374	-4298.722542	0.832832	-4299.617768	-4298.781920	51.5
**M7^P**	1	-4299.566507	-4298.731695	0.834811	-4299.628063	-4298.790236	46.3
**M8^P**	1	-4299.562412	-4298.728890	0.833522	-4299.618648	-4298.782110	51.4
**M9^P**	1	-4299.573756	-4298.739937	0.833820	-4299.625760	-4298.788923	47.1
**M1^P**	3	-4299.600810	-4298.766689	0.834120	-4299.666322	-4298.829186	21.8
**M2^P**	3	-4299.557155	-4298.727125	0.830030	-4299.622606	-4298.789559	46.7
**M3^P**	3	-4299.565463	-4298.735866	0.829597	-4299.631693	-4298.799079	40.7
**M4^P**	3	-4299.564140	-4298.732270	0.831870	-4299.623750	-4298.788863	47.1
**M5^P**	3	-4299.575225	-4298.743860	0.831366	-4299.633831	-4298.799448	40.5
**C1^P**	1	-4299.574181	-4298.738612	0.835569	-4299.637566	-4298.798980	40.8
**C2^P**	1	-4299.597194	-4298.761426	0.835768	-4299.666959	-4298.828175	22.5
**B1^P**	1	-4299.584672	-4298.751407	0.833265	-4299.653418	-4298.817136	29.4
**B2^P**	1	-4299.615046	-4298.783407	0.831639	-4299.682019	-4298.847363	10.4
**Ni-6c**	1	-3751.061736	-3750.229655	0.832081	-3751.046649	-3750.211551	0.0
**Ni-6c'**	1	-3751.048268	-3750.218072	0.830196	-3751.028713	-3750.195500	10.1
**Ni-6c''**	1	-3751.043631	-3750.211769	0.831862	-3751.026720	-3750.191842	12.4
**M6^S**	1	-3750.981640	-3750.154098	0.827542	-3750.956609	-3750.126050	53.7
**M7^S**	1	-3750.997001	-3750.170606	0.826395	-3750.964030	-3750.134619	48.3
**M8^S**	1	-3750.992680	-3750.164906	0.827774	-3750.967029	-3750.136239	47.3
**M9^S**	1	-3751.004640	-3750.177971	0.826669	-3750.977064	-3750.147379	40.3
**M1^S**	3	-3751.016316	-3750.190362	0.825953	-3751.000240	-3750.171271	25.3
**M2^S**	3	-3750.987041	-3750.162694	0.824347	-3750.970443	-3750.143079	43.0
**M3^S**	3	-3750.993910	-3750.169728	0.824183	-3750.974061	-3750.146862	40.6
**M4^S**	3	-3750.993107	-3750.167862	0.825245	-3750.973378	-3750.145116	41.7
**M5^S**	3	-3751.005406	-3750.181540	0.823866	-3750.986757	-3750.159875	32.4
**C1^S**	1	-3751.011001	-3750.180796	0.830205	-3750.992277	-3750.159055	32.9
**C2^S**	1	-3751.036130	-3750.205540	0.830590	-3751.025210	-3750.191603	12.5
**B1^S**	1	-3751.018821	-3750.192964	0.825856	-3751.002255	-3750.173382	24.0
**B2^S**	1	-3751.038618	-3750.212897	0.825721	-3751.022955	-3750.194217	10.9
**B6^S**	1	-3751.040502	-3750.214572	0.825929	-3751.018101	-3750.189155	14.1
**B3^S**	1	-3751.001438	-3750.174966	0.826472	-3750.973000	-3750.143511	42.7
**B4^S**	1	-3751.022595	-3750.197819	0.824776	-3751.008611	-3750.180818	19.3
**B5^S**	1	-3751.042503	-3750.217210	0.825292	-3751.029749	-3750.201440	6.3

$C(Ph)_2$	1	-	-	-	-501.122225	-	-
$C(Ph)_2$	3	-	-	-	-501.114776	-	-
Ni-dibpp	1	-	-	-	-2940.163452	-	-
Ni-dibpp	3	-	-	-	-2940.146466	-	-

[a]Computed using the corresponding MCB as reference.

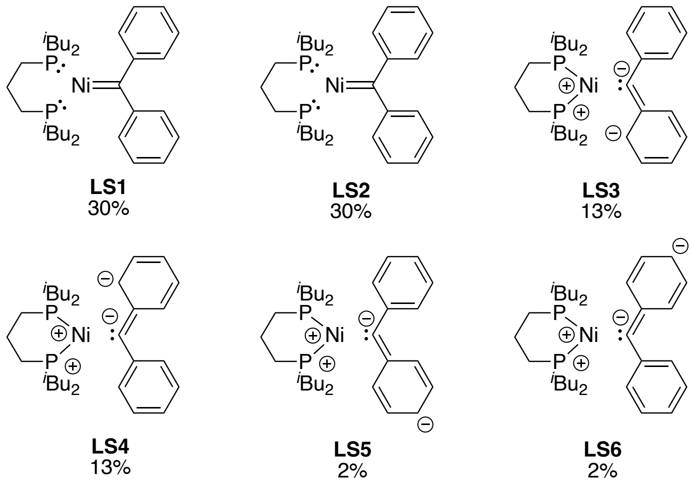

Figure S27. The six most important (highest-weight) Lewis structures of **Ni-1c** and their weight in the resonance expansion as resulting from natural resonance theory (NRT) analysis using a delocalization list energy threshold of 10 kcal/mol (via the keyword NRTE2).

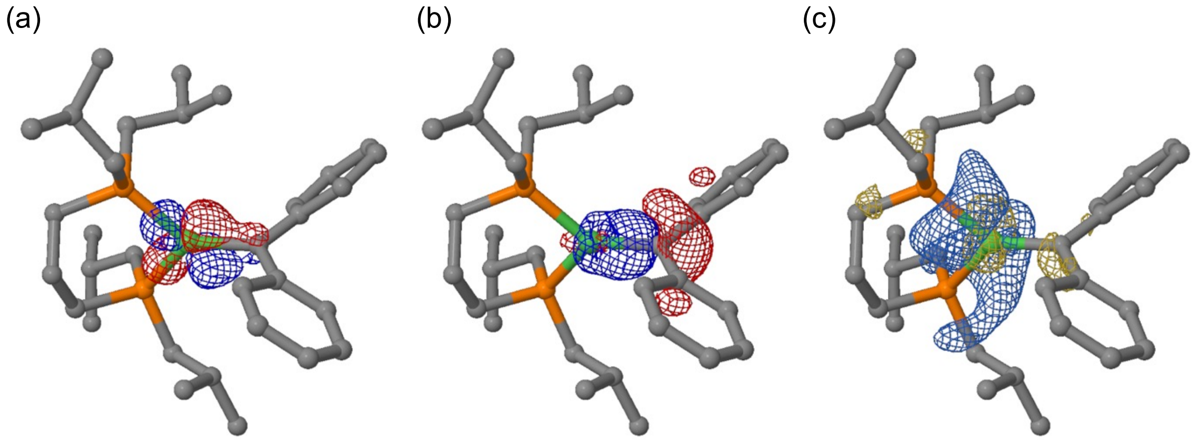

Figure S28. Molecular representation Natural Bond Orbital (NBO) isosurfaces (cutoff 0.12) for **Ni-1c**. (a) π-Symmetry bonding NBO (occupancy 1.88). (b) Donating lone pair on carbon (occupancy 1.63). (c) Acceptor orbital on Ni (occupancy 0.46).

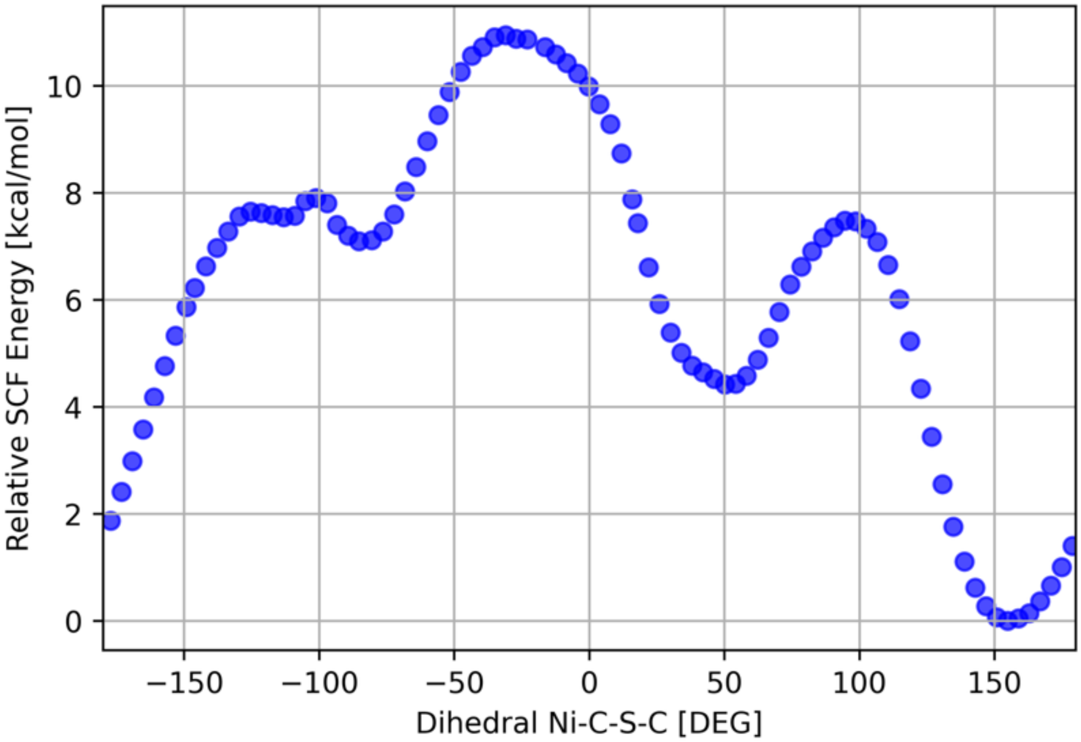

Figure S29. Relative r²scan-3c energy resulting from a relaxed torsional scan around the C–SO_2Ph bond in **Ni-5c**. The energy values are relative to the lowest energy, which corresponds to the conformation of **Ni-5c**.

S5. References

(1) Rong, J.; Li, H.; Fu, R.; Sun, W.; Loh, T.-P.; Jiang, Y., Cleavage and Reassembly C≡C Bonds of Ynones to Access Highly Functionalized Ketones. *ACS Catal.* **2020**, *10*, 3664–3669.

(2) Xie, P.; Wang, J.; Liu, Y.; Fan, J.; Wo, X.; Fu, W.; Sun, Z.; Loh, T.-P., Water-promoted C-S bond formation reactions. *Nat. Commun.* **2018**, *9*, 1321.

(3) Yin, G.; Kalvet, I.; Englert, U.; Schoenebeck, F., Fundamental Studies and Development of Nickel-Catalyzed Trifluoromethylthiolation of Aryl Chlorides: Active Catalytic Species and Key Roles of Ligand and Traceless MeCN Additive Revealed. *J. Am. Chem. Soc.* **2015**, *137*, 4164–4172.

(4) Clevenger, A. L.; Stolley, R. M.; Staudaher, N. D.; Al, N.; Rheingold, A. L.; Vanderlinden, R. T.; Louie, J., Comprehensive Study of the Reactions Between Chelating Phosphines and $Ni(cod)_2$. *Organometallics* **2018**, *37*, 3259–3268.

(5) Lee, M.; Ren, Z.; Musaev, D. G.; Davies, H. M. L., Rhodium-Stabilized Diarylcarbenes Behaving as Donor/Acceptor Carbenes. *ACS Catal.* **2020**, *10*, 6240–6247.

(6) Miao, T.; Xia, D.; Li, Y.; Li, P.; Wang, L., Direct difunctionalization of activated alkynes via domino oxidative benzylation/1,4-aryl migration/decarboxylation reactions under metal-free conditions. *Chem. Commun.* **2016**, *52*, 3175–3178.

(7) Lockwood, T. E.; Angeloski, A., DGet! An open source deuteration calculator for mass spectrometry data. *J. Cheminf.* **2024**, *16*, 36.

(8) Park, J. H.; Chung, T. S.; Hipwell, V. M.; Rivera, E.; Garcia-Garibay, M. A., Transient Kinetics and Quantum Yield Studies of Nanocrystalline α-Phenyl-Substituted Ketones: Sorting Out Reactions from Singlet and Triplet Excited States. *J. Am. Chem. Soc.* **2018**, *140*, 8192–8197.

(9) Pettersen, E. F.; Goddard, T. D.; Huang, C. C.; Couch, G. S.; Greenblatt, D. M.; Meng, E. C.; Ferrin, T. E., UCSF Chimera—A visualization system for exploratory research and analysis. *J. Comput. Chem.* **2004**, *25*, 1605–1612.

(10) Spartan 24, Wavefunction, Inc.: Irvine, CA, 2024.

(11) Halgren, T. A., Merck molecular force field. I. Basis, form, scope, parameterization, and performance of MMFF94. *J. Comput. Chem.* **1996**, *17*, 490–519.

(12) Grimme, S.; Hansen, A.; Ehlert, S.; Mewes, J.-M., r^2SCAN-3c: A "Swiss army knife" composite electronic-structure method. *J. Chem. Phys.* **2021**, *154*, 064103.

(13) Neese, F., Software update: The ORCA program system—Version 5.0. *Wiley Interdiscip. Rev. Comput. Mol. Sci.* **2022**, *12*, e1606.

(14) Zhao, Y.; Truhlar, D. G., The M06 suite of density functionals for main group thermochemistry, thermochemical kinetics, noncovalent interactions, excited states, and transition elements: two new functionals and systematic testing of four M06-class functionals and 12 other functionals. *Theor. Chem. Acc.* **2008**, *120*, 215–241.

(15) Grimme, S.; Ehrlich, S.; Goerigk, L., Effect of the damping function in dispersion corrected density functional theory. *J. Comput. Chem.* **2011**, *32*, 1456–1465.

(16) Weigend, F.; Furche, F.; Ahlrichs, R., Gaussian basis sets of quadruple zeta valence quality for atoms H–Kr. *J. Chem. Phys.* **2003**, *119*, 12753–12762.

(17) Neese, F., An improvement of the resolution of the identity approximation for the formation of the Coulomb matrix. *J. Comput. Chem.* **2003**, *24*, 1740–1747.

(18) Glendening, E. D.; Badenhoop, J. K.; Reed, A. E.; Carpenter, J. E.; Bohmann, J. A.; Morales, C. M.; Karafiloglou, P.; Landis, C. R.; Weinhold, F. *NBO*, 7.0; Theoretical Chemistry Institute, University of Wisconsin: Madison, WI, 2018.

(19) Occhipinti, G.; Jensen, V. R., Nature of the Transition Metal-Carbene Bond in Grubbs Olefin Metathesis Catalysts. *Organometallics* **2011**, *30*, 3522–3529.

(20) Harvey, J. N., Electronic Effects on the Stability of Isomeric Alkyl Transition Metal Compounds. *Organometallics* **2001**, *20*, 4887–4895.

(21) Ásgeirsson, V.; Birgisson, B. O.; Bjornsson, R.; Becker, U.; Neese, F.; Riplinger, C.; Jónsson, H., Nudged Elastic Band Method for Molecular Reactions Using Energy-Weighted Springs Combined with Eigenvector Following. *J. Chem. Theory Comput.* **2021**, *17*, 4929–4945.
